# Predictive validity of the simplified Radiographic Assessment of Lung Edema score for mortality in critically ill patients with COVID-19

**DOI:** 10.3389/fmed.2026.1819896

**Published:** 2026-05-20

**Authors:** Son Ngoc Do, Tuan Quoc Dang, Chinh Quoc Luong, My Ha Nguyen, Dung Thi Pham, Viet Khoi Nguyen, Tan Dang Do, Thai Quoc Nguyen, Vuong Minh Nong, Khoi Hong Vo, Tan Cong Nguyen, Nhung Hong Khuat, Quynh Thi Pham, Dat Tien Hoang, Anh Diep Nguyen, Phuong Minh Nguyen, Duong Dai Cao, Dung Thuy Pham, Anh Hoang Kieu Le, Anh Diep Tran, Son Hong Nguyen, Dung Tuan Dang, Dat Tuan Nguyen, Vinh Duc Nguyen, Thuan Quang Le, Hung Duc Ngo, Dung Van Nguyen, Thach The Pham, Dung Tien Nguyen, Nguyen Trung Nguyen, Lan Tuong Vu, Nhung Thi Huynh, Nga Thu Phan, Cuong Duy Nguyen, Cuong Duy Do, Chi Van Nguyen, Giap Van Vu, Co Xuan Dao

**Affiliations:** 1 Center for Critical Care Medicine, Bach Mai Hospital, Hanoi, Vietnam; 2 Department of Emergency and Critical Care Medicine, Faculty of Medicine, VNU University of Medicine and Pharmacy, Vietnam National University, Hanoi, Vietnam; 3 Department of Emergency and Critical Care Medicine, Hanoi Medical University, Hanoi, Vietnam; 4 Neuro Intensive Care Department, National Institute of Neuroscience, Bach Mai Hospital, Hanoi, Vietnam; 5 Department of Health Organization and Management, Faculty of Public Health, Thai Binh University of Medicine and Pharmacy, Hung Yen, Vietnam; 6 Department of Nutrition and Food Safety, Faculty of Public Health, Thai Binh University of Medicine and Pharmacy, Hung Yen, Vietnam; 7 Institute of Diagnostic and Interventional Radiology, Bach Mai Hospital, Hanoi, Vietnam; 8 Department of Radiology, Hanoi Medical University, Hanoi, Vietnam; 9 Bach Mai Institute for Tropical Medicine, Bach Mai Hospital, Hanoi, Vietnam; 10 Emergency Neurology Room, National Institute of Neuroscience, Bach Mai Hospital, Hanoi, Vietnam; 11 Department of Neurology, Hanoi Medical University, Hanoi, Vietnam; 12 Department of Neurology, Faculty of Medicine, VNU University of Medicine and Pharmacy, Vietnam National University, Hanoi, Vietnam; 13 Intensive Care Unit, University Medical Center Ho Chi Minh City, University of Medicine and Pharmacy at Ho Chi Minh City, Ho Chi Minh City, Vietnam; 14 Department of Intensive Care and Poison Control, Ha Dong General Hospital, Hanoi, Vietnam; 15 Stroke Center, Bach Mai Hospital, Hanoi, Vietnam; 16 Hanoi Medical University, Hanoi, Vietnam; 17 Hanoi - Amsterdam High School for the Gifted, Hanoi, Vietnam; 18 The Olympia Schools, Hanoi, Vietnam; 19 Center for Emergency Medicine, Bach Mai Hospital, Hanoi, Vietnam; 20 Poison Control Center, Bach Mai Hospital, Hanoi, Vietnam; 21 Department of Emergency and Critical Care, Hanoi Medical University Hospital, Hanoi Medical University, Hanoi, Vietnam; 22 Department of Internal Medicine, Faculty of Medicine, VNU University of Medicine and Pharmacy, Vietnam National University, Hanoi, Vietnam; 23 Department of Emergency and Critical Care Medicine, Thai Binh University of Medicine and Pharmacy, Hung Yen, Vietnam; 24 Department of Infectious Diseases, Faculty of Medicine, VNU University of Medicine and Pharmacy, Vietnam National University, Hanoi, Vietnam; 25 Respiratory Center, Bach Mai Hospital, Hanoi, Vietnam; 26 Department of Internal Medicine, Hanoi Medical University, Hanoi, Vietnam

**Keywords:** acute respiratory distress syndrome, chest X-ray severity score, COVID-19, intensive care units, mortality, prognostic factors

## Abstract

**Background:**

Early and precise identification of COVID-19 patients at high risk of mortality is vital for guiding treatment decisions. This study evaluated the predictive validity of the simplified Radiographic Assessment of Lung Edema (RALE) score, derived from chest X-rays (CXRs), for hospital mortality among critically ill patients with Delta-variant COVID-19.

**Methods:**

We conducted a retrospective observational study of 105 critically ill adults with confirmed Delta-variant COVID-19 at an intensive care center in Ho Chi Minh City, Vietnam, between 30 July and 15 October 2021. We calculated the simplified RALE score (range 0–8) from admission frontal CXRs. We evaluated predictive performance using the area under the receiver operating characteristic curve (AUROC). We used multivariable logistic regression to assess associations between the simplified RALE score and hospital mortality, adjusting for baseline confounders (inter-hospital transfers, demographics, comorbidities, clinical and laboratory parameters, and gas exchange data) and mediators (respiratory support, adjunctive therapies, and in-hospital complications).

**Results:**

The cohort averaged 60.5 years of age (40% male), and 79% died in the hospital. Bilateral lung opacities appeared in 99% of patients, and the median simplified RALE score reached 8.0 (IQR 6.0–8.0). The simplified RALE score discriminated mortality well (AUROC 0.747, 95% confidence interval [CI] 0.617–0.877), closely matching SOFA (0.747) and CURB-65 (0.776) scores. In baseline-adjusted models, both the simplified RALE (adjusted odds ratio [AOR] 2.170, 95% CI 1.353–3.482) and CURB-65 scores (AOR 4.316, 95% CI 1.684–11.059) independently predicted hospital mortality. Even after further adjusting for mediators, the simplified RALE score (AOR 1.934, 95% CI 1.220–3.066) remained an independent predictor.

**Conclusion:**

The simplified RALE score performed comparably to SOFA and CURB-65 scores in predicting hospital mortality and remained an independent predictor across all multivariable analyses. These findings support the use of the simplified RALE score as a practical tool for risk stratification in critically ill patients with Delta variant COVID-19, particularly in high-burden and resource-limited settings. Further studies in larger and more diverse populations are warranted to confirm these findings and facilitate broader clinical implementation. This study was previously presented in abstract form at: LIVES 2024, 37th Annual Congress of the European Society of Intensive Care Medicine; October 7–9, 2024; Barcelona, Spain. Do SN, Luong CQ, Nguyen MH, Pham DT, Khuat NH, Pham QT, et al. Predictive validity of the simplified Radiographic Assessment of Lung Edema score for mortality in critically ill COVID-19 patients with the B.1.617.2 (Delta) variant in Vietnam: a retrospective observational study. Intensive Care Med Exp. (2024) 12(Suppl 1):87. doi: 10.1186/s40635-024-00658-z.

## 1 Introduction

Since the first reports of coronavirus disease 2019 (COVID-19) cases from Wuhan, a city in the Hubei Province of China, at the end of 2019, cases have emerged across all continents (1–3). As of 4 February 2024, over 770 million confirmed cases of COVID-19 have been reported globally (4). However, the actual burden of disease is likely underestimated, as only a portion of acute infections are diagnosed and officially documented. During the pandemic, many countries experienced limited access to diagnostic tools, particularly amid surging case numbers. Therefore, it becomes crucial to correctly categorize COVID-19 patients based on the severity of their symptoms for efficient resource distribution (5). Specifically, peripheral blood oxygen saturation (SpO_2_) is among the first clinical parameters assessed upon hospital admission; it often reflects the degree of lung function impairment. The requirement to transfer patients with COVID-19 to an intensive care unit (ICU) depends mainly on their SpO_2_ and concurrent comorbidities (6). Besides hypoxemia, severe COVID-19 patients are characterized by dyspnoea, tachypnoea, and bilateral pulmonary infiltrates (7). Among these patients, those who require respiratory organ support (high-flow nasal oxygen with a flow rate of ≥ 30 liters per minute and a fraction of inspired oxygen of ≥ 0.4 or non-invasive or invasive ventilation) or cardiovascular organ support (vasopressors or inotropes) in an ICU are classified as being critically ill (8). Hence, some specialists propose that imaging could be beneficial in further assessing this requirement.

Imaging techniques, particularly chest X-rays (CXRs), have been proposed to assess the severity of COVID-19 and predict patient outcomes. Scoring systems, such as the Brixia score (9) and a semiquantitative CXR severity score (10), evaluate lung abnormalities using an 18-point scale across six lung zones. While these systems may lack sensitivity in the early stages of infection, they exhibit moderate to strong interobserver agreement and remain valuable for identifying the rapid progression of pulmonary disease, especially in ICU patients (9, 10). Additionally, previous studies that adapted and simplified the Radiographic Assessment of Lung Edema (RALE) (11) to a simplified RALE score demonstrate its utility in quantifying pulmonary involvement and predicting patient mortality (12–15). Abnormal findings, such as consolidation and ground-glass opacities, are commonly distributed bilaterally and peripherally, peaking around 10 to 12 days after symptom onset (12). Although less sensitive than real-time reverse transcription polymerase chain reaction (RT-PCR) and computed tomography (CT), CXR remains a valid, accessible tool for initial screening and monitoring disease progression in critically ill patients (12). However, data on the external validity of the CXR severity and simplified RALE scores are still limited. Accordingly, this study aimed to evaluate the predictive validity of the simplified RALE score for hospital mortality among critically ill COVID-19 patients in Vietnam.

## 2 Materials and methods

### 2.1 Source of data

We conducted a retrospective observational study of critically ill adult patients with COVID-19 caused by the Delta variant, after obtaining approval from the Bach Mai Hospital (BMH) Scientific and Ethics Committees (Approval number: 3412/QĐ-BVBM, issued 14 December 2021). The study included patients admitted to an Intensive Care Center for the Treatment of Critically Ill COVID-19 Patients in Ho Chi Minh City (HCMC), Vietnam, between 30 July and 15 October 2021. This facility, a 360-bed field hospital, was affiliated with BMH in Hanoi, Vietnam. BMH, designated by the Ministry of Health (MOH) as the central hospital in northern Vietnam, (16) is a large general hospital with 3,200 beds.

In July 2021, HCMC experienced a severe outbreak of the Delta variant of SARS-CoV-2, leading to a critical shortage of ICU capacity for patients with severe illness (15, 17, 18). In response, on 30 July 2021, the MOH established four Intensive Care Center for the Treatment of Critically Ill Patients with COVID-19. These centers received patients referred from lower-level hospitals within the city. One such facility, managed by BMH, was designated Field Hospital Number 16. At its peak, the hospital operated with 500 beds, but this capacity was later reduced to 360 beds. As COVID-19 cases declined, Field Hospital Number 16 ceased operations on 30 April 2022.

### 2.2 Participants

This study included eligible patients aged 18 years or older who were critically ill with COVID-19 and presented to the study center. We defined a case of COVID-19 as a person with laboratory confirmation of COVID-19 infection, regardless of clinical or epidemiological criteria (19, 20). Laboratory confirmation of COVID-19 infection was based on a positive RT-PCR test using samples obtained from the respiratory tract by nasopharyngeal swab, throat or mouth swab, or tracheal lavage fluid (21). We excluded patients who did not have CXRs between hospital admission and the end of the first day.

The study was conducted during the peak of the Delta variant epidemic in Vietnam. Although genomic sequencing was not performed on individual patient samples to confirm variant status, contemporaneous genomic surveillance data from Ho Chi Minh City indicated that nearly all circulating SARS-CoV-2 cases during this period were attributable to a single Delta sublineage (AY.57) (22). Consequently, the study population was considered virologically relatively homogeneous with respect to the infecting variant.

### 2.3 Data collection

We used a unified case record form (CRF) to collect data on standard variables from medical records. Data entry was conducted using KoboToolbox, an open-source digital data collection platform developed by the Harvard Humanitarian Initiative in Cambridge, Massachusetts. This platform supports real-time validation, thereby minimizing data entry errors and enhancing accuracy. To ensure patient confidentiality, no identifying information was recorded in the study database.

### 2.4 Outcome measures

The primary outcome was hospital mortality, which we defined as death from any cause during the hospitalization. We also examined the secondary outcomes, such as complications (e.g., ARDS) and hospital lengths of stay.

### 2.5 Predictor measures

The CRF included exposure and confounding variables mainly based on the COVID-19 Treatment Guidelines of the National Institutes of Health (23) and collected by fully trained clinicians, such as information on:

We defined the exposure variables as follows: the simplified RALE score, the Sequential Organ Failure Assessment (SOFA) score (24), the Acute Physiology and Chronic Health Evaluation II (APACHE II) score (25), and the CURB-65 criteria (26), which include Confusion, a Urea level greater than 7 mmol/L, a Respiratory rate of 30 breaths per minute or more, and Blood pressure readings of less than 90 mm Hg (systolic) or less than 60 mm Hg (diastolic), along with an age of 65 years or older (26). These scores were collected within the time frame from admission until 24 h later. Additionally, upon admission, we recorded the interleukin-6 (IL-6) level and the arterial oxygen partial pressure to inspired oxygen fraction ratio (PaO_2_/FiO_2_ ratio) as part of the exposure variables. At our study site, radiologists performed bedside CXRs for severely and critically ill COVID-19 patients using a compact, portable device (FDR nano, Fujifilm) in a single frontal anteroposterior projection. The X-ray tube was mounted over each patient, and the image receptor was placed beneath each patient to minimize movement and reduce infection risk. The scoring used a two-step process from admission through the first hospital day: a third-year radiology resident reviewed all CXRs and calculated the simplified RALE score; then, a thoracic radiologist with ten years of experience independently validated the findings. Both reviewers were blinded to clinical data and patient outcomes. They resolved discrepancies directly to reach a consensus. We adapted and simplified the RALE score (11) to create a simplified RALE score for assessing the severity of COVID-19 based on lung involvement. The radiological classification details from the RALE score are available in Supplementary Figure 1. This approach has also been used in prior published studies to quantify the extent of pulmonary infection in COVID-19 cases (12–15, 20). In a frontal CXR, the simplified RALE scoring system assigns a score to each lung, ranging from 0 to 4. This score reflects the attendance of specific abnormalities. The system divides each lung into four zones, and abnormalities such as consolidation, ground-glass opacification, and reticular interstitial thickening are assigned a score of 1 in each zone (Supplementary Figure 2). A score of 0 indicates no involvement, while a score of 1 corresponds to less than 25% involvement, a score of 2 represents 25% to less than 50% involvement, a score of 3 signifies 50% to less than 75% involvement, and a score of 4 indicates 75% or greater involvement. The overall severity score is the sum of points from both lungs, with a maximum possible score of 8 (15). Based on the simplified RALE score upon admission, the patients were classified into three severity groups as follows: mild (a score of 1 to 2), moderate (a score of 3 to 6), and severe (a score of 7 to 8). Serum IL-6 was measured using the Elecsys IL-6 immunoassay (Roche^§^ Cobas E411 analyzer), an *in vitro* diagnostic test for quantitatively determining IL-6 in human serum and plasma. The measuring range is from 1.5 to 5,000 pg/mL. Upon admission, we collected data on the IL-6 levels and input it into a CRF. In the present study, we defined the IL-6 level as 5,000 pg/mL when values were above the measuring range (> 5,000 pg/mL).

We identified confounding factors as variables collected on a unified CRF, which consisted of four sections as follows:

(i) The first section focused on baseline characteristics, such as inter-hospital care (e.g., prior hospitalization, inter-hospital airway, inter-hospital oxygen), demographics (i.e., age and gender), documented comorbidities (e.g., cerebrovascular disease, chronic cardiac failure, coronary artery disease/myocardial infarction, hypertension, chronic obstructive pulmonary disease/asthma, other chronic pulmonary disease, tuberculosis, chronic renal failure, and diabetes mellitus), and details of admission. We also used the 19 comorbidity categories to compute the Charlson Comorbidity Index (CCI) score, which measures the predicted mortality rate based on the presence of comorbidities (27). A score of zero indicates that no comorbidities were detected; the higher the score, the higher the expected mortality rate is (27–29).
(ii) The second section comprised characteristics upon admission, such as vital signs (e.g., heart rate, respiration rate, arterial blood pressure, and body temperature), laboratory parameters, CXR findings (e.g., bilateral opacities, number of involved quadrants), gas exchange [e.g., arterial oxygen partial pressure (PaO_2_), arterial carbon dioxide partial pressure (PaCO_2_), blood potential hydrogen (pH), and arterial oxygen partial pressure to inspired oxygen fraction ratio (PaO_2_/FiO_2_)].
(iii) The third section captured life-sustaining treatments provided during the ICU stay, such as respiratory support on the first day of admission (e.g., oxygen supplements and mechanical ventilation) and adjunctive therapies (e.g., prone positioning, recruitment maneuvers, extracorporeal membrane oxygenation, antiviral drugs, antibiotics, corticosteroids, heparin, antiplatelet drugs, novel oral anticoagulants, recombinant humanized anti-IL-6 receptor monoclonal antibody, continuous sedation, continuous neuromuscular blocking agents, renal replacement therapy, cytokine adsorption therapy, tracheostomy, and inhaled vasodilators).
(iv) The fourth section is concerned with complications [e.g., hospital-acquired pneumonia (HAP), acute respiratory distress syndrome (ARDS), diffuse alveolar damage, secondary bacterial infections, sepsis and septic shock, cardiac injury, arrhythmia, acute kidney injury, liver dysfunction, multi-organ failure, thromboembolism, gastrointestinal bleeding, and pneumothorax/pneumomediastinum] and clinical outcomes (e.g., hospital mortality). HAP is defined as pneumonia that occurs 48 h or more after admission and does not appear to be incubating at the time of admission (30). To diagnose and classify ARDS, we applied the Berlin criteria, which categorize ARDS severity based on the PaO_2_/FiO_2_ ratio as follows: mild (200 < PaO_2_/FiO_2_ ≤ 300 mmHg), moderate (100 < PaO_2_/FiO_2_ ≤ 200 mmHg), and severe (PaO_2_/FiO_2_ ≤ 100 mmHg), with a minimum positive end-expiratory pressure (PEEP) of 5 cmH_2_O applied to the lungs at the end of each breath (31, 32). Additionally, septic shock is identified as a clinical construct of sepsis characterized by persisting hypotension requiring vasopressors to maintain a mean arterial pressure ≥ 65 mmHg, along with a serum lactate level > 2 mmol/L (18 mg/dL) despite adequate volume resuscitation (33). Lastly, thromboembolism encompasses venous thromboembolism (i.e., deep vein thrombosis and pulmonary embolism), arterial events (i.e., stroke, limb ischemia, and myocardial infarction), and microvascular thrombosis (e.g., microvascular thrombosis in the lungs) (23).

### 2.6 Statistical analyses

We used IBM^§^ SPSS^§^ Statistics 22.0 (IBM Corp., Armonk, United States of America) and Analyze-it statistical software (Analyze-it Software, Ltd., Leeds, United Kingdom) for data analysis. We presented categorical variables as numbers (no.) and percentages (%), while continuous variables were reported as median with interquartile ranges (IQR) or mean with standard deviations (SDs). We employed the Chi-squared or Fisher’s exact test to compare survival and death in the hospital across categorical variables. We used the Kolmogorov-Smirnov test to evaluate the null hypothesis that continuous variables follow a normal distribution. For continuous variables that met the criteria for normality, we used the Student’s *t*-test or one-way analysis of variance (ANOVA) to compare survival and death in the hospital. Conversely, we applied the Mann–Whitney U or Kruskal–Wallis tests for continuous variables that did not meet normality assumptions.

To evaluate the predictive performance of variables for hospital mortality among critically ill COVID-19 patients upon admission, we plotted receiver operating characteristic (ROC) curves and computed the areas under the ROC curves (AUROCs) for the simplified RALE score, SOFA score, APACHE II score, CURB-65 score, and IL-6 level. In this context, higher scores or levels indicated more positive test results. Similarly, we assessed the PaO_2_/FiO_2_ ratio using ROC curves and AUROCs, where lower ratios represented more positive test results. Optimal cut-off values for each variable, whether a score, level, or ratio, were determined using Youden’s index (sensitivity + specificity −1). We then compared the AUROCs of the simplified RALE score with those of the SOFA, APACHE II, and CURB-65 scores, as well as the IL-6 level, using Z-statistics to see which one was better at predicting hospital mortality. Additionally, we calculated correlation coefficients (Rs) using Spearman’s rho to explore the relationship between the simplified RALE score and other measures.

We used logistic regression to assess the association between key exposure variables (i.e., the simplified RALE score, SOFA score, APACHE II score, CURB-65 score, IL-6 level, and PaO_2_/FiO_2_ ratio) and hospital mortality, with adjustment for potential confounders. Univariable logistic regression was first performed for each exposure and candidate covariate to explore unadjusted associations. Candidate covariates were classified *a priori* as baseline confounders (e.g., inter-hospital transfers, demographics, comorbidities, clinical characteristics, chest X-ray findings, laboratory parameters, and gas exchange data) or potential mediators (e.g., first-day respiratory support methods, adjunctive therapies, and complications). To evaluate the effect of admission prognostic scores, levels, or ratios while minimizing over-adjustment for downstream effects, we constructed models in stages. For the baseline multivariable model, we selected candidate baseline confounders based on a univariable *P*-value ≤ 0.10 (when comparing survivors vs. non-survivors), including inter-hospital transfers (e.g., nasal cannula), demographics (e.g., age), comorbidities (e.g., CCI score), admission vitals (e.g., respiratory rate), and labs (e.g., fibrinogen), or established clinical relevance, including inter-hospital transfers [e.g., endotracheal tube (34)] and demographic variables [e.g., gender (35)]. Variables with > 10% missing data [e.g., certain admission laboratory values such as C-reactive protein (CRP)] were excluded. A full baseline model containing the exposure variable and all selected confounders was then fitted, followed by backward stepwise elimination (removal of variables with *P* ≥ 0.05 at each iteration) to retain only independent predictors. To explore potential mediator effects, we extended the baseline model in a sensitivity analysis by adding candidate mediator variables that showed a univariable association with mortality (*P* ≤ 0.10) included first-day respiratory support (e.g., high-flow nasal cannula), adjunctive therapies (e.g., antiviral drugs, continuous neuromuscular blocking agents), and complications (e.g., ARDS) or had strong clinical justification included first-day respiratory support [e.g., bag-valve-mask ventilation (36)], adjunctive therapies [e.g., renal replacement therapy (37)], and complications [e.g., HAP (38)]. Mediators with > 10% missing data (e.g., complications such as secondary bacterial infections, sepsis, and septic shock) or those causing substantial multicollinearity (e.g., mechanical ventilation, continuous sedation, diffuse alveolar damage) were excluded. The full model (exposure, baseline confounders, and mediators) was again subjected to stepwise backward elimination to identify independent predictors of hospital mortality. Model diagnostics and sensitivity analyses included assessment of multicollinearity using variance inflation factors (all VIFs < 5, considered acceptable). Model calibration was evaluated using the Hosmer–Lemeshow goodness-of-fit test, log-likelihood, pseudo-R^2^, calibration plots (observed vs. predicted probabilities), and estimation of the calibration slope and intercept. Sensitivity analyses comprised a complete-case analysis (excluding patients with any missing covariate data), which produced results consistent with the primary analysis. Odds ratios (ORs) with 95% confidence intervals (CIs) were reported for univariable analyses, while adjusted odds ratios (AORs) with 95% CIs were presented for multivariable models.

Significance levels were two-tailed for all analyses, and we considered *P* < 0.05 to be statistically significant.

## 3 Results

### 3.1 Baseline characteristics and clinical outcomes

Of 105 patients, 40.0% were men, and the mean age was 60.45 years (SD: 14.98) (Table 1). Upon admission, nearly all patients (99.0%; 100/102) exhibited bilateral lung opacities on their CXR (Table 2). These opacities were most frequently distributed across three (18.3%; 19/104) and four quadrants of lungs (74.0%; 77/104). Additionally, the patients had a high median simplified RALE score of 8.0 (IQR: 6.0–8.0) (Table 2) and a low median PaO_2_/FiO_2_ ratio of 85.0 (IQR: 65.3–119.3) (Table 2). Upon admission, an elevated median serum IL-6 level was observed (41.8 pg/mL; IQR: 20.7–41.8) (Table 2). The median SOFA score was 4.0 (IQR: 2.0–5.0), the median APACHE II score was 8.0 (IQR: 3.0–12.0), and the median CURB-65 score was 1.0 (IQR: 1.0–2.0) within the time window from admission until 24 h later (Table 2). In general, 79.0% (83/105) of the patients died in the hospital (Tables 1, 2). As shown in Tables 1, 2 and Supplementary Tables 1–5, we also compared the factors between survival and death in the hospital, such as inter-hospital care, demographics, comorbidities, vital signs, laboratory investigations, CXR findings, severity scoring systems, treatments, and complications.

**TABLE 1:** Baseline characteristics of critically ill COVID-19 patients with the Delta variant, according to hospital survivability.

Variables	All cases *n* = 105	Survived *n* = 22	Died *n* = 83	*P*-value^a^
Demographics				
Age (year), mean (SD)	60.45 (14.98)	53.82 (14.76)	62.20 (14.63)	0.019
Gender (male), no. (%)	42 (40.0)	6 (27.3)	36 (43.4)	0.171
Comorbidities				
Cerebrovascular disease, no. (%), *n* = 78	5 (6.4)	0 (0.0)	5 (7.7)	0.583
Chronic cardiac failure, no. (%), *n* = 78	4 (5.1)	2 (15.4)	2 (3.1)	0.127
Coronary artery disease/MI, no. (%), *n* = 78	4 (5.1)	1 (7.7)	3 (4.6)	0.525
Hypertension, no. (%), *n* = 78	50 (64.1)	7 (53.8)	43 (66.2)	0.528
COPD/asthma, no. (%), *n* = 78	1 (1.3)	0 (0.0)	1 (1.5)	> 0.999
Other chronic pulmonary disease, no. (%), *n* = 78	1 (1.3)	0 (0.0)	1 (1.5)	> 0.999
Tuberculosis, no. (%), *n* = 78	1 (1.3)	0 (0.0)	1 (1.5)	> 0.999
Chronic renal failure, no. (%), *n* = 78	1 (1.3)	0 (0.0)	1 (1.5)	> 0.999
Diabetes mellitus, no. (%), *n* = 78	40 (51.3)	5 (38.5)	35 (53.8)	0.311
CCI score, median (IQR), *n* = 102	2.0 (1.0–4.0)	1.0 (0.0–2.5)	2.0 (1.0–4.0)	0.023
Vital signs upon admission				
HR (beats/min), median (IQR), *n* = 99	92.0 (80.0–100.0)	90.0 (84.75–100.75)	95.0 (80.0–100.0)	0.871
RR (breaths/min), median (IQR), *n* = 97	26.0 (25.0–30.0)	25.0 (22.0–27.0)	28.0 (25.0–31.5)	0.006
Systolic BP (mmHg), median (IQR), *n* = 99	120.0 (120.0–135.0)	120.0 (120.0–136.3)	120.0 (120.0–137.5)	0.573
Diastolic BP (mmHg), median (IQR), *n* = 98	80.0 (70.0–80.0)	80.0 (73.0–80.0)	80.0 (70.0–80.0)	0.195
Body temperature (°C), median (IQR), *n* = 95	37.0 (36.8–37.0)	37.0 (37.0–37.0)	37.00 (36.8–37.0)	0.390

^a^The comparison between patients who survived and those who died in the hospital. BP, blood pressure; CCI, Charlson Comorbidity Index; COPD, chronic obstructive pulmonary disease; HR, heart rate; IQR, interquartile range; MI, myocardial infarction; no., number of patients; RR, respiration rate; SD, standard deviation.

**TABLE 2:** Laboratory investigations, gas exchange, chest X-ray findings, and severity scoring systems of critically ill COVID-19 patients with the Delta variant upon admission, according to hospital survivability.

Variables	All cases *n* = 105	Survived *n* = 22	Died *n* = 83	*P*-value^a^
Laboratory investigations				
WBC (×10^9^/L), mean (SD)	12.94 (5.73)	11.94 (4.78)	13.21 (5.95)	0.358
Hemoglobin (g/L), median (IQR)	128.0 (114.5–138.0)	122.50 (111.8–133.3)	129.00 (115.0–142.0)	0.317
Platelet count (×10^9^/L), median (IQR)	229.0 (174.5–323.0)	226.50 (183.3–285.0)	238.00 (167.0–329.0)	0.937
CRP (mg/L), median (IQR), *n* = 90	10.5 (4.8–14.0)	7.48 (2.8–13.5)	10.44 (4.9–14.1)	0.295
Glucose (mmol/L), median (IQR), *n* = 101	10.0 (7.5–14.4)	9.50 (5.8–13.8)	10.15 (7.5–14.6)	0.473
Ure (mmol/L), median (IQR), *n* = 104	6.8 (5.0–9.7)	5.35 (4.4–6.9)	7.20 (5.2–10.0)	0.005
Creatinine (μmol/L), median (IQR), *n* = 104	81.0 (68.0–91.8)	70.50 (63.8–86.0)	82.50 (68.8–96.3)	0.040
INR, median (IQR)	1.1 (1.0–1.3)	1.11 (1.0–1.3)	1.12 (1.0–1.3)	0.576
Interleukin 6 (pg/mL), median (IQR), *n* = 90	41.8 (20.7–41.8)	27.1 (7.9–96.3)	45.1 (25.4–107.1)	0.128
Gas exchange				
pH, median (IQR), *n* = 63	7.43 (7.3–7.5)	7.50 (7.4–7.5)	7.40 (7.3–7.5)	0.006
PaO_2_ (mmHg), median (IQR), *n* = 63	70.00 (58.0–83.0)	78.00 (67.0–115.0)	67.00 (58.0–81.8)	0.135
PaCO_2_ (mmHg), median (IQR), *n* = 63	39.00 (34.0–48.0)	38.00 (30.0–41.0)	38.50 (34.0–51.8)	0.224
SpO_2_ (%),median (IQR), *n* = 87	94.00 (90.0–95.0)	95.00 (94.0–97.0)	93.00 (90.0–95.0)	0.042
Chest X-ray findings				
Bilateral opacities, no. (%), *n* = 102	100 (99.0)	19 (95.0)	82 (100.0)	0.196
Number of involved quadrants, *n* = 104				< 0.001
1 quadrant, no. (%)	1 (1.0)	1 (4.8)	0 (0.0)	
2 quadrants, no. (%)	7 (6.7)	6 (28.6)	1 (1.2)	
3 quadrants, no. (%)	19 (18.3)	6 (28.6)	13 (15.7)	
4 quadrants, no. (%)	77 (74.0)	8 (38.1)	69 (83.1)	
Simplified RALE score, median (Q1–Q3), *n* = 105	8.0 (6.0–8.0)	6.0 (4.0–8.0)	8.0 (7.0–8.0)	< 0.001
Severity scoring system				
SOFA score, median (Q1–Q3), *n* = 79	4.0 (2.0–5.0)	2.0 (0.0–3.0)	4.0 (2.25–5.0)	0.002
APACHE II score, median (Q1–Q3), *n* = 75	8.0 (3.0–12.0)	7.0 (2.0–8.75)	9.0 (4.0–13.0)	0.077
CURB-65 score, median (Q1–Q3), *n* = 99	1.0 (1.0–2.0)	0.5 (0.0–1.0)	1.0 (1.0–2.0)	< 0.001

^a^The comparison between patients who survived and those who died in the hospital. APACHE II, Acute Physiology and Chronic Health Evaluation II; CRP, C-reactive protein; CURB-65, Confusion, Urea > 7 mmol/L, respiratory rate ≥ 30 breaths/min, blood pressure < 90 mm Hg (systolic) or < 60 mm Hg (diastolic), and age ≥ 65 years criteria; IL-6, interleukin 6; INR, international normalized ratio; IQR, interquartile range; no., number of patients; PaO_2_, arterial oxygen partial pressure; PaO_2_/FiO_2_, arterial oxygen partial pressure to inspired oxygen fraction ratio; pH, blood potential hydrogen; SD, standard deviation; SOFA, Sequential Organ Failure Assessment; SpO_2_, peripheral oxygen saturation; RALE, Radiographic Assessment of Lung Edema; WBC, white blood cell.

### 3.2 Overall predictive performance of simplified RALE score and other severity scoring systems

In this study, the simplified RALE score demonstrated good discriminatory ability in predicting hospital mortality, with an AUROC of 0.747 (95% CI: 0.617–0.877), a cut-off value of ≥ 5.5, a sensitivity of 93.9%, a specificity of 45.5%, and a P_*AUROC*_ < 0.001 (Table 3). Similarly, the SOFA score (AUROC: 0.747 [95% CI: 0.604–0.890], cut-off value ≥ 3.5, sensitivity: 62.5%, specificity: 80.0%, P_*AUROC*_ = 0.003) and the CURB-65 score (AUROC: 0.776 [95% CI: 0.665–0.887], cut-off value ≥ 0.5, sensitivity: 89.9%, specificity: 50.0%, P_*AUROC*_ = 0.001) also demonstrated good discriminatory performance for predicting hospital mortality (Table 3). We provide additional details on the predictive performance of other factors in critically ill COVID-19 patients with the Delta variant in Table 3.

**TABLE 3:** Diagnostic accuracy of simplified RALE, SOFA, APACHE II, CURB-65, PaO_2_/FiO_2_, and serum IL-6 in predicting hospital mortality in critically ill COVID-19 patients with Delta variant upon admission.

Variables	AUROC (95% CI)	*p*-value	Cut-off value	Sensitivity (%)	Specificity (%)
Simplified RALE score	0.747 (0.617–0.877)	< 0.001	5.5	93.9	45.5
SOFA score	0.747 (0.604–0.890)	0.003	3.5	62.5	80.0
APACHE II score	0.645 (0.504–0.785)	0.078	11.5	33.9	93.8
CURB-65 score	0.776 (0.665–0.887)	0.001	0.5	89.9	50.0
PaO_2_/FiO_2_ ratio	0.694 (0.513–0.876)	0.045	119.5 mmHg	82.4	54.5
Serum IL-6 level	0.610 (0.459–0.761)	0.128	15.8 pg/mL	84.1	42.9

APACHE, Acute Physiology and Chronic Health Evaluation; AUROC, areas under the receiver operating characteristic curve; CI, confidence interval; CURB-65, Confusion, Urea > 7 mmol/L, respiratory rate ≥ 30 breaths/min, blood pressure < 90 mm Hg (systolic) or < 60 mm Hg (diastolic), age ≥ 65 years; FiO_2_, inspired oxygen fraction; IL-6, interleukin-6; PaO_2_, arterial oxygen partial pressure; RALE, Radiographic Assessment of Lung Edema; SOFA, Sequential Organ Failure Assessment.

Furthermore, Supplementary Table 6 compares the AUROC curves of the simplified RALE with other severity scoring systems. Notably, there was no significant difference in the AUROCs for predicting hospital mortality between the simplified RALE score and the SOFA score (AUROC difference: 0.015, 95% CI: −0.117 to 0.148, Z-statistic: 0.23, *p* = 0.819) or between the simplified RALE score and the CURB-65 score (AUROC difference: 0.046, 95% CI: −0.110 to 0.203, Z-statistic: 0.58, *p* = 0.562). We also summarize the AUROC differences for other factors in critically ill COVID-19 patients with the Delta variant in Supplementary Table 6.

### 3.3 Spearman’s correlation between simplified RALE score and severity of illness

We used Spearman’s rank correlation coefficient to evaluate the relationships between the simplified RALE score and various severity scoring systems upon admission (Table 4 and Supplementary Table 7). We found a modest correlation between the simplified RALE score and the SOFA score (Rs = 0.344; *p* = 0.002), the APACHE II score (Rs = 0.231; *p* = 0.046), the CURB-65 score (Rs = 0.235; *p* = 0.019), and the serum IL-6 level (Rs = 0.226; *p* = 0.032). However, we observed no correlation between the simplified RALE score and the PaO_2_/FiO_2_ ratio (Rs = −0.158; *p* = 0.220). Additional Spearman’s correlation results for other severity scoring systems upon admission are available in Supplementary Table 7.

**TABLE 4:** Spearman’s correlation between the simplified RALE score and the severity scoring systems upon admission.

		Simplified RALE	SOFA	APACHE II	CURB-65	PaO_2_/FiO_2_	Serum IL-6
Simplified RALE	Rs value	1.000	0.344	0.231	0.235	−0.158	0.226
	*P*-value	NA	0.002	0.046	0.019	0.220	0.032
	*N*	105	79	75	99	62	90

APACHE II, Acute Physiology and Chronic Health Evaluation II; CURB-65, Confusion, Urea > 7 mmol/L, respiratory rate ≥ 30 breaths/min, blood pressure < 90 mm Hg (systolic) or < 60 mm Hg (diastolic), and age ≥ 65 years criteria; IL-6, interleukin 6; *N*, number of patients; NA, not available; PaO_2_/FiO_2_, the ratio of partial pressure of oxygen in arterial blood to the fraction of inspiratory oxygen concentration; Rs, correlation coefficients; SOFA, Sequential Organ Failure Assessment; RALE, Radiographic Assessment of Lung Edema.

### 3.4 Association of simplified RALE score with hospital mortality

In the univariable analyses, higher simplified RALE scores (OR: 1.938; 95% CI: 1.393–2.697; *p* < 0.001), SOFA scores (OR: 1.680; 95% CI: 1.158–2.438; *p* = 0.006), and CURB-65 scores (OR: 4.436; 95% CI: 1.940–10.146; *p* < 0.001) were identified as significant predictors of hospital mortality (Tables 5, 6). In contrast, the APACHE II score, PaO_2_/FiO_2_ ratio, and IL-6 level did not show significant associations with hospital mortality. Multivariable analyses that included only baseline variables demonstrated that both a higher simplified RALE score (AOR: 2.170; 95% CI: 1.353–3.482; *p* = 0.001) and a higher CURB-65 score (AOR: 4.316; 95% CI: 1.684–11.059; *p* = 0.002) remained independently associated with an increased risk of hospital mortality (Table 5). However, when mediator variables, such as first-day respiratory support methods, adjunctive therapies, and complications, were included in the model, only the simplified RALE score remained an independent predictor of hospital mortality (AOR: 1.934; 95% CI: 1.220–3.066; *p* = 0.005) (Table 6). Additional factors associated with hospital mortality, after adjustment for baseline and/or mediator confounders, are detailed in Tables 5, 6 and in Supplementary Tables 8–31.

**TABLE 5:** Association of severity scoring systems with hospital mortality in critically ill COVID-19 patients with Delta variant upon admission, adjusted for baseline confounders.

Factors	Univariable logistic regression analyses				Multivariable logistic regression analyses			
	OR	95% CI for OR		*p*-value	AOR	95% CI for AOR		*p*-value
		Lower	Upper			Lower	Upper	
Simplified RALE score	1.938	1.393	2.697	< 0.001	2.170	1.353	3.482	0.001
Simplified RALE score ≥ 5.5^a^	13.000	3.786	44.637	< 0.001	12.977	2.562	65.719	0.002
SOFA score	1.680	1.158	2.438	0.006	1.417	0.939	2.139	0.097
SOFA score ≥ 3.5^a^	6.667	1.707	26.042	0.006	4.450	0.801	24.726	0.088
APACHE II score	1.117	0.988	1.261	0.077	NA	NA	NA	NA
APACHE II score ≥ 11.5^a^	7.692	0.947	62.490	0.056	NA	NA	NA	NA
CURB-65 score	4.436	1.940	10.146	< 0.001	4.316	1.684	11.059	0.002
CURB-65 score ≥ 0.5^a^	8.875	2.835	27.786	< 0.001	6.283	1.568	25.185	0.009
PaO_2_/FiO_2_ ratio	0.993	0.983	1.002	0.140	NA	NA	NA	NA
PaO_2_/FiO_2_ ratio ≥ 119.5 mmHg^a^	0.179	0.045	0.716	0.015	0.166	0.037	0.736	0.018
IL-6 level	1.002	0.997	1.007	0.334	NA	NA	NA	NA
IL-6 level ≥ 15.8 pg/mL^a^	3.955	1.345	11.623	0.012	NA	NA	NA	NA

See Supplementary Tables 8–19 for additional information. ^a^To indicate the best cut-off value determined by analyzing the receiver operator characteristic curve of severity scoring systems for predicting hospital mortality. AOR, adjusted odds ratio; APACHE II score, Acute Physiology and Chronic Health Evaluation II score; CI, confidence interval; CURB-65 score, Confusion, Urea > 7 mmol/L, respiratory rate ≥ 30 breaths/min, blood pressure < 90 mm Hg (systolic) or < 60 mm Hg (diastolic), age ≥ 65 years; IL-6, interleukin 6; NA, not available; OR, odds ratio; PaO_2_/FiO_2_ ratio, arterial oxygen partial pressure to inspired oxygen fraction ratio; simplified RALE score, simplified Radiographic Assessment of Lung Edema score; SOFA score, Sequential Organ Failure Assessment score.

**TABLE 6:** Association of severity scoring systems with hospital mortality in critically ill COVID-19 patients with Delta variant upon admission, adjusted for baseline and mediator confounders.

Factors	Univariable logistic regression analyses				Multivariable logistic regression analyses			
	OR	95% CI for OR		*p*-value	AOR	95% CI for AOR		*p*-value
		Lower	Upper			Lower	Upper	
Simplified RALE score	1.938	1.393	2.697	< 0.001	1.934	1.220	3.066	0.005
Simplified RALE score ≥ 5.5^a^	13.000	3.786	44.637	< 0.001	6.665	1.020	43.560	0.048
SOFA score	1.680	1.158	2.438	0.006	NA	NA	NA	NA
SOFA score ≥ 3.5^a^	6.667	1.707	26.042	0.006	NA	NA	NA	NA
APACHE II score	1.117	0.988	1.261	0.077	NA	NA	NA	NA
APACHE II score ≥ 11.5^a^	7.692	0.947	62.490	0.056	NA	NA	NA	NA
CURB-65 score	4.436	1.940	10.146	< 0.001	NA	NA	NA	NA
CURB-65 score ≥ 0.5^a^	8.875	2.835	27.786	< 0.001	NA	NA	NA	NA
PaO_2_/FiO_2_ ratio	0.993	0.983	1.002	0.140	NA	NA	NA	NA
PaO_2_/FiO_2_ ratio ≥ 119.5 mmHg^a^	0.179	0.045	0.716	0.015	NA	NA	NA	NA
IL-6 level	1.002	0.997	1.007	0.334	NA	NA	NA	NA
IL-6 level ≥ 15.8 pg/mL^a^	3.955	1.345	11.623	0.012	NA	NA	NA	NA

See Supplementary Tables 20–31 for additional information. ^a^To indicate the best cut-off value determined by analyzing the receiver operator characteristic curve of severity scoring systems for predicting hospital mortality. AOR, adjusted odds ratio; APACHE II score, Acute Physiology and Chronic Health Evaluation II score; CI, confidence interval; CURB-65 score, Confusion, Urea > 7 mmol/L, respiratory rate ≥ 30 breaths/min, blood pressure < 90 mm Hg (systolic) or < 60 mm Hg (diastolic), age ≥ 65 years; IL-6, interleukin 6; NA, not available; OR, odds ratio; PaO_2_/FiO_2_ ratio, arterial oxygen partial pressure to inspired oxygen fraction ratio; simplified RALE score, simplified Radiographic Assessment of Lung Edema score; SOFA score, Sequential Organ Failure Assessment score.

## 4 Discussion

This study demonstrated that the simplified RALE score is an effective predictor of hospital mortality, with good discriminatory ability (Table 3). The SOFA and CURB-65 scores also performed well in differentiating outcomes (Table 3). Notably, there were no significant differences between the AUROC curves for the simplified RALE, SOFA, and CURB-65 scores (see Supplementary Table 6). The simplified RALE score showed modest correlations with both SOFA and CURB-65 scores (Table 4). Univariable logistic regression analyses indicated that higher scores on all three measures were associated with a higher risk of hospital mortality (Tables 5, 6). In baseline multivariable models adjusting for the same set of baseline confounders, both the simplified RALE and CURB-65 scores remained independently associated with hospital mortality (Table 5). When we extended the baseline models in sensitivity analyses by adding candidate mediator variables, only the simplified RALE score remained an independent predictor of hospital mortality (Table 6).

The present study revealed that nearly four-fifths of critically ill COVID-19 patients infected with the Delta variant died in the hospital (Tables 1, 2). This mortality rate is higher than the rates reported in other Vietnamese studies, namely, 52.2% (263/504) in Ho Chi Minh City (18) and 64.2% (97/151) in Binh Duong Province (39), as well as 54.6% (306/560) reported in an Indian study (40). These disparities may arise from variations in the inclusion criteria across studies. For example, our study only included critically ill COVID-19 patients who had CXRs taken upon hospital admission. Moreover, our hospital mortality rate greatly surpasses that reported in a Swedish study (38.2%; 190/498) (41). The variation observed may stem from differences in the patients, pathogens, and clinical ability to care for critically ill patients between low- to middle-income and high-income country settings (42–45). Additionally, the study center almost only admitted critically ill patients with COVID-19 (92.4%, 85/92; Supplementary Table 1) who encountered difficulties accessing treatment at lower-level hospitals, with a low rate of intubation (16.7%, 16/96; Supplementary Table 1) and mechanical ventilation (14.0%, 14/100; Supplementary Table 1) during transportation. The transfer of critically ill COVID-19 patients from local to central hospitals may exacerbate their clinical deterioration. In Vietnam, such transfers may occur without essential interventions like intubation, ventilation, PEEP, and other medical interventions (46–48). Although detailed data on patient transfer conditions are limited, previous surveys have identified multiple risk factors associated with increased patient transfers and suboptimal quality of pre-hospital care (49, 50). Therefore, the cohort of the present study is likely to be overestimated in terms of the hospital mortality rate.

During the early stages of the COVID-19 pandemic, ICU admission decisions for COVID-19 patients were primarily based on SpO_2_ levels and comorbidities (6). However, imaging modalities, particularly CXRs, have been suggested as valuable tools for assessing disease severity. CXRs can offer insights into the extent of lung involvement, (9, 10, 12–14, 51) which helps predict the prognosis of COVID-19 patients. Although data on simplified CXR-based scoring systems for mortality prediction remain limited, prior studies have demonstrated the potential of the simplified RALE score as an independent predictor of hospital mortality. For instance, a retrospective study in Pakistan identified the initial simplified RALE score (AOR: 1.278; 95% CI: 1.010–1.617) as an independent predictor of hospital mortality (13). Similarly, a Vietnamese retrospective study demonstrated a significant association between the simplified RALE score (OR: 1.10; 95% CI: 1.04–1.18) and a higher risk of hospital mortality (14). These findings align with the present study, which confirmed that a higher simplified RALE score was independently associated with an increased risk of hospital mortality, particularly when the score reaches ≥ 5.5 (Tables 5, 6). Furthermore, the present study also underscored the simplified RALE score’s good predictive ability in assessing hospital mortality risk (Table 3). Therefore, the simplified RALE score may serve as an effective tool for identifying critically ill COVID-19 patients at greatest risk of death.

The present study unveiled that the PaO_2_/FiO_2_ ratio and serum IL-6 level exhibited a poor discriminatory ability in predicting hospital mortality (Table 3). The reliability of these measures remains a topic of ongoing debate (52–61). For instance, a Chinese retrospective study demonstrated that the PaO_2_/FiO_2_ ratio (with an AUROC of 0.865 and a 95% CI of 0.748–0.941) demonstrated excellent discrimination for predicting hospital mortality among COVID-19 ICU patients (52). In contrast, an Italian prospective study reported a lower discriminatory ability of the PaO_2_/FiO_2_ ratio (AUROC: 0.688, 95% CI: 0.650–0.846), suggesting poor predictive performance (54). An earlier critique identified a caution problem with the PaO_2_/FiO_2_ ratio: while PaO_2_ accurately reflects a COVID-19 patient’s oxygenation, its reliability diminishes when expressed as a PaO_2_/FiO_2_ ratio (53). As a result, inconsistent data may arise regarding the predictive value of this ratio (52, 54). In terms of serum IL-6 level, the Chinese study found it had an excellent discriminatory ability to predict hospital mortality (AUROC: 0.900, 95% CI: 0.791–0.964) (52). However, a meta-analysis suggested that although serum IL-6 is a useful diagnostic marker for predicting severe disease, it does not appear to be associated with COVID-19 mortality (56). Interestingly, a Belgian retrospective study observed significant differences in serum IL-6 levels between survivors and non-survivors over time, with the maximum serum IL-6 value emerging as a predictor of ICU mortality in critically ill COVID-19 patients (57). Thus, the potential role of serum IL-6 as a prognostic biomarker in COVID-19 warrants further investigation.

The spectrum of COVID-19 in adults ranges from asymptomatic infection to mild respiratory tract symptoms to severe pneumonia with ARDS and multi-organ dysfunction. In patients with suspected COVID-19 pneumonia, key management steps involve assessing disease severity and identifying the appropriate level of care. Clinicians frequently use the CURB-65 severity score due to its straightforwardness (26). The present study found that the CURB-65 score exhibited good discrimination in predicting hospital mortality (Table 3), consistent with the finding from a comprehensive analysis of 22 predictive models applied to 411 hospitalized adults with COVID-19, in which the CURB-65 score also showed good discrimination for 30-day mortality (AUROC: 0.75, 95% CI: 0.7–0.8) (62). Although several predictive models have been proposed, none have emerged as clearly superior or consistently accurate in forecasting deterioration or mortality in critically ill COVID-19 patients (42, 43, 62–66). Although the APACHE IV score is the most up-to-date version, some centers still use older versions, including the APACHE II score. The present study showed that the APACHE II score had poor discrimination in predicting hospital mortality in critically ill COVID-19 patients (Table 3), which aligns with a Belgian retrospective study that reported similarly poor discrimination using APACHE II (AUROC: 0.633) (66). These findings are supported by previous studies, which revealed that the APACHE II score had a good prognostic value in acutely ill or surgical patients (25, 67) but has limitations in distinguishing sterile from infected necrotizing pancreatitis and in predicting the severity of acute pancreatitis within 24 h (68). In contrast, the SOFA score in this study demonstrated good discriminatory performance in predicting hospital mortality (Table 3). However, this contrasts with findings from a large American cohort of COVID-19 patients, excluding those on mechanical ventilation within the first 24 h or with a do-not-resuscitate (DNR) designation at admission, where the SOFA score had poor discriminatory ability (AUROC: 0.66, 95% CI: 0.65–0.67) (64). This variation might be because the illness severity of our patients was worse than those of the large American cohort [mean SOFA score: 2.77 (SD: 1.91)] (64). These findings are supported by an Iranian prospective study, which reported that the mean of daily SOFA scores during ICU admission (AUROC: 0.895) had excellent discrimination in predicting hospital mortality (69).

As previously noted, the predictive value of the test results and models discussed for assessing critically ill COVID-19 patients remains uncertain. The optimal use of these diagnostic markers is also unclear. However, the present study shows that the simplified RALE score, SOFA score, and CURB-65 score demonstrated good discrimination in predicting hospital mortality (Table 3), with no differences between their AUROC curves (Supplementary Table 6). Although the simplified RALE score showed only modest coefficients, it correlated with both SOFA and CURB-65 scores (Table 4). In baseline multivariable models that controlled for the same set of baseline confounders, we found that only higher simplified RALE and CURB-65 scores were independently associated with increased risk of hospital mortality (Table 5). When we included mediator variables in these models, only the simplified RALE score remained an independent predictor of mortality. The key finding is that the simplified RALE score consistently predicted hospital mortality across all analyses, even after adjustment. Additionally, we observed that survivors with radiological involvement in all four quadrants differed significantly from non-survivors (Table 2), suggesting that the simplified RALE score may capture critical lung injury not captured by clinical scoring systems. This reinforces the added prognostic value of an imaging-based approach in high-severity cases. Our findings support previous studies showing that CXR-based scoring systems can reliably assess disease severity and predict mortality by quantifying the extent of lung involvement in critically ill COVID-19 patients (9, 10, 12–14). Importantly, radiography is widely available, rapid, and cost-effective, making it particularly valuable in resource-limited settings where other diagnostic methods may not be feasible. Therefore, the simplified RALE score represents a practical and efficient tool for quantifying COVID-19 pneumonia severity and guiding clinical management.

### 4.1 Limitations

This retrospective observational study has several important limitations. We present them in sequence, from study design and population characteristics to measurement, analysis, and applicability. For each, we also note strengths relevant to scientific and clinical assessment, particularly in high-burden, resource-limited settings during the COVID-19 pandemic.

First, the sample size was modest (*n* = 105), and the outcome was imbalanced (22 survivors [21%] vs. 83 non-survivors [79%]), limiting statistical power and increasing the risk of overfitting in multivariable logistic regression. Some AORs had wide 95% confidence intervals (e.g., the simplified RALE AOR of 2.170; 95% CI, 1.353–3.482). The low event count prevented robust subgroup or higher-order interaction analyses. The relatively high event rate permitted identification of an independent association between the simplified RALE score and hospital mortality after adjusting for baseline confounders (transfer status, demographics, comorbidities, vital signs, labs, and gas-exchange data). Complete-case sensitivity analyses (*n* = 87 for the main model, see Supplementary Table 32) and calibration checks [Hosmer–Lemeshow χ^2^(8) = 12.222, *p* = 0.142; pseudo-R^2^ = 0.532] yielded consistent results (shown at the bottom of Supplementary Table 8), providing hypothesis-generating evidence from a Delta-variant surge in Southeast Asia, where data on imaging-based prognostication in critically ill patients were previously limited.

Second, the single-center, retrospective design at a temporary 360-bed field hospital (Field Hospital Number 16, affiliated with Bach Mai Hospital) during the peak Delta-variant outbreak in Ho Chi Minh City restricts external generalizability. Patients were exclusively critically ill adults transferred from lower-level facilities under extreme healthcare-system strain, producing an unusually high hospital mortality rate (79%), near-universal bilateral lung opacities (99%), and a median simplified RALE score of 8.0 (IQR 6.0–8.0). This introduces selection bias, as chest X-rays were obtained only when clinically indicated in severely ill patients, potentially conferring a “home-field advantage” to an imaging-based score relative to non-radiological tools such as SOFA or CURB-65. Nevertheless, this real-world context represents a major strength: the study captures authentic, high-stakes conditions in a low- and middle-income country during healthcare overload, where simple, bedside prognostic tools are most urgently needed. The findings, therefore, provide contextually relevant, actionable insights that complement data from high-resource settings and directly inform risk stratification when advanced diagnostics are unavailable.

Third, although standardized, radiographic assessment has inherent limitations in reproducibility and temporal consistency. Bedside anteroposterior CXRs were scored using a pragmatic two-step process (initial review by a third-year radiology resident followed by independent validation by a thoracic radiologist with 10 years’ experience; both blinded to clinical data and outcomes), based on the simplified RALE system (0–8 scale; see Supplementary Figures 1, 2). However, inter-rater reliability was not formally quantified in this dataset, and only admission (not serial) images were analyzed. Consequently, the score reflects a single time-point snapshot and may not capture dynamic disease evolution or subtle heterogeneity in radiographic patterns (e.g., consolidation versus ground-glass opacities). At the same time, the use of portable bedside equipment and a rapid, consensus-based scoring protocol closely mirrors real-world ICU practice in resource-limited environments, thereby enhancing the tool’s feasibility and immediate clinical applicability. The resulting excellent discrimination (AUROC 0.747, 95% CI 0.617–0.877) in this challenging cohort underscores the robustness of the simplified RALE score as a low-cost, rapidly obtainable prognostic marker.

Fourth, while statistical analysis improved after peer review, primary models included only baseline confounders, mediator-adjusted models moved to sensitivity analyses (Supplementary Tables 20–31), multicollinearity was checked (all VIF < 5, shown at the bottom of Supplementary Tables 8–31), calibration was assessed (Supplementary Figures 3, 4 and Supplementary Tables 8–31), and sensitivity analyses included complete-case analyses, which supported the primary findings (Supplementary Tables 32–55), the study lacks internal validation (bootstrapping or cross-validation) and external validation. This may cause optimism bias in performance metrics. Still, transparent reporting of both baseline-only and full models, along with AUROC comparisons with SOFA and CURB-65 (*p* > 0.05), boosts confidence in the findings and provides a sound basis for future validation studies.

Finally, the exclusive focus on laboratory-confirmed Delta-variant cases in a pre-vaccination-peak, resource-limited pandemic setting limits applicability to subsequent variants, vaccinated populations, or settings with routine CT availability. This specificity, however, constitutes a timely and unique scientific contribution: the study is among the first to rigorously validate the simplified RALE score in critically ill patients with severe Delta-variant pneumonia in Vietnam, generating actionable data at a time when such surges overwhelmed healthcare systems globally.

These limitations in design, sampling, measurement, and analysis require caution in interpretation but are balanced by key strengths: real-world data under extreme conditions, transparent methods, and validation of a simple prognostic tool with independent predictive value (AOR 1.934 after full adjustment). Future research should focus on larger, prospective, multicentre validation across different regions, severities, variants, and resources. Serial imaging, formal inter-rater reliability assessments, external cohorts, and advanced techniques (e.g., machine-learning-derived radiographic scores) can further enhance the score’s utility and clarify its added value in critical care. These efforts will directly build on this work to support broader clinical use.

## 5 Conclusion

This study investigated a highly selected cohort of critically ill COVID-19 patients with the Delta variant, characterized by high simplified RALE scores and elevated hospital mortality, at an Intensive Care Center for the Treatment of Critically Ill Patients with COVID-19 in Ho Chi Minh City, Vietnam, and found that the simplified RALE score performed comparably to SOFA and CURB-65 in predicting hospital mortality. Importantly, the simplified RALE score remained an independent predictor of mortality in multivariable analyses. While these findings are promising, larger and more diverse studies are required to validate them and support broader clinical application.

## Data Availability

The original contributions presented in this study are included in this article/Supplementary material, further inquiries can be directed to the corresponding author.
