# Supplementary material for: Predictive validity of the simplified Radiographic Assessment of Lung Edema score for mortality in critically ill patients with COVID-19
Source: Front Med (Lausanne). 2026 May 20;13:1819896. doi: 10.3389/fmed.2026.1819896 (PMC13230042; doi:10.3389/fmed.2026.1819896)
Supplement: Supplementary file 1 [file Data_Sheet_1.pdf]

SUPPLEMENTARY RESULTS

Table of contents

Supplementary Figure 1. Consolidation and Density Scoring in the Radiographic Assessment of Lung Oedema (RALE) Score

Supplementary Figure 2. The Diagram and Radiograph Illustrate the Severity Classification of COVID-19 Based on Lung Involvement, as Observed in A Frontal Chest X-Ray

Supplementary Table 1. Prehospital Care for Critically Ill COVID-19 Patients with Delta Variant, According to Hospital Survivability

Supplementary Table 2. The Laboratory Characteristics of Critically Ill COVID-19 Patients with Delta Variant Upon Admission, According to Hospital Survivability

Supplementary Table 3. The Gas Exchange in Critically Ill COVID-19 Patients with Delta Variant upon Admission, According to Hospital Survivability

Supplementary Table 4. The First-Day Respiratory Support and Adjunctive Therapies during the Hospital Stay for Critically Ill COVID-19 Patients with Delta Variant, According to Hospital Survivability

Supplementary Table 5. Complications in Critically Ill COVID-19 Patients with Delta Variant, According to Hospital Survivability

Supplementary Table 6. Pairwise Comparisons of AUROC of the Simplified RALE and the Severity Scoring Systems upon Admission for Predicting Hospital Mortality

Supplementary Table 7. Spearman's Correlation between the Simplified RALE Score and the Severity Scoring Systems upon Admission

Supplementary Table 8. Multivariable Regression Analysis of Simplified RALE Score and Hospital Mortality in Critically Ill COVID-19 Patients with Delta Variant upon Admission, Adjusted for Baseline Confounders

Supplementary Table 9. Multivariable Regression Analysis of Simplified RALE Score ≥Cut-off Value and Hospital Mortality in Critically Ill COVID-19 Patients with Delta Variant upon Admission, Adjusted for Baseline Confounders

Supplementary Table 10. Multivariable Regression Analysis of SOFA Score and Hospital Mortality in Critically Ill COVID-19 Patients with Delta Variant upon Admission, Adjusted for Baseline Confounders

Supplementary Table 11. Multivariable Regression Analysis of SOFA Score ≥Cut-off Value and Hospital Mortality in Critically Ill COVID-19 Patients with Delta Variant upon Admission, Adjusted for Baseline Confounders

Supplementary Table 12. Multivariable Regression Analysis of APACHE II Score and Hospital Mortality in Critically Ill COVID-19 Patients with Delta Variant upon Admission, Adjusted for Baseline Confounders

Supplementary Table 13. Multivariable Regression Analysis of APACHE II Score ≥Cut-off Value and Hospital Mortality in Critically Ill COVID-19 Patients with Delta Variant upon Admission, Adjusted for Baseline Confounders

Supplementary Table 14. Multivariable Regression Analysis of CURB-65 Score and Hospital Mortality in Critically Ill COVID-19 Patients with Delta Variant upon Admission, Adjusted for Baseline Confounders

Supplementary Table 15. Multivariable Regression Analysis of CURB-65 Score ≥Cut-off Value and Hospital Mortality in Critically Ill COVID-19 Patients with Delta Variant upon Admission, Adjusted for Baseline Confounders

Supplementary Table 16. Multivariable Regression Analysis of First-day PaO2/FiO2 Ratio and Hospital Mortality in Critically Ill COVID-19 Patients with Delta Variant upon Admission, Adjusted for Baseline Confounders

Supplementary Table 17. Multivariable Regression Analysis of First-day PaO2/FiO2 Ratio ≥Cut-off Value and Hospital Mortality in Critically Ill COVID-19 Patients with Delta Variant upon Admission, Adjusted for Baseline Confounders

Supplementary Table 18. Multivariable Regression Analysis of IL-6 Level and Hospital Mortality in Critically Ill COVID-19 Patients with Delta Variant upon Admission, Adjusted for Baseline Confounders

Supplementary Table 19. Multivariable Regression Analysis of IL-6 Level ≥Cut-off Value and Hospital Mortality in Critically Ill COVID-19 Patients with Delta Variant upon Admission, Adjusted for Baseline Confounders

Supplementary Table 20. Multivariable Regression Analysis of Simplified RALE Score and Hospital Mortality in Critically Ill COVID-19 Patients with Delta Variant upon Admission, Adjusted for Baseline and Mediator Confounders

Supplementary Table 21. Multivariable Regression Analysis of Simplified RALE Score ≥Cut-off Value and Hospital Mortality in Critically Ill COVID-19 Patients with Delta Variant upon Admission, Adjusted for Baseline and Mediator Confounders

Supplementary Table 22. Multivariable Regression Analysis of SOFA Score and Hospital Mortality in Critically Ill COVID-19 Patients with Delta Variant upon Admission, Adjusted for Baseline and Mediator Confounders

Supplementary Table 23. Multivariable Regression Analysis of SOFA Score ≥Cut-off Value and Hospital Mortality in Critically Ill COVID-19 Patients with Delta Variant upon Admission, Adjusted for Baseline and Mediator Confounders

Supplementary Table 24. Multivariable Regression Analysis of APACHE II Score and Hospital Mortality in Critically Ill COVID-19 Patients with Delta Variant upon Admission, Adjusted for Baseline and Mediator Confounders

Supplementary Table 25. Multivariable Regression Analysis of APACHE II Score ≥Cut-off Value and Hospital Mortality in Critically Ill COVID-19 Patients with Delta Variant upon Admission, Adjusted for Baseline and Mediator Confounders

Supplementary Table 26. Multivariable Regression Analysis of CURB-65 Score and Hospital Mortality in Critically Ill COVID-19 Patients with Delta Variant upon Admission, Adjusted for Baseline and Mediator Confounders

Supplementary Table 27. Multivariable Regression Analysis of CURB-65 Score ≥Cut-off Value and Hospital Mortality in Critically Ill COVID-19 Patients with Delta Variant upon Admission, Adjusted for Baseline and Mediator Confounders

Supplementary Table 28. Multivariable Regression Analysis of First-day PaO2/FiO2 Ratio and Hospital Mortality in Critically Ill COVID-19 Patients with Delta Variant upon Admission, Adjusted for Baseline and Mediator Confounders

Supplementary Table 29. Multivariable Regression Analysis of First-day PaO2/FiO2 Ratio ≥Cut-off Value and Hospital Mortality in Critically Ill COVID-19 Patients with Delta Variant upon Admission, Adjusted for Baseline and Mediator Confounders

Supplementary Table 30. Multivariable Regression Analysis of IL-6 Level and Hospital Mortality in Critically Ill COVID-19 Patients with Delta Variant upon Admission, Adjusted for Baseline and Mediator Confounders

Supplementary Table 31. Multivariable Regression Analysis of IL-6 Level ≥Cut-off Value and Hospital Mortality in Critically Ill COVID-19 Patients with Delta Variant upon Admission, Adjusted for Baseline and Mediator Confounders

Supplementary Table 32. Complete-Case Sensitivity Analysis of the Association of Simplified RALE Score with Hospital Mortality in Critically Ill COVID-19 Patients with Delta Variant upon Admission, Adjusted for Baseline Confounders

Supplementary Table 33. Complete-Case Sensitivity Analysis of the Association of Simplified RALE Score Dichotomized at Cut-off with Hospital Mortality in Critically Ill COVID-19 Patients with Delta Variant upon Admission, Adjusted for Baseline Confounders

Supplementary Table 34. Complete-Case Sensitivity Analysis of the Association of SOFA Score with Hospital Mortality in Critically Ill COVID-19 Patients with Delta Variant upon Admission, Adjusted for Baseline Confounders

Supplementary Table 35. Complete-Case Sensitivity Analysis of the Association of SOFA Score Dichotomized at Cut-off with Hospital Mortality in Critically Ill COVID-19 Patients with Delta Variant upon Admission, Adjusted for Baseline Confounders

Supplementary Table 36. Complete-Case Sensitivity Analysis of the Association of APACHE II Score with Hospital Mortality in Critically Ill COVID-19 Patients with Delta Variant upon Admission, Adjusted for Baseline Confounders

Supplementary Table 37. Complete-Case Sensitivity Analysis of the Association of APACHE II Score Dichotomized at Cut-off with Hospital Mortality in Critically Ill COVID-19 Patients with Delta Variant upon Admission, Adjusted for Baseline Confounders

Supplementary Table 38. Complete-Case Sensitivity Analysis of the Association of CURB-65 Score with Hospital Mortality in Critically Ill COVID-19 Patients with Delta Variant upon Admission, Adjusted for Baseline Confounders

Supplementary Table 39. Complete-Case Sensitivity Analysis of the Association of CURB-65 Score Dichotomized at Cut-off with Hospital Mortality in Critically Ill COVID-19 Patients with Delta Variant upon Admission, Adjusted for Baseline Confounders

Supplementary Table 40. Complete-Case Sensitivity Analysis of the Association of First-day PaO_2/FiO_2 Ratio with Hospital Mortality in Critically Ill COVID-19 Patients with Delta Variant upon Admission, Adjusted for Baseline Confounders

Supplementary Table 41. Complete-Case Sensitivity Analysis of the Association of First-day PaO_2/FiO_2 Ratio Dichotomized at Cut-off with Hospital Mortality in Critically Ill COVID-19 Patients with Delta Variant upon Admission, Adjusted for Baseline Confounders

Supplementary Table 42. Complete-Case Sensitivity Analysis of the Association of IL-6 Level with Hospital Mortality in Critically Ill COVID-19 Patients with Delta Variant upon Admission, Adjusted for Baseline Confounders

Supplementary Table 43. Complete-Case Sensitivity Analysis of the Association of IL-6 Level Dichotomized at Cut-off with Hospital Mortality in Critically Ill COVID-19 Patients with Delta Variant upon Admission, Adjusted for Baseline Confounders

Supplementary Table 44. Complete-Case Sensitivity Analysis of the Association of Simplified RALE Score with Hospital Mortality in Critically Ill COVID-19 Patients with Delta Variant upon Admission, Adjusted for Baseline and Mediator Confounders

Supplementary Table 45. Complete-Case Sensitivity Analysis of the Association of Simplified RALE Score Dichotomized at Cut-off with Hospital Mortality in Critically Ill COVID-19 Patients with Delta Variant upon Admission, Adjusted for Baseline and Mediator Confounders

Supplementary Table 46. Complete-Case Sensitivity Analysis of the Association of SOFA Score with Hospital Mortality in Critically Ill COVID-19 Patients with Delta Variant upon Admission, Adjusted for Baseline and Mediator Confounders

Supplementary Table 47. Complete-Case Sensitivity Analysis of the Association of SOFA Score Dichotomized at Cut-off with Hospital Mortality in Critically Ill COVID-19 Patients with Delta Variant upon Admission, Adjusted for Baseline and Mediator Confounders

Supplementary Table 48. Complete-Case Sensitivity Analysis of the Association of APACHE II Score with Hospital Mortality in Critically Ill COVID-19 Patients with Delta Variant upon Admission, Adjusted for Baseline and Mediator Confounders

Supplementary Table 49. Complete-Case Sensitivity Analysis of the Association of APACHE II Score Dichotomized at Cut-off with Hospital Mortality in Critically Ill COVID-19 Patients with Delta Variant upon Admission, Adjusted for Baseline and Mediator Confounders

Supplementary Table 50. Complete-Case Sensitivity Analysis of the Association of CURB-65 Score with Hospital Mortality in Critically Ill COVID-19 Patients with Delta Variant upon Admission, Adjusted for Baseline and Mediator Confounders

Supplementary Table 51. Complete-Case Sensitivity Analysis of the Association of CURB-65 Score Dichotomized at Cut-off with Hospital Mortality in Critically Ill COVID-19 Patients with Delta Variant upon Admission, Adjusted for Baseline and Mediator Confounders

Supplementary Table 52. Complete-Case Sensitivity Analysis of the Association of First-day PaO_2/FiO_2 Ratio with Hospital Mortality in Critically Ill COVID-19 Patients with Delta Variant upon Admission, Adjusted for Baseline and Mediator Confounders

Supplementary Table 53. Complete-Case Sensitivity Analysis of the Association of First-day PaO_2/FiO_2 Ratio Dichotomized at Cut-off with Hospital Mortality in Critically Ill COVID-19 Patients with Delta Variant upon Admission, Adjusted for Baseline and Mediator Confounders

Supplementary Table 54. Complete-Case Sensitivity Analysis of the Association of IL-6 Level with Hospital Mortality in Critically Ill COVID-19 Patients with Delta Variant upon Admission, Adjusted for Baseline and Mediator Confounders

Supplementary Table 55. Complete-Case Sensitivity Analysis of the Association of IL-6 Level Dichotomized at Cut-off with Hospital Mortality in Critically Ill COVID-19 Patients with Delta Variant upon Admission, Adjusted for Baseline and Mediator Confounders

Supplementary Figure 3. Calibration plot for the multivariable logistic regression model in Supplementary Table 8

Supplementary Figure 4. Calibration plot for the multivariable logistic regression model in Supplementary Table 20

CONSOLIDATION[a]	
**Consolidation Score**	**Extent of alveolar opacities**
0	None
1	< 25%
2	25 – 50%
3	50 – 75%
4	> 75%
**DENSITY[b]**	
1	Hazy
2	Moderate
3	Dense
**FINAL RALE SCORE[c]**	
**Right Lung**	**Left Lung**
Upper Quadrant<br>Consolidation Score x Density Score = Upper Quadrant (Q1) Score	Upper Quadrant<br>Consolidation Score x Density Score = Upper Quadrant (Q3) Score
Lower Quadrant<br>Consolidation Score x Density Score = Lower Quadrant (Q2) Score	Lower Quadrant<br>Consolidation Score x Density Score = Lower Quadrant (Q4) Score
**Total RALE Score = Q1 + Q2 + Q3 + Q4**	

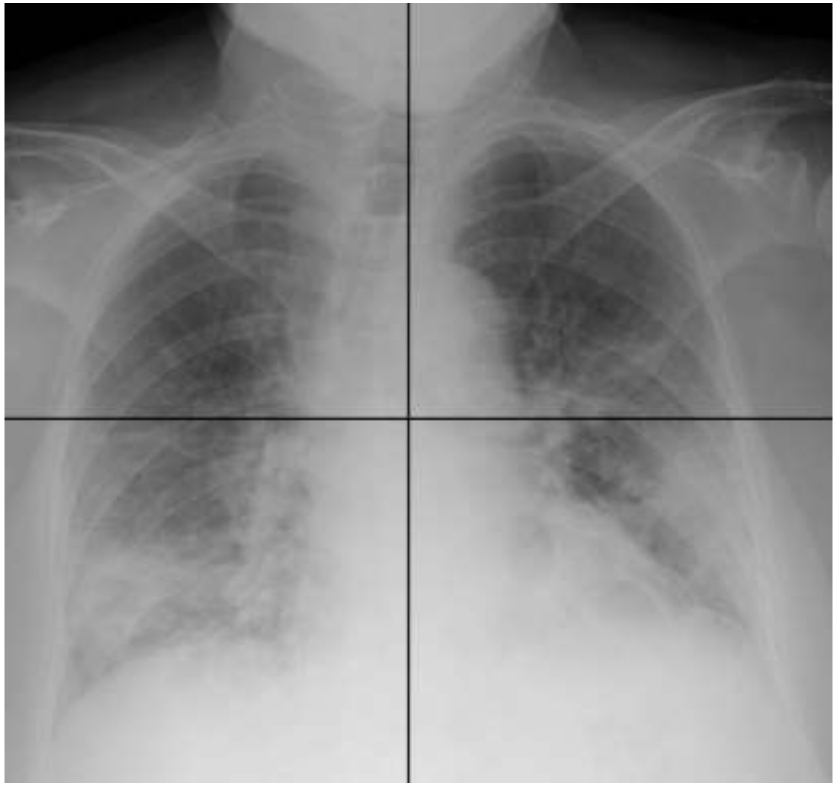

Calculation of the RALE Score

**Score**	**Q1**	**Q2**	**Q3**	**Q4**	**Total**
Consolidation	1	4	2	4	
Density	2	3	2	3	
Quadrant Score	1x2=2	4x3=12	2x2=4	4x3=12	**30**

[a] Consolidation is scored for each quadrant; [b] Density is scored for each quadrant that has a consolidation score of ≥1; [c] If the quadrant consolidation score is 0 then the quadrant score is 0; Abbreviations: **Q1**, upper quadrant of the right lung; **Q2**, lower quadrant of the right lung; **Q3**, upper quadrant of the left lung; **Q4**, lower quadrant of the left lung; **RALE Score**, Radiographic Assessment of Lung Oedema Score. *It's important to note that, for scientific and non-commercial purposes, the radiograph has been reproduced without official authorization from the Bach Mai Hospital, and all rights remain with this organization*.

Supplementary Figure 1. Consolidation and Density Scoring in the Radiographic Assessment of Lung Oedema (RALE) Score

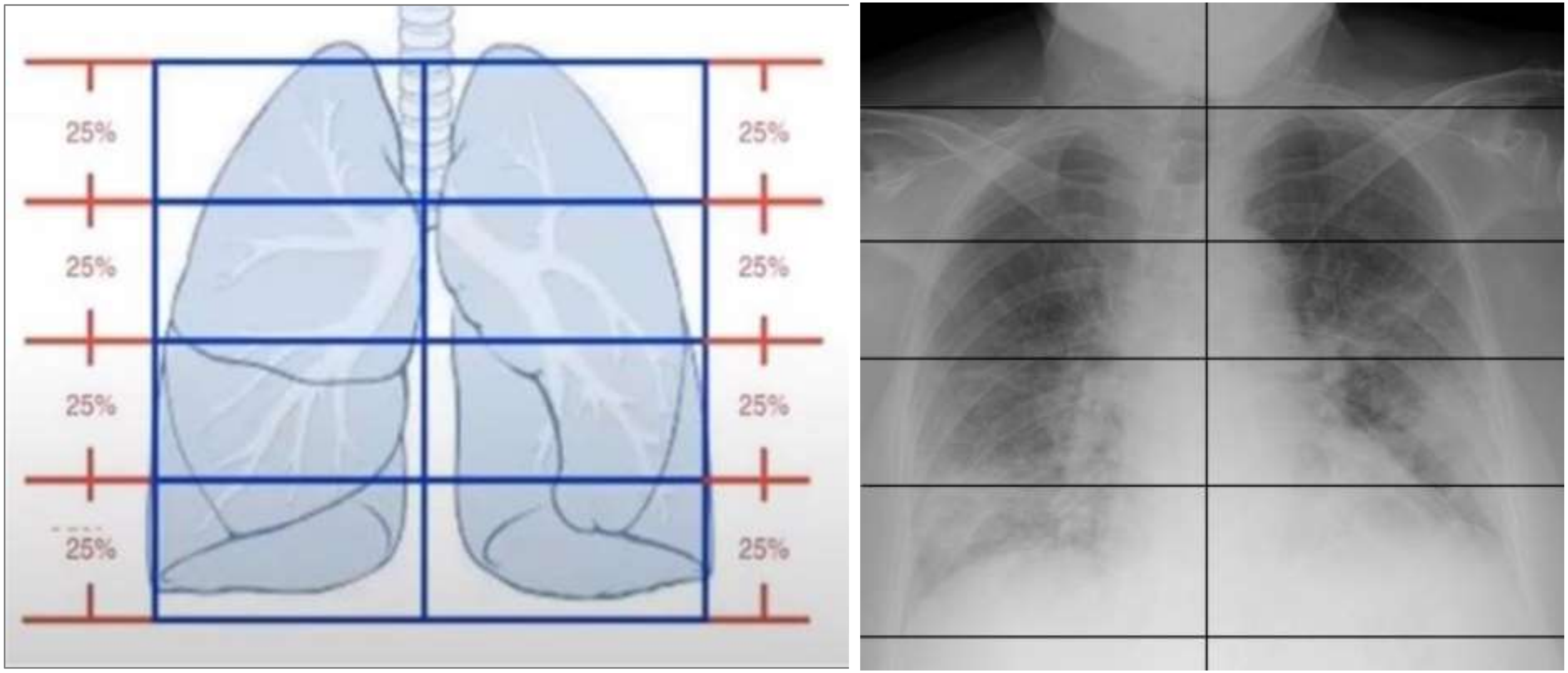

Supplementary Figure 2. The Diagram and Radiograph Illustrate the Severity Classification of COVID-19 Based on Lung Involvement, as Observed in A Frontal Chest X-Ray. In the diagram, the simplified Radiographic Assessment of Lung Edema (simplified RALE) scoring system assigns a score to each lung, ranging from 0 to 4. This score reflects the presence of specific abnormalities. The system divides each lung into four zones, and abnormalities such as consolidation, ground-glass opacification, and reticular interstitial thickening receive a score of 1 in each zone. The overall severity score is calculated by summing up the points from both lungs, with a maximum possible score of 8. In the radiograph,

six lung zones exhibit abnormalities, resulting in a simplified RALE Score of 6. *It's important to note that, for scientific and non-commercial purposes, the diagram and radiograph have been reproduced without official authorization from the Vietnam Ministry of Health and Bach Mai Hospital, respectively, and all rights remain with these organizations.*

Supplementary Table 1. Inter-hospital Care for Critically Ill COVID-19 Patients with Delta Variant, According to Hospital Survivability

Variables	All cases n=105	Survived n=22	Died n=83	P value[a]
Prior hospitalization, no. (%), n=92	85 (92.4)	14 (73.7)	71 (97.3)	0.004
Prior hospitalization				
Duration of stay (day), median (IQR), n=80	3.5 (1.0–7.0)	3 (1.0–5.3)	4 (1.0–9.3)	0.293
MV applied, no. (%), n=82	16 (19.5)	2 (15.4)	14 (20.3)	>0.999
Duration of MV (day), median (IQR), n=16	1.0 (1.0–7.8)	3(2)	1 (1.0–9.3)	0.382
The patient was brought in by, n=87				0.017
EMS, no. (%)	32 (36.8)	5 (29.4)	27 (38.6)	
Interhospital ambulances, no. (%)	52 (59.8)	9 (52.9)	43 (61.4)	
Private ambulances, no. (%)	0 (0.0)	0 (0.0)	0 (0.0)	
Public or own/private transport, no. (%)	2 (2.3)	2 (11.8)	0 (0.0)	
Unknown, no. (%)	1 (1.1)	1 (5.9)	0 (0.0)	
Inter-hospital care provider, n=75				0.007
Non-EMT, no. (%)	19 (25.3)	3 (27.3)	16 (25.0)	

EMT, no. (%)	18 (24.0)	2 (18.2)	16 (25.0)	
Doctors, no. (%)	35 (46.7)	3 (27.3)	32 (50.0)	
Nurses, no. (%)	2 (2.7)	2 918.2)	0 (0.0)	
Unknown, no. (%)	1 (1.3)	1 (9.1)	0 (0.0)	
Airway during inter-hospital transfers, n=96				
None, no. (%)	79 (82.3)	18 (85.7)	61 (81.3)	0.757
Oropharyngeal airway, no. (%)	1 (1.0)	0 (0.0)	1 (1.3)	>0.999
Supraglottic airway, no. (%)	0 (0.0)	0 (0.0)	0 (0.0)	NA
Endotracheal tube, no. (%)	16 (16.7)	2 (9.5)	14 (18.7)	0.510
Others, no. (%)	0 (0.0)	0 (0.0)	0 (0.0)	NA
Oxygen during inter-hospital transfers, n=100				
None, no. (%)	24 (24.0)	7 (33.3)	17 (21.5)	0.260
Nasal cannula, no. (%)	4 (4.0)	3 (14.3)	1 (1.3)	0.028
Venturi mask, no. (%)	1 (1.0)	0 (0.0)	1 (1.3)	>0.999
Facial mask, no. (%)	4 (4.0)	1 (4.8)	3 (3.8)	>0.999
Bag valve mask, no. (%)	39 (39.0)	8 (38.1)	31 (39.2)	0.924
High-flow nasal cannula, no. (%)	15 (15.0)	0 (0.0)	15 (19.0)	0.036
Non-invasive ventilator, no. (%)	5 (5.0)	0 (0.0)	5 (6.3)	0.581
Mechanical ventilator, no. (%)	14 (14.0)	2 (9.5)	12 (15.2)	0.728
Others, no. (%)	0 (0.0)	0 (0.0)	0 (0.0)	NA

[a] The comparison between patients who survived and those who died in the hospital.

Abbreviations: **EMS**, emergency medical services; **EMT**, emergency medical technician; **IQR**, interquartile range; **MV**, mechanical ventilation; **NA**, not available; **no.**, number of patients; **SD**, standard deviation.

Supplementary Table 2. The Laboratory Characteristics of Critically Ill COVID-19 Patients with Delta Variant Upon Admission, According to Hospital Survivability

Variables	All cases n=105	Survived n=22	Died n=83	P value[a]
WBC (x10^9/L), mean (SD)	12.94 (5.73)	11.94 (4.78)	13.21 (5.95)	0.358
Haemoglobin (g/L), median (IQR)	128.0 (114.5–138.0)	122.50 (111.8–133.3)	129.00 (115.0–142.0)	0.317
Platelet count (x10^9/L), median (IQR)	229.0 (174.5–323.0)	226.50 (183.3–285.0)	238.00 (167.0–329.0)	0.937
CRP (mg/L), median (IQR), n=90	10.5 (4.8–14.0)	7.48 (2.8–13.5)	10.44 (4.9–14.1)	0.295
Procalcitonin (ng/mL), median (IQR), n=35	0.4 (0.1–1.1)	0.2 (0.1–0.4)	0.5 (0.1–2.0)	0.131
Troponin T (ng/L), median (IQR), n=66	24.1 (9.5–76. 5)	9.6 (5.25–36.6)	21.2 (11.9–124.2)	0.023
ProBNP (pg/mL), median (IQR), n=52	102.2 (46.7–235.6)	40.3 (28.4–49.9)	145.4 (61.6–240.1)	0.002
Total bilirubin (µmol/L), median (IQR), n=53	11.0 (8.1–17.8)	13.4 (8.3–20.5)	10.5 (8.0–16.3)	0.411
AST (U/L), median (IQR)	38.0 (25.5–59.5)	41.0 (25.0–70.8)	37.0 (25.0–60.0)	0.789
ALT (U/L), median (IQR)	54.0 (35.0–83.0)	56.5 (34.8–81.3)	53.0 (35.0–85.0)	0.878
Glucose (mmol/L), median (IQR), n=101	10.0 (7.5–14.4)	9.50 (5.8–13.8)	10.2 (7.5–14.6)	0.473
Ure (mmol/L), median (IQR), n=104	6.8 (5.0–9.7)	5.35 (4.4–6.9)	7.20 (5.2–10.0)	0.005
Creatinine (µmol/L), median (IQR), n=104	81.0 (68.0–91.8)	70.50 (63.8–86.0)	82.50 (68.8–96.3)	0.040

INR, median (IQR)	1.1 (1.0–1.3)	1.11 (1.0–1.3)	1.12 (1.0–1.3)	0.576

[a] The comparison between patients who survived and those who died in the hospital.
Abbreviations: **ALT**, alanine transaminase; **AST**, aspartate transaminase; **CRP**, C-reactive protein; **INR**, international normalized ratio; **IQR**, interquartile range; **ProBNP**, N-terminal pro-B-type natriuretic peptide; **SD**, standard deviation; **WBC**, white blood cell.

Supplementary Table 3. The Gas Exchange in Critically Ill COVID-19 Patients with Delta Variant upon Admission, According to Hospital Survivability

Variables	All cases n=105	Survived n=22	Died n=83	P value[a]
**The 1st-day gas exchange**				
pH, median (IQR), n=63	7.43 (7.3–7.5)	7.50 (7.4–7.5)	7.40 (7.3–7.5)	0.006
PaO_2 (mmHg), median (IQR),n=63	70.00 (58.0–83.0)	78.00 (67.0–115.0)	67.00 (58.0–81.8)	0.135
$PaCO_2$ (mmHg), median (IQR), n=63	39.00 (34.0–48.0)	38.00 (30.0–41.0)	38.50 (34.0–51.8)	0.224
FiO_2 (%),median (IQR), n=74	97.5 (70.0–100.0)	80.00 (50.0–100.0)	100.00 (70.0–100.0)	0.092
PaO_2/FiO_2 ratio, median (IQR), n=62	85.0 (65.3–119.3)	120.00 (83.0–178.0)	81.00 (63.0–101.0)	0.045
SpO_2 (%),median (IQR),n=87	94.00 (90.0–95.0)	95.00 (94.0–97.0)	93.00 (90.0–95.0)	0.042

[a] The comparison between patients who survived and those who died in the hospital.
Abbreviations: **IQR**, interquartile range; **$PaCO_2$**, arterial carbon dioxide partial pressure; **PaO_2**, arterial oxygen partial pressure; **PaO_2/FiO_2**, arterial oxygen partial pressure to inspired oxygen fraction ratio; **pH**, blood potential hydrogen; **SD**, standard deviation; **SpO_2**, peripheral oxygen saturation.

Supplementary Table 4. The First-Day Respiratory Support and Adjunctive Therapies during the Hospital Stay for Critically Ill COVID-19 Patients with Delta Variant, According to Hospital Survivability

Variables	All cases n=105	Survived n=22	Died n=83	P value[a]
**1st-day respiratory support**				
Oxygen supplements, no. (%), n=104	101 (97.1)	21 (95.5)	80 (97.6)	0.514
Nasal cannula, no. (%), n=101	2 (2.0)	2 (10.0)	0 (0.0)	0.038
Venturi mask, no. (%), n=100	1 (1.0)	0 (0.0)	1 (1.2)	>0.999
Facial mask, no. (%), n=100	4 (4.0)	1 (5.0)	3 (3.8)	>0.999
Bag valve mask, no. (%), n-102	39 (38.2)	12 (57.1)	27 (33.3)	0.045
High-flow nasal cannula, no. (%), n=101	42 (41.6)	5 (25.0)	37 (45.7)	0.093
Mechanical ventilation, no. (%), n=102	35 (34.3)	4 (20.0)	31 (37.8)	0.133
**Adjunctive therapies during the hospital stay**				
Prone positioning, no. (%), n=91	24 (26.4)	5 (25.0)	19 (26.8)	0.875
Recruitment manoeuvres, no. (%), n=87	15 (17.2)	0.0 (0.0)	15 (21.7)	0.034
ECMO, no. (%), n=104	2 (1.9)	0.0 (0.0)	2 (2.4)	>0.999
Antiviral drugs, no. (%), n=104	66 (63.5)	17 (77.3)	49 (59.8)	0.130
Antibiotics, no. (%)	103 (98.1)	20 (90.9)	83 (100.0)	0.042
Corticosteroids, no. (%), n=104	95 (91.3)	22 (100.0)	73 (89.0)	0.199
Heparin, no. (%), n=104	100 (96.2)	22 (100.0)	78 (95.1)	0.576

Antiplatelet drugs, no. (%)	4 (3.8)	0.0 (0.0)	4 (4.8)	0.577
NOACs, no. (%), n=104	4 (3.8)	1 (4.8)	3 (3.6)	>0.999
Recombinant humanized anti-IL-6 receptor monoclonal antibody, no. (%)	4 (3.8)	1 (4.5)	3 (3.6)	>0.999
Continuous sedation, no. (%)	83 (79.0)	5 (22.7)	78 (94.0)	<0.001
NMBAs, no. (%), n=104	70 (67.3)	6 (28.6)	64 (77.1)	<0.001
Renal replacement therapy, no. (%)	19 (18.1)	1 (4.5)	18 (21.7)	0.070
Cytokine adsorption therapy, no. (%)	30 (28.6)	6 (27.3)	24 (28.9)	0.879
Tracheostomy, no. (%)	7 (6.7)	2 (9.1)	5 (6.0)	0.635
Inhaled vasodilators, no. (%)	1 (1.0)	0.0 (0.0)	1 (1.2)	>0.999

[a] The comparison between patients who survived and those who died in the hospital.

Abbreviations: **ECMO**, extracorporeal membrane oxygenation; **IL-6**, Interleukin 6; **MV**, mechanical ventilation; **NMBAs**, continuous neuromuscular blocking agents; **no.**, number of patients; **NOACs**, new oral anticoagulants.

Supplementary Table 5. Complications in Critically Ill COVID-19 Patients with Delta Variant, According to Hospital Survivability

Variables	All cases n=105	Survived n=22	Died n=83	P value[a]
HAP, no. (%), n=100	91 (91.0)	16 (76.2)	75 (94.9)	0.019
ARDS, no. (%), n=100	95 (95.0)	17 (81.0)	78 (98.7)	0.007
Diffuse alveolar damage, no. (%), n=99	92 (92.9)	16 (80.0)	76 (96.2)	0.029

Secondary bacterial infections, no. (%), n=74	50 (67.6)	9 (47.4)	41 (74.5)	0.029
Sepsis and septic shock, no. (%), n=78	36 (46.2)	1 (4.8)	35 (61.4)	<0.001
Cardiac injury, no. (%), n=91	9 (9.9)	0.0 (0.0)	9 (12.5)	0.195
Arrhythmia, no. (%), n=100	23 (23.0)	1 (4.8)	22 (27.8)	0.038
Acute kidney injury, no. (%), n=104	35 (33.7)	0.0 (0.00	35 (42.7)	<0.001
Liver dysfunction, no. (%), n=99	22 (22.2)	1 (4.8)	21 (26.9)	0.037
Multi-organ failure, no. (%), n=100	45 (45.0)	0.0 (0.0)	45 (57.7)	<0.001
Thromboembolism, no. (%), n=96	2 (2.1)	0.0 (0.0)	2 (2.7)	>0.999
Gastrointestinal bleeding, no. (%), n=90	7 (6.7)	0.0 (0.0)	7 (8.4)	0.340
Pneumomediastinum, no. (%), n=102	5 (4.9)	0.0 (0.0)	5 (6.2)	0.581

[a] The comparison between patients who survived and those who died in the hospital.

Abbreviations: **ARDS**, acute respiratory distress syndrome; **HAP**, hospital-acquired pneumonia; **no.**, number of patients.

Supplementary Table 6. Pairwise Comparisons of AUROC of the Simplified RALE and the Severity Scoring Systems upon Admission for Predicting Hospital Mortality

Comparison	AUROC difference (95% CI)	SE	Z -statistic	p-value
**On admission/the 1st day of ICU admission**				
Simplified RALE and SOFA	0.015 (-0.117 to 0.148)	0.068	0.23	0.819
Simplified RALE and APACHE II	0.087 (-0.076 to 0.250)	0.083	1.05	0.294
Simplified RALE and CURB-65	0.046 (-0.110 to 0.203)	0.080	0.58	0.562

Simplified RALE and Serum IL-6	0.124 (-0.041 to 0.290)	0.084	1.48	0.140

Abbreviations: **APACHE II**, Acute Physiology and Chronic Health Evaluation II; **AUROC**, areas under the receiver operator characteristic curve; **CCI**, Charlson Comorbidity Index; **CI**, confidence interval; **CURB-65**, Confusion, Urea >7 mmol/L, Respiratory Rate ≥30 breaths/min, Blood Pressure <90 mm Hg (Systolic) or <60 mm Hg (Diastolic), and Age ≥65 Years Criteria; **IL-6**, Interleukin 6; **SOFA**, Sequential Organ Failure Assessment; **RALE**, Radiographic Assessment of Lung Edema.

Supplementary Table 7. Spearman's Correlation between the Simplified RALE Score and the Severity Scoring Systems upon Admission

		Simplified RALE	SOFA	APACHE II	CURB-65	PaO_2/FiO_2	Serum IL-6
Simplified RALE	Rs value	1.000	0.344	0.231	0.235	-0.158	0.226
	P value	NA	0.002	0.046	0.019	0.220	0.032
	N	105	79	75	99	62	90
SOFA	Rs value	0.344	1.000	0.455	0.438	-0.512	0.329
	P value	0.002	NA	0.000	0.000	0.000	0.004
	N	79	79	72	79	61	74
APACHE II	Rs value	0.231	0.455	1.000	0.683	-0.268	0.360
	P value	0.046	0.000	NA	0.000	0.037	0.002
	N	75	72	75	75	61	72
CURB-65	Rs value	0.235	0.438	0.683	1.000	-0.271	0.313
	P value	0.019	0.000	0.000	NA	0.033	0.003
	N	99	79	75	99	62	87
PaO_2/FiO_2	Rs value	-0.158	-0.512	-0.268	-0.271	1.000	-0.185

	P value	0.220	0.000	0.037	0.033	NA	0.149
	N	62	61	61	62	62	62
Serum IL-6	Rs value	0.226	0.329	0.360	0.313	-0.185	1.000
	P value	0.032	0.004	0.002	0.003	0.149	NA
	N	90	74	72	87	62	90

Abbreviations: **APACHE II**, Acute Physiology and Chronic Health Evaluation II; **AUROC**, areas under the receiver operator characteristic curves; **CCI**, Charlson Comorbidity Index; **CURB-65**, Confusion, Urea >7 mmol/L, Respiratory Rate ≥30 breaths/min, Blood Pressure <90 mm Hg (Systolic) or <60 mm Hg (Diastolic), and Age ≥65 Years Criteria; **IL-6**, Interleukin 6; **N**, number of patients; **NA**, not available; **PaO_2/FiO_2**, the ratio of partial pressure of oxygen in arterial blood to the fraction of inspiratory oxygen concentration; **Rs**, correlation coefficients; **SOFA**, Sequential Organ Failure Assessment; **RALE**, Radiographic Assessment of Lung Edema.

Supplementary Table 8. Multivariable Regression Analysis of Simplified RALE Score and Hospital Mortality in Critically Ill COVID-19 Patients with Delta Variant upon Admission, Adjusted for Baseline Confounders

Factors	Univariable logistic regression analyses[a]				Multivariable logistic regression analysis[b]			
	OR	95% CI for OR		p-value	AOR	95% CI for AOR		p-value
		Lower	Upper			Lower	Upper	
**Exposure variable**								
Initial chest imaging								
Simplified RALE score	1.938	1.393	2.697	<0.001	2.170	1.353	3.482	0.001
**Confounding variables**								
Inter-hospital transfers								
Endotracheal tube	2.180	0.454	10.645	0.330	NA	NA	NA	NA
Nasal cannula	0.077	0.008	0.783	0.030	NA	NA	NA	NA
Demographics								
Age (year)	1.039	1.005	1.074	0.023	NA	NA	NA	NA

Gender (male)	2.043	0.726	5.743	0.176	10.967	1.857	64.757	0.008
Comorbidities								
CCI score	1.401	1.027	1.912	0.033	1.564	1.030	2.374	0.036
Admission vital signs								
RR (breaths/min)	1.191	1.046	1.357	0.008	1.303	1.076	1.577	0.007
Admission labs								
Fibrinogen (g/L)	1.592	1.137	2.228	0.007	NA	NA	NA	NA
Constant					0.000			0.001

[a)] Each exposure and confounding variable was first analysed using a univariable logistic regression model. Variables with a P-value ≤0.10 in the univariable analysis and clinically significant factors were included in the multivariable logistic regression model. To ensure the robustness of our model, we evaluated multicollinearity among the predictor variables using the variance inflation factor (VIF) analysis. All VIF values were within acceptable limits, indicating no significant collinearity concerns. In addition, the final model demonstrated a good model fit, with -2 Log-Likelihood (-2LL) of 52.537 and a pseudo R^2 of 0.532, indicating strong explanatory power. The Hosmer-Lemeshow goodness-of-fit test yielded a χ^2 2 (8)=12.222, p-value = 0.142, suggesting excellent calibration and no evidence of poor fit.

[b)] The multivariable logistic regression model utilized a stepwise backward elimination approach. Variables were systematically removed from the full model until only those independently associated with hospital mortality remained.

Abbreviations: **AOR**, adjusted odds ratio; **CCI**, Charlson Comorbidity Index; **CI**, confidence interval; **NA**, not available; **OR**, odds ratio; **RALE**, Radiographic Assessment of Lung Edema; **RR**, respiration rate.

Supplementary Table 9. Multivariable Regression Analysis of Simplified RALE Score ≥Cut-off Value and Hospital Mortality in Critically Ill COVID-19 Patients with Delta Variant upon Admission, Adjusted for Baseline Confounders

Factors	Univariable logistic regression analyses				Multivariable logistic regression analysis			
	OR	95% CI for OR		p-value	AOR	95% CI for AOR		p-value
		Lower	Upper			Lower	Upper	
**Exposure variable**								
Initial chest imaging								
Simplified RALE ≥5.5[c]	13.000	3.786	44.637	<0.001	12.977	2.562	65.719	0.002

**Confounding variables**								
Inter-hospital transfers								
Endotracheal tube	2.180	0.454	10.645	0.330	NA	NA	NA	NA
Nasal cannula	0.077	0.008	0.783	0.030	NA	NA	NA	NA
Demographics								
Age (year)	1.039	1.005	1.074	0.023	NA	NA	NA	NA
Gender (male)	2.043	0.726	5.743	0.176	12.126	2.045	71.909	0.006
Comorbidities								
CCI score	1.401	1.027	1.912	0.033	1.522	1.011	2.292	0.044
Admission vital signs								
RR (breaths/min)	1.191	1.046	1.357	0.008	1.292	1.074	1.553	0.007
Admission labs								
Fibrinogen (g/L)	1.592	1.137	2.228	0.007	NA	NA	NA	NA
Constant					0.000			0.001

[a)] Each exposure and confounding variable was first analysed using a univariable logistic regression model. Variables with a P-value ≤0.10 in the univariable analysis and clinically significant factors were included in the multivariable logistic regression model. To ensure the robustness of our model, we evaluated multicollinearity among the predictor variables using the variance inflation factor (VIF) analysis. All VIF values were within acceptable limits, indicating no significant collinearity concerns. In addition, the final model demonstrated a good model fit, with -2 Log-Likelihood (-2LL) of 55.089 and a pseudo R^2 of 0.501, indicating strong explanatory power. The Hosmer-Lemeshow goodness-of-fit test yielded a χ^2 2 (8)=12.290, p-value = 0.139, suggesting excellent calibration and no evidence of poor fit

[b)] The multivariable logistic regression model utilized a stepwise backward elimination approach. Variables were systematically removed from the full model until only those independently associated with hospital mortality remained.

[c)] The numbers represent the best cut-off value determined by analysing each variable's receiver operator characteristic curve.

Abbreviations: **AOR**, adjusted odds ratio; **CCI**, Charlson Comorbidity Index; **CI**, confidence interval; **NA**, not available; **OR**, odds ratio; **RALE**, Radiographic Assessment of Lung Edema; **RR**, respiration rate.

Supplementary Table 10. Multivariable Regression Analysis of SOFA Score and Hospital Mortality in Critically Ill COVID-19 Patients with Delta Variant upon Admission, Adjusted for Baseline Confounders

Factors	Univariable logistic regression analyses[a]				Multivariable logistic regression analysis[b]			
	OR	95% CI for OR		p-value	AOR	95% CI for AOR		p-value
		Lower	Upper			Lower	Upper	
**Exposure variable**								
Admission severity of illness								
SOFA score	1.680	1.158	2.438	0.006	1.417	0.939	2.139	0.097
**Confounding variables**								
Inter-hospital transfers								
Endotracheal tube	2.180	0.454	10.645	0.330	NA	NA	NA	NA
Nasal cannula	0.077	0.008	0.783	0.030	NA	NA	NA	NA
Demographics								
Age (year)	1.039	1.005	1.074	0.023	NA	NA	NA	NA
Gender (male)	2.043	0.726	5.743	0.176	3.783	0.869	16.463	0.076
Comorbidities								
CCI score	1.401	1.027	1.912	0.033	NA	NA	NA	NA
Admission vital signs								
RR (breaths/min)	1.191	1.046	1.357	0.008	1.204	0.988	1.466	0.066
Admission labs								
Fibrinogen (g/L)	1.592	1.137	2.228	0.007	NA	NA	NA	NA
Constant					0.007			0.044

[a)] Each exposure and confounding variable was first analysed using a univariable logistic regression model. Variables with a P-value ≤0.10 in the univariable analysis and clinically significant factors were included in the multivariable logistic regression model. To ensure the robustness of our model, we evaluated multicollinearity among the predictor variables using the variance inflation factor (VIF) analysis. All VIF values were within acceptable limits, indicating no significant collinearity concerns. In addition, the final model demonstrated a good model fit, with -2 Log-Likelihood (-2LL) of 55.248 and a pseudo R^2 of 0.323, indicating strong explanatory power. The Hosmer-Lemeshow goodness-of-fit test yielded a χ^2 2 (8)=7.971, p-value = 0.436, suggesting excellent calibration and no evidence of poor fit

b) The multivariable logistic regression model utilized a stepwise backward elimination approach. Variables were systematically removed from the full model until only those independently associated with hospital mortality remained.
Abbreviations: **AOR**, adjusted odds ratio; **CCI**, Charlson Comorbidity Index; **CI**, confidence interval **NA**, not available; **OR**, odds ratio; **RR**, respiration rate; **SOFA**, Sequential Organ Failure Assessment.

Supplementary Table 11. Multivariable Regression Analysis of SOFA Score ≥Cut-off Value and Hospital Mortality in Critically Ill COVID-19 Patients with Delta Variant upon Admission, Adjusted for Baseline Confounders

Factors	Univariable logistic regression analyses[a]				Multivariable logistic regression analysis[b]			
	OR	95% CI for OR		p-value	AOR	95% CI for AOR		p-value
		Lower	Upper			Lower	Upper	
**Exposure variable**								
Admission severity of illness								
SOFA score ≥ 3.5[c]	6.667	1.707	26.042	0.006	4.450	0.801	24.726	0.088
**Confounding variables**								
Inter-hospital transfers								
Endotracheal tube	2.180	0.454	10.645	0.330	NA	NA	NA	NA
Nasal cannula	0.077	0.008	0.783	0.030	0.000	0.000	NA	0.999
Demographics								
Age (year)	1.039	1.005	1.074	0.023	NA	NA	NA	NA
Gender (male)	2.043	0.726	5.743	0.176	7.019	1.182	41.684	0.032
Comorbidities								
CCI score	1.401	1.027	1.912	0.033	1.498	0.960	2.338	0.075
Admission vital signs								
RR (breaths/min)	1.191	1.046	1.357	0.008	1.157	0.954	1.403	0.139
Admission labs								

Fibrinogen (g/L)	1.592	1.137	2.228	0.007	NA	NA	NA	NA
Constant					0.013			0.103

a) Each exposure and confounding variable was first analysed using a univariable logistic regression model. Variables with a P-value ≤0.10 in the univariable analysis and clinically significant factors were included in the multivariable logistic regression model. To ensure the robustness of our model, we evaluated multicollinearity among the predictor variables using the variance inflation factor (VIF) analysis. All VIF values were within acceptable limits, indicating no significant collinearity concerns. In addition, the final model demonstrated a good model fit, with -2 Log-Likelihood (-2LL) of 45.908 and a pseudo R^2 of 0.475, indicating strong explanatory power. The Hosmer-Lemeshow goodness-of-fit test yielded a χ^2 2 (8)=9.745, p-value = 0.283, suggesting excellent calibration and no evidence of poor fit

b) The multivariable logistic regression model utilized a stepwise backward elimination approach. Variables were systematically removed from the full model until only those independently associated with hospital mortality remained.

c) The numbers represent the best cut-off value determined by analysing each variable's receiver operator characteristic curve.

Abbreviations: **AOR**, adjusted odds ratio; **CCI**, Charlson Comorbidity Index; **CI**, confidence interval **NA**, not available; **OR**, odds ratio; **RR**, respiration rate; **SOFA**, Sequential Organ Failure Assessment.

Supplementary Table 12. Multivariable Regression Analysis of APACHE II Score and Hospital Mortality in Critically Ill COVID-19 Patients with Delta Variant upon Admission, Adjusted for Baseline Confounders

Factors	Univariable logistic regression analyses[a]				Multivariable logistic regression analysis[b]			
	OR	95% CI for OR		p-value	AOR	95% CI for AOR		p-value
		Lower	Upper			Lower	Upper	
**Exposure variable**								
Admission severity of illness								
APACHE II score	1.117	0.988	1.261	0.077	NA	NA	NA	NA
**Confounding variables**								
Inter-hospital transfers								
Endotracheal tube	2.180	0.454	10.645	0.330	NA	NA	NA	NA
Nasal cannula	0.077	0.008	0.783	0.030	0.000	0.000	NA	0.999
Demographics								

Age (year)	1.039	1.005	1.074	0.023	NA	NA	NA	NA
Gender (male)	2.043	0.726	5.743	0.176	5.079	1.019	25.326	0.047
Comorbidities								
CCI score	1.401	1.027	1.912	0.033	1.424	.945	2.144	0.091
Admission vital signs								
RR (breaths/min)	1.191	1.046	1.357	0.008	1.200	1.003	1.436	0.046
Admission labs								
Fibrinogen (g/L)	1.592	1.137	2.228	0.007	NA	NA	NA	NA
Constant					0.010	-	-	0.064

a) Each exposure and confounding variable was first analysed using a univariable logistic regression model. Variables with a P-value ≤0.10 in the univariable analysis and clinically significant factors were included in the multivariable logistic regression model. To ensure the robustness of our model, we evaluated multicollinearity among the predictor variables using the variance inflation factor (VIF) analysis. All VIF values were within acceptable limits, indicating no significant collinearity concerns. In addition, the final model demonstrated a good model fit, with -2 Log-Likelihood (-2LL) of 50.797 and a pseudo R^2 of 0.412, indicating strong explanatory power. The Hosmer-Lemeshow goodness-of-fit test yielded a χ^2 2 (8)=4.779, p-value = 0.781, suggesting excellent calibration and no evidence of poor fit

b) The multivariable logistic regression model utilized a stepwise backward elimination approach. Variables were systematically removed from the full model until only those independently associated with hospital mortality remained.

Abbreviations: **AOR**, adjusted odds ratio; **APACHE II**, Acute Physiology and Chronic Health Evaluation II; **CCI**, Charlson Comorbidity Index; **CI**, confidence interval; **NA**, not available; **OR**, odds ratio; **RR**, respiration rate.

Supplementary Table 13. Multivariable Regression Analysis of APACHE II Score ≥Cut-off Value and Hospital Mortality in Critically Ill COVID-19 Patients with Delta Variant upon Admission, Adjusted for Baseline Confounders

Factors	Univariable logistic regression analyses[a]				Multivariable logistic regression analysis[b]			
	OR	95% CI for OR		p-value	AOR	95% CI for AOR		p-value
		Lower	Upper			Lower	Upper	
**Exposure variable**								
Admission severity of illness								

APACHE II score ≥ 11.5[c]	7.692	0.947	62.490	0.056	NA	NA	NA	NA
**Confounding variables**								
Inter-hospital transfers								
Endotracheal tube	2.180	0.454	10.645	0.330	NA	NA	NA	NA
Nasal cannula	0.077	0.008	0.783	0.030	0.000	0.000	NA	0.999
Demographics								
Age (year)	1.039	1.005	1.074	0.023	NA	NA	NA	NA
Gender (male)	2.043	0.726	5.743	0.176	5.079	1.019	25.326	0.047
Comorbidities								
CCI score	1.401	1.027	1.912	0.033	1.424	0.945	2.144	0.091
Admission vital signs								
RR (breaths/min)	1.191	1.046	1.357	0.008	1.200	1.003	1.436	0.046
Admission labs								
Fibrinogen (g/L)	1.592	1.137	2.228	0.007	NA	NA	NA	NA
Constant					0.010			0.064

[a)] Each exposure and confounding variable was first analysed using a univariable logistic regression model. Variables with a P-value ≤0.10 in the univariable analysis and clinically significant factors were included in the multivariable logistic regression model. To ensure the robustness of our model, we evaluated multicollinearity among the predictor variables using the variance inflation factor (VIF) analysis. All VIF values were within acceptable limits, indicating no significant collinearity concerns. In addition, the final model demonstrated a good model fit, with -2 Log-Likelihood (-2LL) of 50.797 and a pseudo R^2 of 0.412, indicating strong explanatory power. The Hosmer-Lemeshow goodness-of-fit test yielded a χ^2 2 (8)=4.779, p-value = 0.781, suggesting excellent calibration and no evidence of poor fit

[b)] The multivariable logistic regression model utilized a stepwise backward elimination approach. Variables were systematically removed from the full model until only those independently associated with hospital mortality remained.

[c)] The numbers represent the best cut-off value determined by analysing each variable's receiver operator characteristic curve.

Abbreviations: **AOR**, adjusted odds ratio; **APACHE II**, Acute Physiology and Chronic Health Evaluation II; **CCI**, Charlson Comorbidity Index; **CI**, confidence interval; **NA**, not available; **OR**, odds ratio; **RR**, respiration rate.

Supplementary Table 14. Multivariable Regression Analysis of CURB-65 Score and Hospital Mortality in Critically Ill COVID-19 Patients with Delta Variant upon Admission, Adjusted for Baseline Confounders

Factors	Univariable logistic regression analyses				Multivariable logistic regression analysis			
	OR	95% CI for OR		p-value	AOR	95% CI for AOR		p-value
		Lower	Upper			Lower	Upper	
**Exposure variable**								
Admission severity of illness								
CURB-65 score	4.436	1.940	10.146	<0.001	4.316	1.684	11.059	0.002
**Confounding variables**								
Inter-hospital transfers								
Endotracheal tube	2.180	0.454	10.645	0.330	NA	NA	NA	NA
Nasal cannula	0.077	0.008	0.783	0.030	0.000	0.000	NA	0.999
Demographics								
Age (year)	1.039	1.005	1.074	0.023	NA	NA	NA	NA
Gender (male)	2.043	0.726	5.743	0.176	NA	NA	NA	NA
Comorbidities								
CCI score	1.401	1.027	1.912	0.033	NA	NA	NA	NA
Admission vital signs								
RR (breaths/min)	1.191	1.046	1.357	0.008	NA	NA	NA	NA
Admission labs								
Fibrinogen (g/L)	1.592	1.137	2.228	0.007	1.640	1.049	2.565	0.030
Constant					0.094			0.051

[a)] Each exposure and confounding variable was first analysed using a univariable logistic regression model. Variables with a P-value ≤0.10 in the univariable analysis and clinically significant factors were included in the multivariable logistic regression model. To ensure the robustness of our model, we evaluated multicollinearity among the predictor variables using the variance inflation factor (VIF) analysis. All VIF values were within acceptable limits, indicating no significant collinearity concerns. In addition,

the final model demonstrated a good model fit, with -2 Log-Likelihood (-2LL) of 59.227 and a pseudo R^2 of 0.446, indicating strong explanatory power. The Hosmer-Lemeshow goodness-of-fit test yielded a χ^2 2 (8)=14.005, p-value = 0.082, suggesting excellent calibration and no evidence of poor fit

b) The multivariable logistic regression model utilized a stepwise backward elimination approach. Variables were systematically removed from the full model until only those independently associated with hospital mortality remained.

Abbreviations: **AOR**, adjusted odds ratio; **CCI**, Charlson Comorbidity Index; **CI**, confidence interval; **CURB-65**, Confusion, Urea >7 mmol/L, Respiratory Rate ≥30 breaths/min, Blood Pressure <90 mm Hg (Systolic) or <60 mm Hg (Diastolic), Age ≥65 Years; **NA**, not available; **OR**, odds ratio; **RR**, respiration rate.

Supplementary Table 15. Multivariable Regression Analysis of CURB-65 Score ≥Cut-off Value and Hospital Mortality in Critically Ill COVID-19 Patients with Delta Variant upon Admission, Adjusted for Baseline Confounders

Factors	Univariable logistic regression analyses[a]				Multivariable logistic regression analysis[b]			
	OR	95% CI for OR		p-value	AOR	95% CI for AOR		p-value
		Lower	Upper			Lower	Upper	
**Exposure variable**								
Admission severity of illness								
CURB-65 score ≥ 0.5[c]	8.875	2.835	27.786	<0.001	6.283	1.568	25.185	0.009
**Confounding variables**								
Inter-hospital transfers								
Endotracheal tube	2.180	0.454	10.645	0.330	NA	NA	NA	NA
Nasal cannula	0.077	0.008	0.783	0.030	NA	NA	NA	NA
Demographics								
Age (year)	1.039	1.005	1.074	0.023	NA	NA	NA	NA
Gender (male)	2.043	0.726	5.743	0.176	4.137	1.065	16.063	0.040
Comorbidities								
CCI score	1.401	1.027	1.912	0.033	NA	NA	NA	NA

Admission vital signs								
RR (breaths/min)	1.191	1.046	1.357	0.008	1.168	0.986	1.385	0.073
Admission labs								
Fibrinogen (g/L)	1.592	1.137	2.228	0.007	NA	NA	NA	NA
Constant					0.010			0.036

[a] Each exposure and confounding variable was first analysed using a univariable logistic regression model. Variables with a P-value ≤0.10 in the univariable analysis and clinically significant factors were included in the multivariable logistic regression model. To ensure the robustness of our model, we evaluated multicollinearity among the predictor variables using the variance inflation factor (VIF) analysis. All VIF values were within acceptable limits, indicating no significant collinearity concerns. In addition, the final model demonstrated a good model fit, with -2 Log-Likelihood (-2LL) of 65.738 and a pseudo R^2 of 0.358, indicating strong explanatory power. The Hosmer-Lemeshow goodness-of-fit test yielded a χ^2 2 (8)=3.898, p-value = 0.866, suggesting excellent calibration and no evidence of poor fit

[b] The multivariable logistic regression model utilized a stepwise backward elimination approach. Variables were systematically removed from the full model until only those independently associated with hospital mortality remained.

[c] The numbers represent the best cut-off value determined by analysing each variable's receiver operator characteristic curve.

Abbreviations: **AOR**, adjusted odds ratio; **CCI**, Charlson Comorbidity Index; **CI**, confidence interval; **CURB-65**, Confusion, Urea >7 mmol/L, Respiratory Rate ≥30 breaths/min, Blood Pressure <90 mm Hg (Systolic) or <60 mm Hg (Diastolic), Age ≥65 Years; **NA**, not available; **OR**, odds ratio; **RR**, respiration rate.

Supplementary Table 16. Multivariable Regression Analysis of First-day PaO2/FiO2 Ratio and Hospital Mortality in Critically Ill COVID-19 Patients with Delta Variant upon Admission, Adjusted for Baseline Confounders

Factors	Univariable logistic regression analyses				Multivariable logistic regression analysis			
	OR	95% CI for OR		p-value	AOR	95% CI for AOR		p-value
		Lower	Upper			Lower	Upper	
**Exposure variable**								
The 1st-day gas exchange								
PaO_2/FiO_2 ratio	0.993	0.983	1.002	0.140	NA	NA	NA	NA
**Confounding variables**								
Inter-hospital transfers								

Endotracheal tube	2.180	0.454	10.645	0.330	NA	NA	NA	NA
Nasal cannula	0.077	0.008	0.783	0.030	NA	NA	NA	NA
Demographics								
Age (year)	1.039	1.005	1.074	0.023	NA	NA	NA	NA
Gender (male)	2.043	0.726	5.743	0.176	4.505	0.805	25.199	0.087
Comorbidities								
CCI score	1.401	1.027	1.912	0.033	NA	NA	NA	NA
Admission vital signs								
RR (breaths/min)	1.191	1.046	1.357	0.008	1.172	0.977	1.405	0.088
Admission labs								
Fibrinogen (g/L)	1.592	1.137	2.228	0.007	NA	NA	NA	
Constant					0.042			0.200

[a)] Each exposure and confounding variable was first analysed using a univariable logistic regression model. Variables with a P-value ≤0.10 in the univariable analysis and clinically significant factors were included in the multivariable logistic regression model. To ensure the robustness of our model, we evaluated multicollinearity among the predictor variables using the variance inflation factor (VIF) analysis. All VIF values were within acceptable limits, indicating no significant collinearity concerns. In addition, the final model demonstrated a good model fit, with -2 Log-Likelihood (-2LL) of 46.749 and a pseudo R^2 of 0.170, indicating strong explanatory power. The Hosmer-Lemeshow goodness-of-fit test yielded a χ^2 2 (8)=4.400, p-value = 0.819, suggesting excellent calibration and no evidence of poor fit

[b)] The multivariable logistic regression model utilized a stepwise backward elimination approach. Variables were systematically removed from the full model until only those independently associated with hospital mortality remained.

Abbreviations: **AOR**, adjusted odds ratio; **CCI**, Charlson Comorbidity Index; **CI**, confidence interval; **NA**, not available; **OR**, odds ratio; **PaO_2/FiO_2**, arterial oxygen partial pressure to inspired oxygen fraction ratio; **RR**, respiration rate.

Supplementary Table 17. Multivariable Regression Analysis of First-day PaO2/FiO2 Ratio ≥Cut-off Value and Hospital Mortality in Critically Ill COVID-19 Patients with Delta Variant upon Admission, Adjusted for Baseline Confounders

Factors	Univariable logistic regression analyses[a]				Multivariable logistic regression analysis[b]			
	OR	95% CI for OR		p-value	AOR	95% CI for AOR		p-value
		Lower	Upper			Lower	Upper	
**Exposure variable**								
The 1st-day gas exchange								
$PaO_2/FiO_2 \geq 119.5$ mmHg[c]	0.179	0.045	0.716	0.015	0.166	0.037	0.736	0.018
**Confounding variables**								
Inter-hospital transfers								
Endotracheal tube	2.180	0.454	10.645	0.330	NA	NA	NA	NA
Nasal cannula	0.077	0.008	0.783	0.030	NA	NA	NA	NA
Demographics								
Age (year)	1.039	1.005	1.074	0.023	NA	NA	NA	NA
Gender (male)	2.043	0.726	5.743	0.176	3.267	0.579	18.426	0.180
Comorbidities								
CCI score	1.401	1.027	1.912	0.033	NA	NA	NA	NA
Admission vital signs								
RR (breaths/min)	1.191	1.046	1.357	0.008	NA	NA	NA	NA
Admission labs								
Fibrinogen (g/L)	1.592	1.137	2.228	0.007	NA	NA	NA	NA
Constant					6.356			0.001

[a]) Each exposure and confounding variable was first analysed using a univariable logistic regression model. Variables with a P-value ≤0.10 in the univariable analysis and clinically significant factors were included in the multivariable logistic regression model. To ensure the robustness of our model, we evaluated multicollinearity among the predictor variables using the variance inflation factor (VIF) analysis. All VIF values were within acceptable limits, indicating no significant collinearity concerns. In addition, the final model demonstrated a good model fit, with -2 Log-Likelihood (-2LL) of 44.564 and a pseudo R^2 of 0.226, indicating strong explanatory power. The Hosmer-Lemeshow goodness-of-fit test yielded a χ^2 2 (8)=0.050, p-value = 0.975, suggesting excellent calibration and no evidence of poor fit

[b] The multivariable logistic regression model utilized a stepwise backward elimination approach. Variables were systematically removed from the full model until only those independently associated with hospital mortality remained.
[c] The numbers represent the best cut-off value determined by analysing each variable's receiver operator characteristic curve.
Abbreviations: **AOR**, adjusted odds ratio; **CCI**, Charlson Comorbidity Index; **CI**, confidence interval; **IL-6**, interleukin 6; **NA**, not available; **OR**, odds ratio; **RR**, respiration rate.

Supplementary Table 18. Multivariable Regression Analysis of IL-6 Level and Hospital Mortality in Critically Ill COVID-19 Patients with Delta Variant upon Admission, Adjusted for Baseline Confounders

Factors	Univariable logistic regression analyses				Multivariable logistic regression analysis			
	OR	95% CI for OR		p-value	AOR	95% CI for AOR		p-value
		Lower	Upper			Lower	Upper	
**Exposure variable**								
The 1st-day Laboratory investigations								
IL-6 level	1.002	0.997	1.007	0.334	NA	NA	NA	NA
**Confounding variables**								
Inter-hospital transfers								
Endotracheal tube	2.180	0.454	10.645	0.330	NA	NA	NA	NA
Nasal cannula	0.077	0.008	0.783	0.030	0.000	0.000	NA	0.999
Demographics								
Age (year)	1.039	1.005	1.074	0.023	NA	NA	NA	NA
Gender (male)	2.043	0.726	5.743	0.176	5.314	1.235	22.877	0.025
Comorbidities								
CCI score	1.401	1.027	1.912	0.033	1.579	1.046	2.383	0.030
Admission vital signs								

RR (breaths/min)	1.191	1.046	1.357	0.008	1.218	1.032	1.437	0.020
Admission labs								
Fibrinogen (g/L)	1.592	1.137	2.228	0.007	NA	NA	NA	NA
Constant					0.005			0.023

[a)] Each exposure and confounding variable was first analysed using a univariable logistic regression model. Variables with a P-value ≤0.10 in the univariable analysis and clinically significant factors were included in the multivariable logistic regression model. To ensure the robustness of our model, we evaluated multicollinearity among the predictor variables using the variance inflation factor (VIF) analysis. All VIF values were within acceptable limits, indicating no significant collinearity concerns. In addition, the final model demonstrated a good model fit, with -2 Log-Likelihood (-2LL) of 58.719 and a pseudo R^2 of 0.394, indicating strong explanatory power. The Hosmer-Lemeshow goodness-of-fit test yielded a χ^2 2 (8)=7.135, p-value = 0.522, suggesting excellent calibration and no evidence of poor fit

[b)] The multivariable logistic regression model utilized a stepwise backward elimination approach. Variables were systematically removed from the full model until only those independently associated with hospital mortality remained.

Abbreviations: **AOR**, adjusted odds ratio; **CCI**, Charlson Comorbidity Index; **CI**, confidence interval; **IL-6**, interleukin 6; **NA**, not available; **OR**, odds ratio; **RR**, respiration rate.

Supplementary Table 19. Multivariable Regression Analysis of IL-6 Level ≥Cut-off Value and Hospital Mortality in Critically Ill COVID-19 Patients with Delta Variant upon Admission, Adjusted for Baseline Confounders

Factors	Univariable logistic regression analyses				Multivariable logistic regression analysis			
	OR	95% CI for OR		p-value	AOR	95% CI for AOR		p-value
		Lower	Upper			Lower	Upper	
**Exposure variable**								
The 1st-day Laboratory investigations								
IL-6 level ≥ 15.8 pg/mL[c]	3.955	1.345	11.623	0.012	NA	NA	NA	NA
**Confounding variables**								
Inter-hospital transfers								

Endotracheal tube	2.180	0.454	10.645	0.330	NA	NA	NA	NA
Nasal cannula	0.077	0.008	0.783	0.030	0.000	0.000	NA	0.999
Demographics								
Age (year)	1.039	1.005	1.074	0.023	NA	NA	NA	NA
Gender (male)	2.043	0.726	5.743	0.176	5.314	1.235	22.877	0.025
Comorbidities								
CCI score	1.401	1.027	1.912	0.033	1.579	1.046	2.383	0.030
Admission vital signs								
RR (breaths/min)	1.191	1.046	1.357	0.008	1.218	1.032	1.437	0.020
Admission labs								
Fibrinogen (g/L)	1.592	1.137	2.228	0.007	NA	NA	NA	NA
Constant					0.005			0.023

[a] Each exposure and confounding variable was first analysed using a univariable logistic regression model. Variables with a P-value ≤0.10 in the univariable analysis and clinically significant factors were included in the multivariable logistic regression model. To ensure the robustness of our model, we evaluated multicollinearity among the predictor variables using the variance inflation factor (VIF) analysis. All VIF values were within acceptable limits, indicating no significant collinearity concerns. In addition, the final model demonstrated a good model fit, with -2 Log-Likelihood (-2LL) of 58.719 and a pseudo R^2 of 0.394, indicating strong explanatory power. The Hosmer-Lemeshow goodness-of-fit test yielded a χ^2 2 (8)=7.135, p-value = 0.522, suggesting excellent calibration and no evidence of poor fit

[b] The multivariable logistic regression model utilized a stepwise backward elimination approach. Variables were systematically removed from the full model until only those independently associated with hospital mortality remained.

[c] The numbers represent the best cut-off value determined by analysing each variable's receiver operator characteristic curve.

Abbreviations: **AOR**, adjusted odds ratio; **CCI**, Charlson Comorbidity Index; **CI**, confidence interval; **IL-6**, interleukin 6; **NA**, not available; **OR**, odds ratio; **RR**, respiration rate.

Supplementary Table 20. Multivariable Regression Analysis of Simplified RALE Score and Hospital Mortality in Critically Ill COVID-19 Patients with Delta Variant upon Admission, Adjusted for Baseline and Mediator Confounders

Factors	Univariable logistic regression analyses[a]				Multivariable logistic regression analysis[b]			
	OR	95% CI for OR		p-value	AOR	95% CI for AOR		p-value
		Lower	Upper			Lower	Upper	
**Exposure variable**								
Initial chest imaging								
Simplified RALE score	1.938	1.393	2.697	<0.001	1.934	1.220	3.066	0.005
**Confounding variables**								
Inter-hospital transfers								
Endotracheal tube	2.180	0.454	10.645	0.330	NA	NA	NA	NA
Nasal cannula	0.077	0.008	0.783	0.030	NA	NA	NA	NA
Demographics								
Age (year)	1.039	1.005	1.074	0.023	NA	NA	NA	NA
Gender (male)	2.043	0.726	5.743	0.176	5.281	1.027	27.146	0.046
Comorbidities								
CCI score	1.401	1.027	1.912	0.033	NA	NA	NA	NA
Admission vital signs								
RR (breaths/min)	1.191	1.046	1.357	0.008	1.258	1.043	1.519	0.017
Admission labs								
Fibrinogen (g/L)	1.592	1.137	2.228	0.007	NA	NA	NA	NA
1st-day respiratory support								
Bag valve mask	0.375	0.141	0.999	0.050	NA	NA	NA	NA
High-flow nasal cannula	2.523	0.838	7.598	0.100	NA	NA	NA	NA
Adjunctive therapies								
Antiviral drugs	0.437	0.147	1.300	0.136	NA	NA	NA	NA
NMBAs	8.421	2.870	24.707	<0.001	NA	NA	NA	NA

Renal replacement therapy	5.815	0.732	46.220	0.096	NA	NA	NA	NA
Complications								
HAP	18.353	1.928	174.691	0.011	NA	NA	NA	NA
ARDS	6.333	1.290	31.090	0.023	NA	NA	NA	NA
Constant					0.000			0.003

[a)] Each exposure and confounding variable was first analysed using a univariable logistic regression model. Variables with a P-value ≤0.10 in the univariable analysis and clinically significant factors were included in the multivariable logistic regression model. To ensure the robustness of our model, we evaluated multicollinearity among the predictor variables using the variance inflation factor (VIF) analysis. All VIF values were within acceptable limits, indicating no significant collinearity concerns. In addition, the final model demonstrated a good model fit, with -2 Log-Likelihood (-2LL) of 50.415 and a pseudo R^2 of 0.408, indicating strong explanatory power. The Hosmer-Lemeshow goodness-of-fit test yielded a χ^2 2 (8)=11.939, p-value = 0.103, suggesting excellent calibration and no evidence of poor fit.

[b)] The multivariable logistic regression model utilized a stepwise backward elimination approach. Variables were systematically removed from the full model until only those independently associated with hospital mortality remained.

Abbreviations: **AOR**, adjusted odds ratio; **ARDS**, acute respiratory distress syndrome; **CCI**, Charlson Comorbidity Index; **CI**, confidence interval; **HAP**, hospital-acquired pneumonia; **NA**, not available; **NMBAs**, continuous neuromuscular blocking agents; **OR**, odds ratio; **RALE**, Radiographic Assessment of Lung Edema; **RR**, respiration rate.

Supplementary Table 21. Multivariable Regression Analysis of Simplified RALE Score ≥Cut-off Value and Hospital Mortality in Critically Ill COVID-19 Patients with Delta Variant upon Admission, Adjusted for Baseline and Mediator Confounders

Factors	Univariable logistic regression analyses				Multivariable logistic regression analysis			
	OR	95% CI for OR		p-value	AOR	95% CI for AOR		p-value
		Lower	Upper			Lower	Upper	
**Exposure variable**								
Initial chest imaging								
Simplified RALE ≥5.5[c]	13.000	3.786	44.637	<0.001	6.665	1.020	43.560	0.048
**Confounding variables**								
Inter-hospital transfers								
Endotracheal tube	2.180	0.454	10.645	0.330	NA	NA	NA	NA
Nasal cannula	0.077	0.008	0.783	0.030	NA	NA	NA	NA

Demographics								
Age (year)	1.039	1.005	1.074	0.023	NA	NA	NA	NA
Gender (male)	2.043	0.726	5.743	0.176	4.845	0.821	28.573	0.081
Comorbidities								
CCI score	1.401	1.027	1.912	0.033	NA	NA	NA	NA
Admission vital signs								
RR (breaths/min)	1.191	1.046	1.357	0.008	1.228	1.018	1.482	0.032
Admission labs								
Fibrinogen (g/L)	1.592	1.137	2.228	0.007	1.475	0.933	2.333	0.097
1st-day respiratory support								
Bag valve mask	0.375	0.141	0.999	0.050	NA	NA	NA	NA
High-flow nasal cannula	2.523	0.838	7.598	0.100	NA	NA	NA	NA
Adjunctive therapies								
Antiviral drugs	0.437	0.147	1.300	0.136	NA	NA	NA	NA
NMBAs	8.421	2.870	24.707	<0.001	4.099	0.887	18.942	0.071
Renal replacement therapy	5.815	0.732	46.220	0.096	NA	NA	NA	NA
Complications								
HAP	18.353	1.928	174.691	0.011	NA	NA	NA	NA
ARDS	6.333	1.290	31.090	0.023	NA	NA	NA	NA
Constant					0.000			0.003

[a)] Each exposure and confounding variable was first analysed using a univariable logistic regression model. Variables with a P-value ≤0.10 in the univariable analysis and clinically significant factors were included in the multivariable logistic regression model. To ensure the robustness of our model, we evaluated multicollinearity among the predictor variables using the variance inflation factor (VIF) analysis. All VIF values were within acceptable limits, indicating no significant collinearity concerns. In addition, the final model demonstrated a good model fit, with -2 Log-Likelihood (-2LL) of 46.127 and a pseudo R^2 of 0.475, indicating strong explanatory power. The Hosmer-Lemeshow goodness-of-fit test yielded a χ^2 2 (8)=15.199, p-value = 0.034, indicating that the model is not a perfect fit.

[b)] The multivariable logistic regression model utilized a stepwise backward elimination approach. Variables were systematically removed from the full model until only those independently associated with hospital mortality remained.

[c)] The numbers represent the best cut-off value determined by analysing each variable's receiver operator characteristic curve.

Abbreviations: **AOR**, adjusted odds ratio; **ARDS**, acute respiratory distress syndrome; **CCI**, Charlson Comorbidity Index; **CI**, confidence interval; **HAP**, hospital-acquired

pneumonia; **NA**, not available; **NMBAs**, continuous neuromuscular blocking agents; **OR**, odds ratio; **RALE**, Radiographic Assessment of Lung Edema; **RR**, respiration rate.

Supplementary Table 22. Multivariable Regression Analysis of SOFA Score and Hospital Mortality in Critically Ill COVID-19 Patients with Delta Variant upon Admission, Adjusted for Baseline and Mediator Confounders

Factors	Univariable logistic regression analyses[a]				Multivariable logistic regression analysis[b]			
	OR	95% CI for OR		p-value	AOR	95% CI for AOR		p-value
		Lower	Upper			Lower	Upper	
**Exposure variable**								
Admission severity of illness								
SOFA score	1.680	1.158	2.438	0.006	NA	NA	NA	NA
**Confounding variables**								
Inter-hospital transfers								
Endotracheal tube	2.180	0.454	10.645	0.330	NA	NA	NA	NA
Nasal cannula	0.077	0.008	0.783	0.030	NA	NA	NA	NA
Demographics								
Age (year)	1.039	1.005	1.074	0.023	NA	NA	NA	NA
Gender (male)	2.043	0.726	5.743	0.176	NA	NA	NA	NA
Comorbidities								
CCI score	1.401	1.027	1.912	0.033	NA	NA	NA	NA
Admission vital signs								
RR (breaths/min)	1.191	1.046	1.357	0.008	1.214	0.979	1.505	0.077
Admission labs								
Fibrinogen (g/L)	1.592	1.137	2.228	0.007	NA	NA	NA	NA
1st-day respiratory support								
Bag valve mask	0.375	0.141	0.999	0.050	NA	NA	NA	NA
High-flow nasal cannula	2.523	0.838	7.598	0.100	NA	NA	NA	NA

Adjunctive therapies								
Antiviral drugs	0.437	0.147	1.300	0.136	NA	NA	NA	NA
NMBAs	8.421	2.870	24.707	<0.001	14.179	2.441	82.365	0.003
Renal replacement therapy	5.815	0.732	46.220	0.096	NA	NA	NA	NA
Complications								
HAP	18.353	1.928	174.691	0.011	NA	NA	NA	NA
ARDS	6.333	1.290	31.090	0.023	NA	NA	NA	NA
Constant					0.009			0.089

a) Each exposure and confounding variable was first analysed using a univariable logistic regression model. Variables with a P-value ≤0.10 in the univariable analysis and clinically significant factors were included in the multivariable logistic regression model. To ensure the robustness of our model, we evaluated multicollinearity among the predictor variables using the variance inflation factor (VIF) analysis. All VIF values were within acceptable limits, indicating no significant collinearity concerns. In addition, the final model demonstrated a good model fit, with -2 Log-Likelihood (-2LL) of 36.724 and a pseudo R^2 of 0.434, indicating strong explanatory power. The Hosmer-Lemeshow goodness-of-fit test yielded a χ^2 2 (8)=8.409, p-value = 0.395, suggesting excellent calibration and no evidence of poor fit.

b) The multivariable logistic regression model utilized a stepwise backward elimination approach. Variables were systematically removed from the full model until only those independently associated with hospital mortality remained.

Abbreviations: **AOR**, adjusted odds ratio; **ARDS**, acute respiratory distress syndrome; **CCI**, Charlson Comorbidity Index; **CI**, confidence interval; **HAP**, hospital-acquired pneumonia; **NA**, not available; **NMBAs**, continuous neuromuscular blocking agents; **OR**, odds ratio; **RR**, respiration rate; **SOFA**, Sequential Organ Failure Assessment.

Supplementary Table 23. Multivariable Regression Analysis of SOFA Score ≥Cut-off Value and Hospital Mortality in Critically Ill COVID-19 Patients with Delta Variant upon Admission, Adjusted for Baseline and Mediator Confounders

Factors	Univariable logistic regression analyses[a]				Multivariable logistic regression analysis[b]			
	OR	95% CI for OR		p-value	AOR	95% CI for AOR		p-value
		Lower	Upper			Lower	Upper	
**Exposure variable**								
Admission severity of illness								
SOFA score ≥ 3.5[c]	6.667	1.707	26.042	0.006	NA	NA	NA	NA
**Confounding variables**								

Inter-hospital transfers								
Endotracheal tube	2.180	0.454	10.645	0.330	NA	NA	NA	NA
Nasal cannula	0.077	0.008	0.783	0.030	NA	NA	NA	NA
Demographics								
Age (year)	1.039	1.005	1.074	0.023	NA	NA	NA	NA
Gender (male)	2.043	0.726	5.743	0.176	NA	NA	NA	NA
Comorbidities								
CCI score	1.401	1.027	1.912	0.033	NA	NA	NA	NA
Admission vital signs								
RR (breaths/min)	1.191	1.046	1.357	0.008	1.214	0.979	1.505	0.077
Admission labs								
Fibrinogen (g/L)	1.592	1.137	2.228	0.007	NA	NA	NA	NA
1st-day respiratory support								
Bag valve mask	0.375	0.141	0.999	0.050	NA	NA	NA	NA
High-flow nasal cannula	2.523	0.838	7.598	0.100	NA	NA	NA	NA
Adjunctive therapies								
Antiviral drugs	0.437	0.147	1.300	0.136	NA	NA	NA	NA
NMBAs	8.421	2.870	24.707	<0.001	14.179	2.441	82.365	0.003
Renal replacement therapy	5.815	0.732	46.220	0.096	NA	NA	NA	NA
Complications								
HAP	18.353	1.928	174.691	0.011	NA	NA	NA	NA
ARDS	6.333	1.290	31.090	0.023	NA	NA	NA	NA
Constant					0.009			0.089

[a)] Each exposure and confounding variable was first analysed using a univariable logistic regression model. Variables with a P-value ≤0.10 in the univariable analysis and clinically significant factors were included in the multivariable logistic regression model. To ensure the robustness of our model, we evaluated multicollinearity among the predictor variables using the variance inflation factor (VIF) analysis. All VIF values were within acceptable limits, indicating no significant collinearity concerns. In addition, the final model demonstrated a good model fit, with -2 Log-Likelihood (-2LL) of 36.724 and a pseudo R^2 of 0.434, indicating strong explanatory power. The Hosmer-Lemeshow goodness-of-fit test yielded a χ^2 2 (8)=8.409, p-value = 0.395, suggesting excellent calibration and no evidence of poor fit.

[b)] The multivariable logistic regression model utilized a stepwise backward elimination approach. Variables were systematically removed from the full model until only those independently associated with hospital mortality remained.
[c)] The numbers represent the best cut-off value determined by analysing each variable's receiver operator characteristic curve.
Abbreviations: **AOR**, adjusted odds ratio; **ARDS**, acute respiratory distress syndrome; **CCI**, Charlson Comorbidity Index; **CI**, confidence interval; **HAP**, hospital-acquired pneumonia; **NA**, not available; **NMBAs**, continuous neuromuscular blocking agents; **OR**, odds ratio; **RR**, respiration rate; **SOFA**, Sequential Organ Failure Assessment.

Supplementary Table 24. Multivariable Regression Analysis of APACHE II Score and Hospital Mortality in Critically Ill COVID-19 Patients with Delta Variant upon Admission, Adjusted for Baseline and Mediator Confounders

Factors	Univariable logistic regression analyses[a]				Multivariable logistic regression analysis[b]			
	OR	95% CI for OR		p-value	AOR	95% CI for AOR		p-value
		Lower	Upper			Lower	Upper	
**Exposure variable**								
Admission severity of illness								
APACHE II score	1.117	0.988	1.261	0.077	NA	NA	NA	NA
**Confounding variables**								
Inter-hospital transfers								
Endotracheal tube	2.180	0.454	10.645	0.330	NA	NA	NA	NA
Nasal cannula	0.077	0.008	0.783	0.030	NA	NA	NA	NA
Demographics								
Age (year)	1.039	1.005	1.074	0.023	NA	NA	NA	NA
Gender (male)	2.043	0.726	5.743	0.176	NA	NA	NA	NA
Comorbidities								
CCI score	1.401	1.027	1.912	0.033	NA	NA	NA	NA
Admission vital signs								
RR (breaths/min)	1.191	1.046	1.357	0.008	1.176	0972	1.422	0.096
Admission labs								

Fibrinogen (g/L)	1.592	1.137	2.228	0.007	NA	NA	NA	NA
1st-day respiratory support								
Bag valve mask	0.375	0.141	0.999	0.050	NA	NA	NA	NA
High-flow nasal cannula	2.523	0.838	7.598	0.100	NA	NA	NA	NA
Adjunctive therapies								
Antiviral drugs	0.437	0.147	1.300	0.136	NA	NA	NA	NA
NMBAs	8.421	2.870	24.707	<0.001	9.099	1.928	42.946	0.005
Renal replacement therapy	5.815	0.732	46.220	0.096	NA	NA	NA	NA
Complications								
HAP	18.353	1.928	174.691	0.011	NA	NA	NA	NA
ARDS	6.333	1.290	31.090	0.023	NA	NA	NA	NA
Constant					0.019			0.115

[a] Each exposure and confounding variable was first analysed using a univariable logistic regression model. Variables with a P-value ≤0.10 in the univariable analysis and clinically significant factors were included in the multivariable logistic regression model. To ensure the robustness of our model, we evaluated multicollinearity among the predictor variables using the variance inflation factor (VIF) analysis. All VIF values were within acceptable limits, indicating no significant collinearity concerns. In addition, the final model demonstrated a good model fit, with -2 Log-Likelihood (-2LL) of 42.774 and a pseudo R^2 of 0.347, indicating strong explanatory power. The Hosmer-Lemeshow goodness-of-fit test yielded a χ^2 2 (8)=8.032, p-value = 0.330, suggesting excellent calibration and no evidence of poor fit.

[b] The multivariable logistic regression model utilized a stepwise backward elimination approach. Variables were systematically removed from the full model until only those independently associated with hospital mortality remained.

Abbreviations: **AOR**, adjusted odds ratio; **APACHE II**, Acute Physiology and Chronic Health Evaluation II; **ARDS**, acute respiratory distress syndrome; **CCI**, Charlson Comorbidity Index; **CI**, confidence interval; **HAP**, hospital-acquired pneumonia; **NA**, not available; **NMBAs**, continuous neuromuscular blocking agents; **OR**, odds ratio; **RR**, respiration rate.

Supplementary Table 25. Multivariable Regression Analysis of APACHE II Score ≥Cut-off Value and Hospital Mortality in Critically Ill COVID-19 Patients with Delta Variant upon Admission, Adjusted for Baseline and Mediator Confounders

Factors	Univariable logistic regression analyses[a]				Multivariable logistic regression analysis[b]			
	OR	95% CI for OR		p-value	AOR	95% CI for AOR		p-value
		Lower	Upper			Lower	Upper	
**Exposure variable**								
Admission severity of illness								
APACHE II score ≥ 11.5[c]	7.692	0.947	62.490	0.056	NA	NA	NA	NA
**Confounding variables**								
Inter-hospital transfers								
Endotracheal tube	2.180	0.454	10.645	0.330	NA	NA	NA	NA
Nasal cannula	0.077	0.008	0.783	0.030	NA	NA	NA	NA
Demographics								
Age (year)	1.039	1.005	1.074	0.023	NA	NA	NA	NA
Gender (male)	2.043	0.726	5.743	0.176	NA	NA	NA	NA
Comorbidities								
CCI score	1.401	1.027	1.912	0.033	NA	NA	NA	NA
Admission vital signs								
RR (breaths/min)	1.191	1.046	1.357	0.008	1.176	0.972	1.422	0.096
Admission labs								
Fibrinogen (g/L)	1.592	1.137	2.228	0.007	NA	NA	NA	NA
1st-day respiratory support								
Bag valve mask	0.375	0.141	0.999	0.050	NA	NA	NA	NA
High-flow nasal cannula	2.523	0.838	7.598	0.100	NA	NA	NA	NA
Adjunctive therapies								
Antiviral drugs	0.437	0.147	1.300	0.136	NA	NA	NA	NA
NMBAs	8.421	2.870	24.707	<0.001	9.099	1.928	42.946	0.005

Renal replacement therapy	5.815	0.732	46.220	0.096	NA	NA	NA	NA
Complications								
HAP	18.353	1.928	174.691	0.011	NA	NA	NA	NA
ARDS	6.333	1.290	31.090	0.023	NA	NA	NA	NA
Constant					0.019			0.115

[a)] Each exposure and confounding variable was first analysed using a univariable logistic regression model. Variables with a P-value ≤0.10 in the univariable analysis and clinically significant factors were included in the multivariable logistic regression model. To ensure the robustness of our model, we evaluated multicollinearity among the predictor variables using the variance inflation factor (VIF) analysis. All VIF values were within acceptable limits, indicating no significant collinearity concerns. In addition, the final model demonstrated a good model fit, with -2 Log-Likelihood (-2LL) of 42.774 and a pseudo R^2 of 0.347, indicating strong explanatory power. The Hosmer-Lemeshow goodness-of-fit test yielded a χ^2 2 (8)=8.032, p-value = 0.330, suggesting excellent calibration and no evidence of poor fit.

[b)] The multivariable logistic regression model utilized a stepwise backward elimination approach. Variables were systematically removed from the full model until only those independently associated with hospital mortality remained.

[c)] The numbers represent the best cut-off value determined by analysing each variable's receiver operator characteristic curve.

Abbreviations: **AOR**, adjusted odds ratio; **APACHE II**, Acute Physiology and Chronic Health Evaluation II; **ARDS**, acute respiratory distress syndrome; **CCI**, Charlson Comorbidity Index; **CI**, confidence interval; **HAP**, hospital-acquired pneumonia; **NA**, not available; **NMBAs**, continuous neuromuscular blocking agents; **OR**, odds ratio; **RR**, respiration rate.

Supplementary Table 26. Multivariable Regression Analysis of CURB-65 Score and Hospital Mortality in Critically Ill COVID-19 Patients with Delta Variant upon Admission, Adjusted for Baseline and Mediator Confounders

Factors	Univariable logistic regression analyses				Multivariable logistic regression analysis			
	OR	95% CI for OR		p-value	AOR	95% CI for AOR		p-value
		Lower	Upper			Lower	Upper	
**Exposure variable**								
Admission severity of illness								
CURB-65 score	4.436	1.940	10.146	<0.001	NA	NA	NA	NA
**Confounding variables**								
Inter-hospital transfers								
Endotracheal tube	2.180	0.454	10.645	0.330	NA	NA	NA	NA

Nasal cannula	0.077	0.008	0.783	0.030	NA	NA	NA	NA
Demographics								
Age (year)	1.039	1.005	1.074	0.023	NA	NA	NA	NA
Gender (male)	2.043	0.726	5.743	0.176	NA	NA	NA	NA
Comorbidities								
CCI score	1.401	1.027	1.912	0.033	NA	NA	NA	NA
Admission vital signs								
RR (breaths/min)	1.191	1.046	1.357	0.008	1.185	1.010	1.391	0.038
Admission labs								
Fibrinogen (g/L)	1.592	1.137	2.228	0.007	1.519	0.955	2.415	0.078
1st-day respiratory support								
Bag valve mask	0.375	0.141	0.999	0.050	NA	NA	NA	NA
High-flow nasal cannula	2.523	0.838	7.598	0.100	NA	NA	NA	NA
Adjunctive therapies								
Antiviral drugs	0.437	0.147	1.300	0.136	NA	NA	NA	NA
NMBAs	8.421	2.870	24.707	<0.001	7.191	1.784	28.985	0.006
Renal replacement therapy	5.815	0.732	46.220	0.096	NA	NA	NA	NA
Complications								
HAP	18.353	1.928	174.691	0.011	NA	NA	NA	NA
ARDS	6.333	1.290	31.090	0.023	NA	NA	NA	NA
Constant					0.002			0.011

[a)] Each exposure and confounding variable was first analysed using a univariable logistic regression model. Variables with a P-value ≤0.10 in the univariable analysis and clinically significant factors were included in the multivariable logistic regression model. To ensure the robustness of our model, we evaluated multicollinearity among the predictor variables using the variance inflation factor (VIF) analysis. All VIF values were within acceptable limits, indicating no significant collinearity concerns. In addition, the final model demonstrated a good model fit, with -2 Log-Likelihood (-2LL) of 52.158 and a pseudo R^2 of 0.375, indicating strong explanatory power. The Hosmer-Lemeshow goodness-of-fit test yielded a χ^2 2 (8)=8.000 p-value = 0.433 suggesting excellent calibration and no evidence of poor fit.

[b)] The multivariable logistic regression model utilized a stepwise backward elimination approach. Variables were systematically removed from the full model until only those independently associated with hospital mortality remained.

Abbreviations: **AOR**, adjusted odds ratio; **ARDS**, acute respiratory distress syndrome; **CCI**, Charlson Comorbidity Index; **CI**, confidence interval; **CURB-65**, Confusion,

Urea >7 mmol/L, Respiratory Rate ≥30 breaths/min, Blood Pressure <90 mm Hg (Systolic) or <60 mm Hg (Diastolic), Age ≥65 Years; **HAP**, hospital-acquired pneumonia; **NA**, not available; **NMBAs**, continuous neuromuscular blocking agents; **OR**, odds ratio; **RR**, respiration rate.

Supplementary Table 27. Multivariable Regression Analysis of CURB-65 Score ≥Cut-off Value and Hospital Mortality in Critically Ill COVID-19 Patients with Delta Variant upon Admission, Adjusted for Baseline and Mediator Confounders

Factors	Univariable logistic regression analyses[a]				Multivariable logistic regression analysis[b]			
	OR	95% CI for OR		p-value	AOR	95% CI for AOR		p-value
		Lower	Upper			Lower	Upper	
**Exposure variable**								
Admission severity of illness								
CURB-65 score ≥ 0.5[c]	8.875	2.835	27.786	<0.001	NA	NA	NA	NA
**Confounding variables**								
Inter-hospital transfers								
Endotracheal tube	2.180	0.454	10.645	0.330	NA	NA	NA	NA
Nasal cannula	0.077	0.008	0.783	0.030	NA	NA	NA	NA
Demographics								
Age (year)	1.039	1.005	1.074	0.023	NA	NA	NA	NA
Gender (male)	2.043	0.726	5.743	0.176	NA	NA	NA	NA
Comorbidities								
CCI score	1.401	1.027	1.912	0.033	NA	NA	NA	NA
Admission vital signs								
RR (breaths/min)	1.191	1.046	1.357	0.008	1.185	1.010	1.391	0.038
Admission labs								
Fibrinogen (g/L)	1.592	1.137	2.228	0.007	1.519	0.955	2.415	0.078
1st-day respiratory support								
Bag valve mask	0.375	0.141	0.999	0.050	NA	NA	NA	NA

High-flow nasal cannula	2.523	0.838	7.598	0.100	NA	NA	NA	NA
Adjunctive therapies								
Antiviral drugs	0.437	0.147	1.300	0.136	NA	NA	NA	NA
NMBAs	8.421	2.870	24.707	<0.001	7.191	1.784	28.985	0.006
Renal replacement therapy	5.815	0.732	46.220	0.096	NA	NA	NA	NA
Complications								
HAP	18.353	1.928	174.691	0.011	NA	NA	NA	NA
ARDS	6.333	1.290	31.090	0.023	NA	NA	NA	NA
Constant					0.002			0.011

[a)] Each exposure and confounding variable was first analysed using a univariable logistic regression model. Variables with a P-value ≤0.10 in the univariable analysis and clinically significant factors were included in the multivariable logistic regression model. To ensure the robustness of our model, we evaluated multicollinearity among the predictor variables using the variance inflation factor (VIF) analysis. All VIF values were within acceptable limits, indicating no significant collinearity concerns. In addition, the final model demonstrated a good model fit, with -2 Log-Likelihood (-2LL) of 52.158 and a pseudo R^2 of 0.375, indicating strong explanatory power. The Hosmer-Lemeshow goodness-of-fit test yielded a χ^2 2 (8)=8.000 p-value = 0.433 suggesting excellent calibration and no evidence of poor fit.

[b)] The multivariable logistic regression model utilized a stepwise backward elimination approach. Variables were systematically removed from the full model until only those independently associated with hospital mortality remained.

[c)] The numbers represent the best cut-off value determined by analysing each variable's receiver operator characteristic curve.

Abbreviations: **AOR**, adjusted odds ratio; **ARDS**, acute respiratory distress syndrome; **CCI**, Charlson Comorbidity Index; **CI**, confidence interval; **CURB-65**, Confusion, Urea >7 mmol/L, Respiratory Rate ≥30 breaths/min, Blood Pressure <90 mm Hg (Systolic) or <60 mm Hg (Diastolic), Age ≥65 Years; **HAP**, hospital-acquired pneumonia; **NA**, not available; **NMBAs**, continuous neuromuscular blocking agents; **OR**, odds ratio; **RR**, respiration rate.

Supplementary Table 28. Multivariable Regression Analysis of First-day PaO_2/FiO_2 Ratio and Hospital Mortality in Critically Ill COVID-19 Patients with Delta Variant upon Admission, Adjusted for Baseline and Mediator Confounders

Factors	Univariable logistic regression analyses				Multivariable logistic regression analysis			
	OR	95% CI for OR		p-value	AOR	95% CI for AOR		p-value
		Lower	Upper			Lower	Upper	
**Exposure variable**								
The 1st-day gas exchange								

PaO_2/FiO_2 ratio	0.993	0.983	1.002	0.140	NA	NA	NA	NA
**Confounding variables**								
Inter-hospital transfers								
Endotracheal tube	2.180	0.454	10.645	0.330	NA	NA	NA	NA
Nasal cannula	0.077	0.008	0.783	0.030	NA	NA	NA	NA
Demographics								
Age (year)	1.039	1.005	1.074	0.023	NA	NA	NA	NA
Gender (male)	2.043	0.726	5.743	0.176	NA	NA	NA	NA
Comorbidities								
CCI score	1.401	1.027	1.912	0.033	NA	NA	NA	NA
Admission vital signs								
RR (breaths/min)	1.191	1.046	1.357	0.008	1.180	0.953	1.461	0.129
Admission labs								
Fibrinogen (g/L)	1.592	1.137	2.228	0.007	NA	NA	NA	NA
1st-day respiratory support								
Bag valve mask	0.375	0.141	0.999	0.050	NA	NA	NA	NA
High-flow nasal cannula	2.523	0.838	7.598	0.100	NA	NA	NA	NA
Adjunctive therapies								
Antiviral drugs	0.437	0.147	1.300	0.136	NA	NA	NA	NA
NMBAs	8.421	2.870	24.707	<0.001	16.586	2.530	108.726	0.003
Renal replacement therapy	5.815	0.732	46.220	0.096	NA	NA	NA	NA
Complications								
HAP	18.353	1.928	174.691	0.011	NA	NA	NA	NA
ARDS	6.333	1.290	31.090	0.023	NA	NA	NA	NA
Constant					0.014			0.142

a) Each exposure and confounding variable was first analysed using a univariable logistic regression model. Variables with a P-value ≤0.10 in the univariable analysis and clinically significant factors were included in the multivariable logistic regression model. To ensure the robustness of our model, we evaluated multicollinearity among the predictor variables using the variance inflation factor (VIF) analysis. All VIF values were within acceptable limits, indicating no significant collinearity concerns. In addition, the final model demonstrated a good model fit, with -2 Log-Likelihood (-2LL) of 30.542 and a pseudo R^2 of 0.403, indicating strong explanatory power. The Hosmer-Lemeshow goodness-of-fit test yielded a χ^2 2 (8)=5.047, p-value = 0.538, suggesting excellent calibration and no evidence of poor fit.

b) The multivariable logistic regression model utilized a stepwise backward elimination approach. Variables were systematically removed from the full model until only those independently associated with hospital mortality remained.

Abbreviations: **AOR**, adjusted odds ratio; **ARDS**, acute respiratory distress syndrome; **CCI**, Charlson Comorbidity Index; **CI**, confidence interval; **HAP**, hospital-acquired pneumonia; **NA**, not available; **NMBAs**, continuous neuromuscular blocking agents; **OR**, odds ratio; **PaO_2/FiO_2**, arterial oxygen partial pressure to inspired oxygen fraction ratio; **RR**, respiration rate.

Supplementary Table 29. Multivariable Regression Analysis of First-day PaO_2/FiO_2 Ratio ≥Cut-off Value and Hospital Mortality in Critically Ill COVID-19 Patients with Delta Variant upon Admission, Adjusted for Baseline and Mediator Confounders

Factors	Univariable logistic regression analyses[a]				Multivariable logistic regression analysis[b]			
	OR	95% CI for OR		p-value	AOR	95% CI for AOR		p-value
		Lower	Upper			Lower	Upper	
**Exposure variable**								
The 1st-day Laboratory investigations								
$PaO_2/FiO_2 \geq 119.5$ mmHg[c]	0.179	0.045	0.716	0.015	NA	NA	NA	NA
**Confounding variables**								
Inter-hospital transfers								
Endotracheal tube	2.180	0.454	10.645	0.330	NA	NA	NA	NA
Nasal cannula	0.077	0.008	0.783	0.030	NA	NA	NA	NA
Demographics								
Age (year)	1.039	1.005	1.074	0.023	NA	NA	NA	NA
Gender (male)	2.043	0.726	5.743	0.176	NA	NA	NA	NA

Comorbidities								
CCI score	1.401	1.027	1.912	0.033	NA	NA	NA	NA
Admission vital signs								
RR (breaths/min)	1.191	1.046	1.357	0.008	1.180	0.953	1.461	0.129
Admission labs								
Fibrinogen (g/L)	1.592	1.137	2.228	0.007	NA	NA	NA	NA
1st-day respiratory support								
Bag valve mask	0.375	0.141	0.999	0.050	NA	NA	NA	NA
High-flow nasal cannula	2.523	0.838	7.598	0.100	NA	NA	NA	NA
Adjunctive therapies								
Antiviral drugs	0.437	0.147	1.300	0.136	NA	NA	NA	NA
NMBAs	8.421	2.870	24.707	<0.001	16.586	2.530	108.726	0.003
Renal replacement therapy	5.815	0.732	46.220	0.096	NA	NA	NA	NA
Complications								
HAP	18.353	1.928	174.691	0.011	NA	NA	NA	NA
ARDS	6.333	1.290	31.090	0.023	NA	NA	NA	NA
Constant					0.014			0.142

[a)] Each exposure and confounding variable was first analysed using a univariable logistic regression model. Variables with a P-value ≤0.10 in the univariable analysis and clinically significant factors were included in the multivariable logistic regression model. To ensure the robustness of our model, we evaluated multicollinearity among the predictor variables using the variance inflation factor (VIF) analysis. All VIF values were within acceptable limits, indicating no significant collinearity concerns. In addition, the final model demonstrated a good model fit, with -2 Log-Likelihood (-2LL) of 30.542 and a pseudo R^2 of 0.403, indicating strong explanatory power. The Hosmer-Lemeshow goodness-of-fit test yielded a χ^2 2 (8)=5.047, p-value = 0.538, suggesting excellent calibration and no evidence of poor fit.

[b)] The multivariable logistic regression model utilized a stepwise backward elimination approach. Variables were systematically removed from the full model until only those independently associated with hospital mortality remained.

[c)] The numbers represent the best cut-off value determined by analysing each variable's receiver operator characteristic curve.

Abbreviations: **AOR**, adjusted odds ratio; **ARDS**, acute respiratory distress syndrome; **CCI**, Charlson Comorbidity Index; **CI**, confidence interval; **HAP**, hospital-acquired pneumonia; **NA**, not available; **NMBAs**, continuous neuromuscular blocking agents; **OR**, odds ratio; **PaO_2/FiO_2**, arterial oxygen partial pressure to inspired oxygen fraction ratio; **RR**, respiration rate.

Supplementary Table 30. Multivariable Regression Analysis of IL-6 Level and Hospital Mortality in Critically Ill COVID-19 Patients with Delta Variant upon Admission, Adjusted for Baseline and Mediator Confounders

Factors	Univariable logistic regression analyses				Multivariable logistic regression analysis			
	OR	95% CI for OR		p-value	AOR	95% CI for AOR		p-value
		Lower	Upper			Lower	Upper	
**Exposure variable**								
The 1st-day Laboratory investigations								
IL-6 level	1.002	0.997	1.007	0.334	NA	NA	NA	NA
**Confounding variables**								
Inter-hospital transfers								
Endotracheal tube	2.180	0.454	10.645	0.330	NA	NA	NA	NA
Nasal cannula	0.077	0.008	0.783	0.030	NA	NA	NA	NA
Demographics								
Age (year)	1.039	1.005	1.074	0.023	NA	NA	NA	NA
Gender (male)	2.043	0.726	5.743	0.176	NA	NA	NA	NA
Comorbidities								
CCI score	1.401	1.027	1.912	0.033	NA	NA	NA	NA
Admission vital signs								
RR (breaths/min)	1.191	1.046	1.357	0.008	NA	NA	NA	NA
Admission labs								
Fibrinogen (g/L)	1.592	1.137	2.228	0.007	1.583	1.027	2.441	0.037
1st-day respiratory support								
Bag valve mask	0.375	0.141	0.999	0.050	NA	NA	NA	NA
High-flow nasal cannula	2.523	0.838	7.598	0.100	NA	NA	NA	NA
Adjunctive therapies								
Antiviral drugs	0.437	0.147	1.300	0.136	NA	NA	NA	NA

NMBAs	8.421	2.870	24.707	<0.001	6.737	1.687	26.902	0.037
Renal replacement therapy	5.815	0.732	46.220	0.096	NA	NA	NA	NA
Complications								
HAP	18.353	1.928	174.691	0.011	NA	NA	NA	NA
ARDS	6.333	1.290	31.090	0.023	NA	NA	NA	NA
Constant					0.135			0.096

[a)] Each exposure and confounding variable was first analysed using a univariable logistic regression model. Variables with a P-value ≤0.10 in the univariable analysis and clinically significant factors were included in the multivariable logistic regression model. To ensure the robustness of our model, we evaluated multicollinearity among the predictor variables using the variance inflation factor (VIF) analysis. All VIF values were within acceptable limits, indicating no significant collinearity concerns. In addition, the final model demonstrated a good model fit, with -2 Log-Likelihood (-2LL) of 52.907 and a pseudo R^2 of 0.276, indicating strong explanatory power. The Hosmer-Lemeshow goodness-of-fit test yielded a χ^2 2 (8)=6.435 p-value = 0.490 suggesting excellent calibration and no evidence of poor fit.

[b)] The multivariable logistic regression model utilized a stepwise backward elimination approach. Variables were systematically removed from the full model until only those independently associated with hospital mortality remained.

Abbreviations: **AOR**, adjusted odds ratio; **ARDS**, acute respiratory distress syndrome; **CCI**, Charlson Comorbidity Index; **CI**, confidence interval; **HAP**, hospital-acquired pneumonia; **IL-6**, interleukin 6; **NA**, not available; **NMBAs**, continuous neuromuscular blocking agents; **OR**, odds ratio; **RR**, respiration rate.

Supplementary Table 31. Multivariable Regression Analysis of IL-6 Level ≥Cut-off Value and Hospital Mortality in Critically Ill COVID-19 Patients with Delta Variant upon Admission, Adjusted for Baseline and Mediator Confounders

Factors	Univariable logistic regression analyses				Multivariable logistic regression analysis			
	OR	95% CI for OR		p-value	AOR	95% CI for AOR		p-value
		Lower	Upper			Lower	Upper	
**Exposure variable**								
The 1st-day Laboratory investigations								
IL-6 level ≥ 15.8 pg/mL[c]	3.955	1.345	11.623	0.012	NA	NA	NA	NA
**Confounding variables**								
Inter-hospital transfers								

Endotracheal tube	2.180	0.454	10.645	0.330	NA	NA	NA	NA
Nasal cannula	0.077	0.008	0.783	0.030	NA	NA	NA	NA
Demographics								
Age (year)	1.039	1.005	1.074	0.023	NA	NA	NA	NA
Gender (male)	2.043	0.726	5.743	0.176	NA	NA	NA	NA
Comorbidities								
CCI score	1.401	1.027	1.912	0.033	NA	NA	NA	NA
Admission vital signs								
RR (breaths/min)	1.191	1.046	1.357	0.008	NA	NA	NA	NA
Admission labs								
Fibrinogen (g/L)	1.592	1.137	2.228	0.007	1.583	1.027	2.441	0.037
1st-day respiratory support								
Bag valve mask	0.375	0.141	0.999	0.050	NA	NA	NA	NA
High-flow nasal cannula	2.523	0.838	7.598	0.100	NA	NA	NA	NA
Adjunctive therapies								
Antiviral drugs	0.437	0.147	1.300	0.136	NA	NA	NA	NA
NMBAs	8.421	2.870	24.707	<0.001	6.737	1.687	26.902	0.037
Renal replacement therapy	5.815	0.732	46.220	0.096	NA	NA	NA	NA
Complications								
HAP	18.353	1.928	174.691	0.011	NA	NA	NA	NA
ARDS	6.333	1.290	31.090	0.023	NA	NA	NA	NA
Constant					0.135			0.096

[a] Each exposure and confounding variable was first analysed using a univariable logistic regression model. Variables with a P-value ≤0.10 in the univariable analysis and clinically significant factors were included in the multivariable logistic regression model. To ensure the robustness of our model, we evaluated multicollinearity among the predictor variables using the variance inflation factor (VIF) analysis. All VIF values were within acceptable limits, indicating no significant collinearity concerns. In addition, the final model demonstrated a good model fit, with -2 Log-Likelihood (-2LL) of 52.907 and a pseudo R^2 of 0.276, indicating strong explanatory power. The Hosmer-Lemeshow goodness-of-fit test yielded a χ^2 2 (8)=6.435 p-value = 0.490 suggesting excellent calibration and no evidence of poor fit.

[b] The multivariable logistic regression model utilized a stepwise backward elimination approach. Variables were systematically removed from the full model until only those

independently associated with hospital mortality remained.

[c)] The numbers represent the best cut-off value determined by analysing each variable's receiver operator characteristic curve.

Abbreviations: **AOR**, adjusted odds ratio; **ARDS**, acute respiratory distress syndrome; **CCI**, Charlson Comorbidity Index; **CI**, confidence interval; **HAP**, hospital-acquired pneumonia; **IL-6**, interleukin 6; **NA**, not available; **NMBAs**, continuous neuromuscular blocking agents; **OR**, odds ratio; **RR**, respiration rate.

Supplementary Table 32. Complete-Case Sensitivity Analysis of the Association of Simplified RALE Score with Hospital Mortality in Critically Ill COVID-19 Patients with Delta Variant upon Admission, Adjusted for Baseline Confounders

Analysis Type	Sensitivity Analysis	Sample size (n)	OR (95% CI) for Hospital Mortality	p-value	Interpretation
**Exposure variable**					
Initial chest imaging					
Simplified RALE score	Complete-case	87	2.170 (1.353-3.482)	0.001	Consistent with primary
**Confounding variables**					
Inter-hospital transfers					
Endotracheal tube	Complete-case	87	NA	NA	Consistent with primary
Nasal cannula	Complete-case	87	NA	NA	Consistent with primary
Demographics					
Age (year)	Complete-case	87	NA	NA	Consistent with primary
Gender (male)	Complete-case	87	10.967 (1.857-64.757)	0.008	Consistent with primary
Comorbidities					
CCI score	Complete-case	87	1.564 (1.030-2.374)	0.036	Consistent with primary
Admission vital signs					
RR (breaths/min)	Complete-case	87	1.303 (1.076-1.577)	0.007	Consistent with primary
Admission labs					
Fibrinogen (g/L)	Complete-case	87	NA	NA	Consistent with primary

Abbreviations: **AOR**, adjusted odds ratio; **CCI**, Charlson Comorbidity Index; **CI**, confidence interval; **NA**, not available; **OR**, odds ratio; **RALE**, Radiographic Assessment of Lung Edema; **RR**, respiration rate.

Supplementary Table 33. Complete-Case Sensitivity Analysis of the Association of Simplified RALE Score Dichotomized at Cut-off with Hospital Mortality in Critically Ill COVID-19 Patients with Delta Variant upon Admission, Adjusted for Baseline Confounders

Analysis Type	Sensitivity Analysis	Sample size (n)	OR (95% CI) for Hospital Mortality	p-value	Interpretation
**Exposure variable**					
Initial chest imaging					
Simplified RALE ≥5.5[a]	Complete-case	87	12.977 (2.562-65.719)	0.002	Consistent with primary
**Confounding variables**					
Inter-hospital transfers					
Endotracheal tube	Complete-case	87	NA	NA	Consistent with primary
Nasal cannula	Complete-case	87	NA	NA	Consistent with primary
Demographics					
Age (year)	Complete-case	87	NA	NA	Consistent with primary
Gender (male)	Complete-case	87	12.126 (2.045-71.909)	0.006	Consistent with primary
Comorbidities					
CCI score	Complete-case	87	1.522 (1.011-2.292)	0.044	Consistent with primary
Admission vital signs					
RR (breaths/min)	Complete-case	87	1.292 (1.074-1.553)	0.007	Consistent with primary
Admission labs					
Fibrinogen (g/L)	Complete-case	87	NA	NA	Consistent with primary

[a)] The numbers represent the best cut-off value determined by analysing each variable's receiver operator characteristic curve.

Abbreviations: **AOR**, adjusted odds ratio; **CCI**, Charlson Comorbidity Index; **CI**, confidence interval; **NA**, not available; **OR**, odds ratio; **RALE**, Radiographic Assessment

of Lung Edema; **RR**, respiration rate

Supplementary Table 34. Complete-Case Sensitivity Analysis of the Association of SOFA Score with Hospital Mortality in Critically Ill COVID-19 Patients with Delta Variant upon Admission, Adjusted for Baseline Confounders

Analysis Type	Sensitivity Analysis	Sample size (n)	OR (95% CI) for Hospital Mortality	p-value	Interpretation
**Exposure variable**					
Admission severity of illness					
SOFA score	Complete-case	74	1.417 (0.939-2.139)	0.097	Consistent with primary
**Confounding variables**					
Inter-hospital transfers					
Endotracheal tube	Complete-case	74	NA	NA	Consistent with primary
Nasal cannula	Complete-case	74	NA	NA	Consistent with primary
Demographics					
Age (year)	Complete-case	74	NA	NA	Consistent with primary
Gender (male)	Complete-case	74	3.783 (0.869-16.463)	0.076	Consistent with primary
Comorbidities					
CCI score	Complete-case	74	NA	NA	Consistent with primary
Admission vital signs					
RR (breaths/min)	Complete-case	74	1.204 (0.988-1.466)	0.066	Consistent with primary
Admission labs					
Fibrinogen (g/L)	Complete-case	74	NA	NA	Consistent with primary

Abbreviations: **AOR**, adjusted odds ratio; **CCI**, Charlson Comorbidity Index; **CI**, confidence interval **NA**, not available; **OR**, odds ratio; **RR**, respiration rate; **SOFA**, Sequential Organ Failure Assessment.

Supplementary Table 35. Complete-Case Sensitivity Analysis of the Association of SOFA Score Dichotomized at Cut-off with Hospital Mortality in Critically Ill COVID-19 Patients with Delta Variant upon Admission, Adjusted for Baseline Confounders

Analysis Type	Sensitivity Analysis	Sample size (n)	OR (95% CI) for Hospital Mortality	p-value	Interpretation
**Exposure variable**					
Admission severity of illness					
SOFA score ≥ 3.5[a]	Complete-case	74	4.450 (0.801-24.726)	0.088	Consistent with primary
**Confounding variables**					
Inter-hospital transfers					
Endotracheal tube	Complete-case	74	NA	NA	Consistent with primary
Nasal cannula	Complete-case	74	0.000 (0.000-N/A)	0.999	Consistent with primary
Demographics					
Age (year)	Complete-case	74	NA	NA	Consistent with primary
Gender (male)	Complete-case	74	7.019 (1.182-41.684)	0.032	Consistent with primary
Comorbidities					
CCI score	Complete-case	74	1.498 (0.960-2.338)	0.075	Consistent with primary
Admission vital signs					
RR (breaths/min)	Complete-case	74	1.157 (0.954-1.403)	0.139	Consistent with primary
Admission labs					
Fibrinogen (g/L)	Complete-case	74	NA	NA	Consistent with primary

[a)] The numbers represent the best cut-off value determined by analysing each variable's receiver operator characteristic curve.

Abbreviations: **AOR**, adjusted odds ratio; **CCI**, Charlson Comorbidity Index; **CI**, confidence interval **NA**, not available; **OR**, odds ratio; **RR**, respiration rate; **SOFA**, Sequential Organ Failure Assessment.

Supplementary Table 36. Complete-Case Sensitivity Analysis of the Association of APACHE II Score with Hospital Mortality in Critically Ill COVID-19 Patients with Delta Variant upon Admission, Adjusted for Baseline Confounders

Analysis Type	Sensitivity Analysis	Sample size (n)	OR (95% CI) for Hospital Mortality	p-value	Interpretation
**Exposure variable**					
Admission severity of illness					
APACHE II score	Complete-case	69	NA	NA	Consistent with primary
**Confounding variables**					
Inter-hospital transfers					
Endotracheal tube	Complete-case	69	NA	NA	Consistent with primary
Nasal cannula	Complete-case	69	0.000 (0.000-NA)	0.999	Consistent with primary
Demographics					
Age (year)	Complete-case	69	NA	NA	Consistent with primary
Gender (male)	Complete-case	69	2.079 (1.019-25.326)	0.047	Consistent with primary
Comorbidities					
CCI score	Complete-case	69	1.424 (0.945-2.144)	0.091	Consistent with primary
Admission vital signs					
RR (breaths/min)	Complete-case	69	1.200 (1.003-1.436)	0.046	Consistent with primary
Admission labs					
Fibrinogen (g/L)	Complete-case	69	NA	NA	Consistent with primary

Abbreviations: **AOR**, adjusted odds ratio; **APACHE II**, Acute Physiology and Chronic Health Evaluation II; **CCI**, Charlson Comorbidity Index; **CI**, confidence interval; **NA**, not available; **OR**, odds ratio; **RR**, respiration rate.

Supplementary Table 37. Complete-Case Sensitivity Analysis of the Association of APACHE II Score Dichotomized at Cut-off with Hospital Mortality in Critically Ill COVID-19 Patients with Delta Variant upon Admission, Adjusted for Baseline Confounders

Analysis Type	Sensitivity Analysis	Sample size (n)	OR (95% CI) for Hospital Mortality	p-value	Interpretation
**Exposure variable**					
Admission severity of illness					
APACHE II score ≥ 11.5[a]	Complete-case	69	NA	NA	Consistent with primary
**Confounding variables**					
Inter-hospital transfers					
Endotracheal tube	Complete-case	69	NA	NA	Consistent with primary
Nasal cannula	Complete-case	69	0.000 (0.000-NA)	0.999	Consistent with primary
Demographics					
Age (year)	Complete-case	69	NA	NA	Consistent with primary
Gender (male)	Complete-case	69	5.079 (1.019-25.326)	0.047	Consistent with primary
Comorbidities					
CCI score	Complete-case	69	1.424 (0.945-2.144)	0.091	Consistent with primary
Admission vital signs					
RR (breaths/min)	Complete-case	69	1.200 (1.003-1.436)	0.046	Consistent with primary
Admission labs					
Fibrinogen (g/L)	Complete-case	69	NA	NA	Consistent with primary

[a)] The numbers represent the best cut-off value determined by analysing each variable's receiver operator characteristic curve.

Abbreviations: **AOR**, adjusted odds ratio; **APACHE II**, Acute Physiology and Chronic Health Evaluation II; **CCI**, Charlson Comorbidity Index; **CI**, confidence interval; **NA**, not available; **OR**, odds ratio; **RR**, respiration rate.

Supplementary Table 38. Complete-Case Sensitivity Analysis of the Association of CURB-65 Score with Hospital Mortality in Critically Ill COVID-19 Patients with Delta Variant upon Admission, Adjusted for Baseline Confounders

Analysis Type	Sensitivity Analysis	Sample size (n)	OR (95% CI) for Hospital Mortality	p-value	Interpretation
**Exposure variable**					
Admission severity of illness					
CURB-65 score	Complete-case	86	4.316 (1.684-11.059)	0.002	Consistent with primary
**Confounding variables**					
Inter-hospital transfers					
Endotracheal tube	Complete-case	86	NA	NA	Consistent with primary
Nasal cannula	Complete-case	86	0.000 (0.000-NA)	0.999	Consistent with primary
Demographics					
Age (year)	Complete-case	86	NA	NA	Consistent with primary
Gender (male)	Complete-case	86	NA	NA	Consistent with primary
Comorbidities					
CCI score	Complete-case	86	NA	NA	Consistent with primary
Admission vital signs					
RR (breaths/min)	Complete-case	86	NA	NA	Consistent with primary
Admission labs					
Fibrinogen (g/L)	Complete-case	86	1.640 (1.049-2.565)	0.030	Consistent with primary

Abbreviations: **AOR**, adjusted odds ratio; **CCI**, Charlson Comorbidity Index; **CI**, confidence interval; **CURB-65**, Confusion, Urea >7 mmol/L, Respiratory Rate ≥30 breaths/min, Blood Pressure <90 mm Hg (Systolic) or <60 mm Hg (Diastolic), Age ≥65 Years; **NA**, not available; **OR**, odds ratio; **RR**, respiration rate

Supplementary Table 39. Complete-Case Sensitivity Analysis of the Association of CURB-65 Score Dichotomized at Cut-off with Hospital Mortality in Critically Ill COVID-19 Patients with Delta Variant upon Admission, Adjusted for Baseline Confounders

Analysis Type	Sensitivity Analysis	Sample size (n)	OR (95% CI) for Hospital Mortality	p-value	Interpretation
**Exposure variable**					
Admission severity of illness					
CURB-65 score ≥ 0.5[a]	Complete-case	86	6.283 (1.568-25.185)	0.009	Consistent with primary
**Confounding variables**					
Inter-hospital transfers					
Endotracheal tube	Complete-case	86	NA	NA	Consistent with primary
Nasal cannula	Complete-case	86	NA	NA	Consistent with primary
Demographics					
Age (year)	Complete-case	86	NA	NA	Consistent with primary
Gender (male)	Complete-case	86	4.137 (1.065-16.063)	0.040	Consistent with primary
Comorbidities					
CCI score	Complete-case	86	NA	NA	Consistent with primary
Admission vital signs					
RR (breaths/min)	Complete-case	86	1.168 (0.986-1.385)	0.073	Consistent with primary
Admission labs					
Fibrinogen (g/L)	Complete-case	86	NA	NA	Consistent with primary

[a)] The numbers represent the best cut-off value determined by analysing each variable's receiver operator characteristic curve.

Abbreviations: **AOR**, adjusted odds ratio; **CCI**, Charlson Comorbidity Index; **CI**, confidence interval; **CURB-65**, Confusion, Urea >7 mmol/L, Respiratory Rate ≥30 breaths/min, Blood Pressure <90 mm Hg (Systolic) or <60 mm Hg (Diastolic), Age ≥65 Years; **NA**, not available; **OR**, odds ratio; **RR**, respiration rate

Supplementary Table 40. Complete-Case Sensitivity Analysis of the Association of First-day PaO_2/FiO_2 Ratio with Hospital Mortality in Critically Ill COVID-19 Patients with Delta Variant upon Admission, Adjusted for Baseline Confounders

Analysis Type	Sensitivity Analysis	Sample size (n)	OR (95% CI) for Hospital Mortality	p-value	Interpretation
**Exposure variable**					
The 1st-day gas exchange					
PaO_2/FiO_2 ratio	Complete-case	57	NA	NA	Consistent with primary
**Confounding variables**					
Inter-hospital transfers					
Endotracheal tube	Complete-case	57	NA	NA	Consistent with primary
Nasal cannula	Complete-case	57	NA	NA	Consistent with primary
Demographics					
Age (year)	Complete-case	57	NA	NA	Consistent with primary
Gender (male)	Complete-case	57	4.505 (0.805-25.199)	0.087	Consistent with primary
Comorbidities					
CCI score	Complete-case	57	NA	NA	Consistent with primary
Admission vital signs					
RR (breaths/min)	Complete-case	57	1.172 (0.977-1.405)	0.088	Consistent with primary
Admission labs					
Fibrinogen (g/L)	Complete-case	57	NA	NA	Consistent with primary

Abbreviations: **AOR**, adjusted odds ratio; **CCI**, Charlson Comorbidity Index; **CI**, confidence interval; **NA**, not available; **OR**, odds ratio; **PaO_2/FiO_2**, arterial oxygen partial pressure to inspired oxygen fraction ratio; **RR**, respiration rate.

Supplementary Table 41. Complete-Case Sensitivity Analysis of the Association of First-day PaO_2/FiO_2 Ratio Dichotomized at Cut-off with Hospital Mortality in Critically Ill COVID-19 Patients with Delta Variant upon Admission, Adjusted for Baseline Confounders

Analysis Type	Sensitivity Analysis	Sample size (n)	OR (95% CI) for Hospital Mortality	p-value	Interpretation
**Exposure variable**					
The 1st-day gas exchange					
$PaO_2/FiO_2 \geq 119.5$ mmHg[a]	Complete-case	57	0.166 (0.037-0.736)	0.018	Consistent with primary
**Confounding variables**					
Inter-hospital transfers					
Endotracheal tube	Complete-case	57	NA	NA	Consistent with primary
Nasal cannula	Complete-case	57	NA	NA	Consistent with primary
Demographics					
Age (year)	Complete-case	57	NA	NA	Consistent with primary
Gender (male)	Complete-case	57	3.267 (0.579-18.426)	0.180	Consistent with primary
Comorbidities					
CCI score	Complete-case	57	NA	NA	Consistent with primary
Admission vital signs					
RR (breaths/min)	Complete-case	57	NA	NA	Consistent with primary
Admission labs					
Fibrinogen (g/L)	Complete-case	57	NA	NA	Consistent with primary

[a)] The numbers represent the best cut-off value determined by analysing each variable's receiver operator characteristic curve.

Abbreviations: **AOR**, adjusted odds ratio; **CCI**, Charlson Comorbidity Index; **CI**, confidence interval; **NA**, not available; **OR**, odds ratio; **PaO_2/FiO_2**, arterial oxygen partial pressure to inspired oxygen fraction ratio; **RR**, respiration rate.

Supplementary Table 42. Complete-Case Sensitivity Analysis of the Association of IL-6 Level with Hospital Mortality in Critically Ill COVID-19 Patients with Delta Variant upon Admission, Adjusted for Baseline Confounders

Analysis Type	Sensitivity Analysis	Sample size (n)	OR (95% CI) for Hospital Mortality	p-value	Interpretation
**Exposure variable**					
The 1st-day Laboratory investigations					
IL-6 level	Complete-case	78	NA	NA	Consistent with primary
**Confounding variables**					
Inter-hospital transfers					
Endotracheal tube	Complete-case	78	NA	NA	Consistent with primary
Nasal cannula	Complete-case	78	0.000 (0.000- NA)	0.999	Consistent with primary
Demographics					
Age (year)	Complete-case	78	NA	NA	Consistent with primary
Gender (male)	Complete-case	78	5.314 (1.235-22.877)	0.025	Consistent with primary
Comorbidities					
CCI score	Complete-case	78	1.579 (1.046-2.383)	0.030	Consistent with primary
Admission vital signs					
RR (breaths/min)	Complete-case	78	1.218 (1.032-1.437)	0.020	Consistent with primary
Admission labs					
Fibrinogen (g/L)	Complete-case	78	NA	NA	Consistent with primary

Abbreviations: **AOR**, adjusted odds ratio; **CCI**, Charlson Comorbidity Index; **CI**, confidence interval; **IL-6**, interleukin 6; **NA**, not available; **OR**, odds ratio; **RR**, respiration rate.

Supplementary Table 43. Complete-Case Sensitivity Analysis of the Association of IL-6 Level Dichotomized at Cut-off with Hospital Mortality in Critically Ill COVID-19 Patients with Delta Variant upon Admission, Adjusted for Baseline Confounders

Analysis Type	Sensitivity Analysis	Sample size (n)	OR (95% CI) for Hospital Mortality	p-value	Interpretation
**Exposure variable**					
The 1st-day Laboratory investigations					
IL-6 level ≥ 15.8 pg/mL[a]	Complete-case	78	NA	NA	Consistent with primary
**Confounding variables**					
Inter-hospital transfers					
Endotracheal tube	Complete-case	78	NA	NA	Consistent with primary
Nasal cannula	Complete-case	78	0.000 (0.000- NA)	0.999	Consistent with primary
Demographics					
Age (year)	Complete-case	78	NA	NA	Consistent with primary
Gender (male)	Complete-case	78	5.314 (1.235-22.877)	0.025	Consistent with primary
Comorbidities					
CCI score	Complete-case	78	1.579 (1.046-2.383)	0.030	Consistent with primary
Admission vital signs					
RR (breaths/min)	Complete-case	78	1.218 (1.032-1.437)	0.020	Consistent with primary
Admission labs					
Fibrinogen (g/L)	Complete-case	78	NA	NA	Consistent with primary

a) The numbers represent the best cut-off value determined by analysing each variable's receiver operator characteristic curve.

Abbreviations: **AOR**, adjusted odds ratio; **CCI**, Charlson Comorbidity Index; **CI**, confidence interval; **IL-6**, interleukin 6; **NA**, not available; **OR**, odds ratio; **RR**, respiration rate.

Supplementary Table 44. Complete-Case Sensitivity Analysis of the Association of Simplified RALE Score with Hospital Mortality in Critically Ill COVID-19 Patients with Delta Variant upon Admission, Adjusted for Baseline and Mediator Confounders

Analysis Type	Sensitivity Analysis	Sample size (n)	OR (95% CI) for Hospital Mortality	p-value	Interpretation
**Exposure variable**					
Initial chest imaging					
Simplified RALE score	Complete-case	76	1.870 (1.034-3.381)	0.038	Consistent with primary
**Confounding variables**					
Inter-hospital transfers					
Endotracheal tube	Complete-case	76	1.034 (0.035-30.096)	0.985	Consistent with primary
Nasal cannula	Complete-case	76	0.000 (0.000-NA)	0.999	Consistent with primary
Demographics					
Age (year)	Complete-case	76	1.026 (0.904-1.164)	0.690	Consistent with primary
Gender (male)	Complete-case	76	5.843 (0.689-49.546)	0.106	Not Significant
Comorbidities					
CCI score	Complete-case	76	1.287 (0.511-3.239)	0.592	Consistent with primary
Admission vital signs					
RR (breaths/min)	Complete-case	76	1.167 (0.929-1.466)	0.184	Not Significant
Admission labs					
Fibrinogen (g/L)	Complete-case	76	1.452 (0.823-2.563)	0.198	Consistent with primary
1st-day respiratory support					
Bag valve mask	Complete-case	76	0.688 (0.058-8.201)	0.767	Consistent with primary
High-flow nasal cannula	Complete-case	76	0.567 (0.054-5.952)	0.637	Consistent with primary
Adjunctive therapies					
Antiviral drugs	Complete-case	76	0.944 (0.094-9.508)	0.961	Consistent with primary
NMBAs	Complete-case	76	2.159 (0.301-15.467)	0.444	Consistent with primary

Renal replacement therapy	Complete-case	76	1.599 (0.099-25.864)	0.741	Consistent with primary
Complications					
HAP	Complete-case	76	NA	NA	Consistent with primary
ARDS	Complete-case	76	NA	NA	Consistent with primary

Abbreviations: **AOR**, adjusted odds ratio; **ARDS**, acute respiratory distress syndrome; **CCI**, Charlson Comorbidity Index; **CI**, confidence interval; **HAP**, hospital-acquired pneumonia; **NA**, not available; **NMBAs**, continuous neuromuscular blocking agents; **OR**, odds ratio; **RALE**, Radiographic Assessment of Lung Edema; **RR**, respiration rate.

Supplementary Table 45. Complete-Case Sensitivity Analysis of the Association of Simplified RALE Score Dichotomized at Cut-off with Hospital Mortality in Critically Ill COVID-19 Patients with Delta Variant upon Admission, Adjusted for Baseline and Mediator Confounders

Analysis Type	Sensitivity Analysis	Sample size (n)	OR (95% CI) for Hospital Mortality	p-value	Interpretation
**Exposure variable**					
Initial chest imaging					
Simplified RALE ≥5.5[a]	Complete-case	76	8.070 (1.296-50.249)	0.025	Consistent with primary
**Confounding variables**					
Inter-hospital transfers					
Endotracheal tube	Complete-case	76	NA	NA	Consistent with primary
Nasal cannula	Complete-case	76	NA	NA	Consistent with primary
Demographics					
Age (year)	Complete-case	76	NA	NA	Consistent with primary
Gender (male)	Complete-case	76	5.984 (0.987-36.292)	0.052	Consistent with primary
Comorbidities					
CCI score	Complete-case	76	NA	NA	Consistent with primary
Admission vital signs					
RR (breaths/min)	Complete-case	76	1.221 (1.019-1.464)	0.030	Consistent with primary

Admission labs					
Fibrinogen (g/L)	Complete-case	76	1.510 (0.950-2.400)	0.081	Consistent with primary
1st-day respiratory support					
Bag valve mask	Complete-case	76	NA	NA	Consistent with primary
High-flow nasal cannula	Complete-case	76	NA	NA	Consistent with primary
Adjunctive therapies					
Antiviral drugs	Complete-case	76	NA	NA	Consistent with primary
NMBAs	Complete-case	76	4.214 (0.918-19.337)	0.064	Consistent with primary
Renal replacement therapy	Complete-case	76	NA	NA	Consistent with primary
Complications					
HAP	Complete-case	76	NA	NA	Consistent with primary
ARDS	Complete-case	76	NA	NA	Consistent with primary

[a)] The numbers represent the best cut-off value determined by analysing each variable's receiver operator characteristic curve.

Abbreviations: **AOR**, adjusted odds ratio; **ARDS**, acute respiratory distress syndrome; **CCI**, Charlson Comorbidity Index; **CI**, confidence interval; **HAP**, hospital-acquired pneumonia; **NA**, not available; **NMBAs**, continuous neuromuscular blocking agents; **OR**, odds ratio; **RALE**, Radiographic Assessment of Lung Edema; **RR**, respiration rate.

Supplementary Table 46. Complete-Case Sensitivity Analysis of the Association of SOFA Score with Hospital Mortality in Critically Ill COVID-19 Patients with Delta Variant upon Admission, Adjusted for Baseline and Mediator Confounders

Analysis Type	Sensitivity Analysis	Sample size (n)	OR (95% CI) for Hospital Mortality	p-value	Interpretation
**Exposure variable**					
Admission severity of illness					
SOFA score	Complete-case	64	NA	NA	Consistent with primary
**Confounding variables**					
Inter-hospital transfers					

Endotracheal tube	Complete-case	64	NA	NA	Consistent with primary
Nasal cannula	Complete-case	64	NA	NA	Consistent with primary
Demographics					
Age (year)	Complete-case	64	NA	NA	Consistent with primary
Gender (male)	Complete-case	64	NA	NA	Consistent with primary
Comorbidities					
CCI score	Complete-case	64	NA	NA	Consistent with primary
Admission vital signs					
RR (breaths/min)	Complete-case	64	1.166 (0.969-1.402)	0.104	Consistent with primary
Admission labs					
Fibrinogen (g/L)	Complete-case	64	NA	NA	Consistent with primary
1st-day respiratory support					
Bag valve mask	Complete-case	64	NA	NA	Consistent with primary
High-flow nasal cannula	Complete-case	64	NA	NA	Consistent with primary
Adjunctive therapies					
Antiviral drugs	Complete-case	64	NA	NA	Consistent with primary
NMBAs	Complete-case	64	17.561 (3.129-98.539)	0.001	Consistent with primary
Renal replacement therapy	Complete-case	64	NA	NA	Consistent with primary
Complications					
HAP	Complete-case	64	NA	NA	Consistent with primary
ARDS	Complete-case	64	NA	NA	Consistent with primary

Abbreviations: **AOR**, adjusted odds ratio; **ARDS**, acute respiratory distress syndrome; **CCI**, Charlson Comorbidity Index; **CI**, confidence interval; **HAP**, hospital-acquired pneumonia; **NA**, not available; **NMBAs**, continuous neuromuscular blocking agents; **OR**, odds ratio; **RR**, respiration rate; **SOFA**, Sequential Organ Failure Assessment.

Supplementary Table 47. Complete-Case Sensitivity Analysis of the Association of SOFA Score Dichotomized at Cut-off with Hospital Mortality in Critically Ill COVID-19 Patients with Delta Variant upon Admission, Adjusted for Baseline and Mediator Confounders

Analysis Type	Sensitivity Analysis	Sample size (n)	OR (95% CI) for Hospital Mortality	p-value	Interpretation
**Exposure variable**					
Admission severity of illness					
SOFA score ≥ 3.5[a]	Complete-case	64	NA	NA	Consistent with primary
**Confounding variables**					
Inter-hospital transfers					
Endotracheal tube	Complete-case	64	NA	NA	Consistent with primary
Nasal cannula	Complete-case	64	NA	NA	Consistent with primary
Demographics					
Age (year)	Complete-case	64	NA	NA	Consistent with primary
Gender (male)	Complete-case	64	NA	NA	Consistent with primary
Comorbidities					
CCI score	Complete-case	64	NA	NA	Consistent with primary
Admission vital signs					
RR (breaths/min)	Complete-case	64	1.166 (0.969-1.402)	0.104	Consistent with primary
Admission labs					
Fibrinogen (g/L)	Complete-case	64	NA	NA	Consistent with primary
1st-day respiratory support					
Bag valve mask	Complete-case	64	NA	NA	Consistent with primary
High-flow nasal cannula	Complete-case	64	NA	NA	Consistent with primary
Adjunctive therapies					
Antiviral drugs	Complete-case	64	NA	NA	Consistent with primary
NMBAs	Complete-case	64	17.561 (3.129-98.539)	0.001	Consistent with primary

Renal replacement therapy	Complete-case	64	NA	NA	Consistent with primary
Complications					
HAP	Complete-case	64	NA	NA	Consistent with primary
ARDS	Complete-case	64	NA	NA	Consistent with primary

[a)] The numbers represent the best cut-off value determined by analysing each variable's receiver operator characteristic curve.

Abbreviations: **AOR**, adjusted odds ratio; **ARDS**, acute respiratory distress syndrome; **CCI**, Charlson Comorbidity Index; **CI**, confidence interval; **HAP**, hospital-acquired pneumonia; **NA**, not available; **NMBAs**, continuous neuromuscular blocking agents; **OR**, odds ratio; **RR**, respiration rate; **SOFA**, Sequential Organ Failure Assessment.

Supplementary Table 48. Complete-Case Sensitivity Analysis of the Association of APACHE II Score with Hospital Mortality in Critically Ill COVID-19 Patients with Delta Variant upon Admission, Adjusted for Baseline and Mediator Confounders

Analysis Type	Sensitivity Analysis	Sample size (n)	OR (95% CI) for Hospital Mortality	p-value	Interpretation
**Exposure variable**					
Admission severity of illness					
APACHE II score	Complete-case	60	0.806 (0.627-1.036)	0.092	Consistent with primary
**Confounding variables**					
Inter-hospital transfers					
Endotracheal tube	Complete-case	60	NA	NA	Consistent with primary
Nasal cannula	Complete-case	60	NA	NA	Consistent with primary
Demographics					
Age (year)	Complete-case	60	1.064 (0.999-1.133)	0.054	Consistent with primary
Gender (male)	Complete-case	60	8.604 (1.015-72.950)	0.048	Significant
Comorbidities					
CCI score	Complete-case	60	NA	NA	Consistent with primary
Admission vital signs					

RR (breaths/min)	Complete-case	60	1.233 (0.999-1.523)	0.051	Consistent with primary
Admission labs					
Fibrinogen (g/L)	Complete-case	60	NA	NA	Consistent with primary
1st-day respiratory support					
Bag valve mask	Complete-case	60	NA	NA	Consistent with primary
High-flow nasal cannula	Complete-case	60	NA	NA	Consistent with primary
Adjunctive therapies					
Antiviral drugs	Complete-case	60	NA	NA	Consistent with primary
NMBAs	Complete-case	60	25.904 (3.123-214.847)	0.003	Consistent with primary
Renal replacement therapy	Complete-case	60	NA	NA	Consistent with primary
Complications					
HAP	Complete-case	60	NA	NA	Consistent with primary
ARDS	Complete-case	60	NA	NA	Consistent with primary

Abbreviations: **AOR**, adjusted odds ratio; **APACHE II**, Acute Physiology and Chronic Health Evaluation II; **ARDS**, acute respiratory distress syndrome; **CCI**, Charlson Comorbidity Index; **CI**, confidence interval; **HAP**, hospital-acquired pneumonia; **NA**, not available; **NMBAs**, continuous neuromuscular blocking agents; **OR**, odds ratio; **RR**, respiration rate.

Supplementary Table 49. Complete-Case Sensitivity Analysis of the Association of APACHE II Score Dichotomized at Cut-off with Hospital Mortality in Critically Ill COVID-19 Patients with Delta Variant upon Admission, Adjusted for Baseline and Mediator Confounders

Analysis Type	Sensitivity Analysis	Sample size (n)	OR (95% CI) for Hospital Mortality	p-value	Interpretation
**Exposure variable**					
Admission severity of illness					
APACHE II score $\geq$ 11.5[a]	Complete-case	60	NA	NA	Consistent with primary
**Confounding variables**					

Inter-hospital transfers					
Endotracheal tube	Complete-case	60	NA	NA	Consistent with primary
Nasal cannula	Complete-case	60	NA	NA	Consistent with primary
Demographics					
Age (year)	Complete-case	60	NA	NA	Consistent with primary
Gender (male)	Complete-case	60	NA	NA	Consistent with primary
Comorbidities					
CCI score	Complete-case	60	NA	NA	Consistent with primary
Admission vital signs					
RR (breaths/min)	Complete-case	60	1.146 (0.964-1.361)	0.122	Consistent with primary
Admission labs					
Fibrinogen (g/L)	Complete-case	60	NA	NA	Consistent with primary
1st-day respiratory support					
Bag valve mask	Complete-case	60	NA	NA	Consistent with primary
High-flow nasal cannula	Complete-case	60	NA	NA	Consistent with primary
Adjunctive therapies					
Antiviral drugs	Complete-case	60	NA	NA	Consistent with primary
NMBAs	Complete-case	60	10.458 (2.286-47.845)	0.002	Consistent with primary
Renal replacement therapy	Complete-case	60	NA	NA	Consistent with primary
Complications					
HAP	Complete-case	60	NA	NA	Consistent with primary
ARDS	Complete-case	60	NA	NA	Consistent with primary

[a)] The numbers represent the best cut-off value determined by analysing each variable's receiver operator characteristic curve.

Abbreviations: **AOR**, adjusted odds ratio; **APACHE II**, Acute Physiology and Chronic Health Evaluation II; **ARDS**, acute respiratory distress syndrome; **CCI**, Charlson Comorbidity Index; **CI**, confidence interval; **HAP**, hospital-acquired pneumonia; **NA**, not available; **NMBAs**, continuous neuromuscular blocking agents; **OR**, odds ratio; **RR**, respiration rate.

Supplementary Table 50. Complete-Case Sensitivity Analysis of the Association of CURB-65 Score with Hospital Mortality in Critically Ill COVID-19 Patients with Delta Variant upon Admission, Adjusted for Baseline and Mediator Confounders

Analysis Type	Sensitivity Analysis	Sample size (n)	OR (95% CI) for Hospital Mortality	p-value	Interpretation
**Exposure variable**					
Admission severity of illness					
CURB-65 score	Complete-case	75	NA	NA	Consistent with primary
**Confounding variables**					
Inter-hospital transfers					
Endotracheal tube	Complete-case	75	NA	NA	Consistent with primary
Nasal cannula	Complete-case	75	NA	NA	Consistent with primary
Demographics					
Age (year)	Complete-case	75	NA	NA	Consistent with primary
Gender (male)	Complete-case	75	4.068 (0.835-19.823)	0.082	Consistent with primary
Comorbidities					
CCI score	Complete-case	75	NA	NA	Consistent with primary
Admission vital signs					
RR (breaths/min)	Complete-case	75	1.200 (1.020-1.411)	0.028	Consistent with primary
Admission labs					
Fibrinogen (g/L)	Complete-case	75	1.476 (0.934-2.335)	0.096	Consistent with primary
1st-day respiratory support					
Bag valve mask	Complete-case	75	NA	NA	Consistent with primary
High-flow nasal cannula	Complete-case	75	NA	NA	Consistent with primary
Adjunctive therapies					
Antiviral drugs	Complete-case	75	NA	NA	Consistent with primary

NMBAs	Complete-case	75	7.831 (1.919-31.956)	0.004	Consistent with primary
Renal replacement therapy	Complete-case	75	NA	NA	Consistent with primary
Complications					
HAP	Complete-case	75	NA	NA	Consistent with primary
ARDS	Complete-case	75	NA	NA	Consistent with primary

Abbreviations: **AOR**, adjusted odds ratio; **ARDS**, acute respiratory distress syndrome; **CCI**, Charlson Comorbidity Index; **CI**, confidence interval; **CURB-65**, Confusion, Urea >7 mmol/L, Respiratory Rate ≥30 breaths/min, Blood Pressure <90 mm Hg (Systolic) or <60 mm Hg (Diastolic), Age ≥65 Years; **HAP**, hospital-acquired pneumonia; **NA**, not available; **NMBAs**, continuous neuromuscular blocking agents; **OR**, odds ratio; **RR**, respiration rate.

Supplementary Table 51. Complete-Case Sensitivity Analysis of the Association of CURB-65 Score Dichotomized at Cut-off with Hospital Mortality in Critically Ill COVID-19 Patients with Delta Variant upon Admission, Adjusted for Baseline and Mediator Confounders

Analysis Type	Sensitivity Analysis	Sample size (n)	OR (95% CI) for Hospital Mortality	p-value	Interpretation
**Exposure variable**					
Admission severity of illness					
CURB-65 score ≥ 0.5[a]	Complete-case	75	NA	NA	Consistent with primary
**Confounding variables**					
Inter-hospital transfers					
Endotracheal tube	Complete-case	75	NA	NA	Consistent with primary
Nasal cannula	Complete-case	75	NA	NA	Consistent with primary
Demographics					
Age (year)	Complete-case	75	NA	NA	Consistent with primary
Gender (male)	Complete-case	75	4.068 (0.835-19.823)	0.082	Consistent with primary
Comorbidities					
CCI score	Complete-case	75	NA	NA	Consistent with primary

Admission vital signs					
RR (breaths/min)	Complete-case	75	1.200 (1.020-1.411)	0.028	Consistent with primary
Admission labs					
Fibrinogen (g/L)	Complete-case	75	1.476 (0.934-2.335)	0.096	Consistent with primary
1st-day respiratory support					
Bag valve mask	Complete-case	75	NA	NA	Consistent with primary
High-flow nasal cannula	Complete-case	75	NA	NA	Consistent with primary
Adjunctive therapies					
Antiviral drugs	Complete-case	75	NA	NA	Consistent with primary
NMBAs	Complete-case	75	7.831 (1.919-31.956)	0.004	Consistent with primary
Renal replacement therapy	Complete-case	75	NA	NA	Consistent with primary
Complications					
HAP	Complete-case	75	NA	NA	Consistent with primary
ARDS	Complete-case	75	NA	NA	Consistent with primary

[c)] The numbers represent the best cut-off value determined by analysing each variable's receiver operator characteristic curve.

Abbreviations: **AOR**, adjusted odds ratio; **ARDS**, acute respiratory distress syndrome; **CCI**, Charlson Comorbidity Index; **CI**, confidence interval; **CURB-65**, Confusion, Urea >7 mmol/L, Respiratory Rate ≥30 breaths/min, Blood Pressure <90 mm Hg (Systolic) or <60 mm Hg (Diastolic), Age ≥65 Years; **HAP**, hospital-acquired pneumonia; **NA**, not available; **NMBAs**, continuous neuromuscular blocking agents; **OR**, odds ratio; **RR**, respiration rate.

Supplementary Table 52. Complete-Case Sensitivity Analysis of the Association of First-day PaO_2/FiO_2 Ratio with Hospital Mortality in Critically Ill COVID-19 Patients with Delta Variant upon Admission, Adjusted for Baseline and Mediator Confounders

Analysis Type	Sensitivity Analysis	Sample size (n)	OR (95% CI) for Hospital Mortality	p-value	Interpretation
**Exposure variable**					
The 1st-day gas exchange					

PaO_2/FiO_2 ratio	Complete-case	51	NA	NA	Consistent with primary
**Confounding variables**					
Inter-hospital transfers					
Endotracheal tube	Complete-case	51	NA	NA	Consistent with primary
Nasal cannula	Complete-case	51	NA	NA	Consistent with primary
Demographics					
Age (year)	Complete-case	51	NA	NA	Consistent with primary
Gender (male)	Complete-case	51	NA	NA	Consistent with primary
Comorbidities					
CCI score	Complete-case	51	NA	NA	Consistent with primary
Admission vital signs					
RR (breaths/min)	Complete-case	51	1.147 (0.947-1.389)	0.161	Consistent with primary
Admission labs					
Fibrinogen (g/L)	Complete-case	51	NA	NA	Consistent with primary
1st-day respiratory support					
Bag valve mask	Complete-case	51	NA	NA	Consistent with primary
High-flow nasal cannula	Complete-case	51	NA	NA	Consistent with primary
Adjunctive therapies					
Antiviral drugs	Complete-case	51	NA	NA	Consistent with primary
NMBAs	Complete-case	51	20.411 (3.205-129.995)	0.002	Consistent with primary
Renal replacement therapy	Complete-case	51	NA	NA	Consistent with primary
Complications					
HAP	Complete-case	51	NA	NA	Consistent with primary
ARDS	Complete-case	51	NA	NA	Consistent with primary

Abbreviations: **AOR**, adjusted odds ratio; **ARDS**, acute respiratory distress syndrome; **CCI**, Charlson Comorbidity Index; **CI**, confidence interval; **HAP**, hospital-acquired pneumonia; **NA**, not available; **NMBAs**, continuous neuromuscular blocking agents; **OR**, odds ratio; **PaO_2/FiO_2**, arterial oxygen partial pressure to inspired oxygen fraction ratio; **RR**, respiration rate.

Supplementary Table 53. Complete-Case Sensitivity Analysis of the Association of First-day PaO_2/FiO_2 Ratio Dichotomized at Cut-off with Hospital Mortality in Critically Ill COVID-19 Patients with Delta Variant upon Admission, Adjusted for Baseline and Mediator Confounders

Analysis Type	Sensitivity Analysis	Sample size (n)	OR (95% CI) for Hospital Mortality	p-value	Interpretation
**Exposure variable**					
The 1st-day gas exchange					
$PaO_2/FiO_2 \geq 119.5$ mmHg[a]	Complete-case	51	NA	NA	Consistent with primary
**Confounding variables**					
Inter-hospital transfers					
Endotracheal tube	Complete-case	51	NA	NA	Consistent with primary
Nasal cannula	Complete-case	51	NA	NA	Consistent with primary
Demographics					
Age (year)	Complete-case	51	NA	NA	Consistent with primary
Gender (male)	Complete-case	51	NA	NA	Consistent with primary
Comorbidities					
CCI score	Complete-case	51	NA	NA	Consistent with primary
Admission vital signs					
RR (breaths/min)	Complete-case	51	1.147 (0.947-1.389)	0.161	Consistent with primary
Admission labs					
Fibrinogen (g/L)	Complete-case	51	NA	NA	Consistent with primary
1st-day respiratory support					
Bag valve mask	Complete-case	51	NA	NA	Consistent with primary
High-flow nasal cannula	Complete-case	51	NA	NA	Consistent with primary
Adjunctive therapies					
Antiviral drugs	Complete-case	51	NA	NA	Consistent with primary
NMBAs	Complete-case	51	20.411 (3.205-129.995)	0.001	Consistent with primary

Renal replacement therapy	Complete-case	51	NA	NA	Consistent with primary
Complications					
HAP	Complete-case	51	NA	NA	Consistent with primary
ARDS	Complete-case	51	NA	NA	Consistent with primary

[a)] The numbers represent the best cut-off value determined by analysing each variable's receiver operator characteristic curve.

Abbreviations: **AOR**, adjusted odds ratio; **ARDS**, acute respiratory distress syndrome; **CCI**, Charlson Comorbidity Index; **CI**, confidence interval; **HAP**, hospital-acquired pneumonia; **NA**, not available; **NMBAs**, continuous neuromuscular blocking agents; **OR**, odds ratio; **PaO_2/FiO_2**, arterial oxygen partial pressure to inspired oxygen fraction ratio; **RR**, respiration rate.

Supplementary Table 54. Complete-Case Sensitivity Analysis of the Association of IL-6 Level with Hospital Mortality in Critically Ill COVID-19 Patients with Delta Variant upon Admission, Adjusted for Baseline and Mediator Confounders

Analysis Type	Sensitivity Analysis	Sample size (n)	OR (95% CI) for Hospital Mortality	p-value	Interpretation
**Exposure variable**					
The 1st-day Laboratory investigations					
IL-6 level	Complete-case	67	NA	NA	Consistent with primary
**Confounding variables**					
Inter-hospital transfers					
Endotracheal tube	Complete-case	67	NA	NA	Consistent with primary
Nasal cannula	Complete-case	67	NA	NA	Consistent with primary
Demographics					
Age (year)	Complete-case	67	NA	NA	Consistent with primary
Gender (male)	Complete-case	67	NA	NA	Consistent with primary
Comorbidities					

CCI score	Complete-case	67	NA	NA	Consistent with primary
Admission vital signs					
RR (breaths/min)	Complete-case	67	NA	NA	Consistent with primary
Admission labs					
Fibrinogen (g/L)	Complete-case	67	1.601 (1.036-2.473)	0.034	Consistent with primary
1st-day respiratory support					
Bag valve mask	Complete-case	67	NA	NA	Consistent with primary
High-flow nasal cannula	Complete-case	67	NA	NA	Consistent with primary
Adjunctive therapies					
Antiviral drugs	Complete-case	67	NA	NA	Consistent with primary
NMBAs	Complete-case	67	7.618 (1.942-29.880)	0.004	Consistent with primary
Renal replacement therapy	Complete-case	67	NA	NA	Consistent with primary
Complications					
HAP	Complete-case	67	NA	NA	Consistent with primary
ARDS	Complete-case	67	NA	NA	Consistent with primary

Abbreviations: **AOR**, adjusted odds ratio; **ARDS**, acute respiratory distress syndrome; **CCI**, Charlson Comorbidity Index; **CI**, confidence interval; **HAP**, hospital-acquired pneumonia; **IL-6**, interleukin 6; **NA**, not available; **NMBAs**, continuous neuromuscular blocking agents; **OR**, odds ratio; **RR**, respiration rate.

Supplementary Table 55. Complete-Case Sensitivity Analysis of the Association of IL-6 Level Dichotomized at Cut-off with Hospital Mortality in Critically Ill COVID-19 Patients with Delta Variant upon Admission, Adjusted for Baseline and Mediator Confounders

Analysis Type	Sensitivity Analysis	Sample size (n)	OR (95% CI) for Hospital Mortality	p-value	Interpretation
**Exposure variable**					
The 1st-day Laboratory investigations					

IL-6 level ≥ 15.8 pg/mL[a]	Complete-case	67	NA	NA	Consistent with primary
**Confounding variables**					
Inter-hospital transfers					
Endotracheal tube	Complete-case	67	NA	NA	Consistent with primary
Nasal cannula	Complete-case	67	NA	NA	Consistent with primary
Demographics					
Age (year)	Complete-case	67	NA	NA	Consistent with primary
Gender (male)	Complete-case	67	NA	NA	Consistent with primary
Comorbidities					
CCI score	Complete-case	67	NA	NA	Consistent with primary
Admission vital signs					
RR (breaths/min)	Complete-case	67	NA	NA	Consistent with primary
Admission labs					
Fibrinogen (g/L)	Complete-case	67	1.601 (1.036-2.473)	0.034	Consistent with primary
1st-day respiratory support					
Bag valve mask	Complete-case	67	NA	NA	Consistent with primary
High-flow nasal cannula	Complete-case	67	NA	NA	Consistent with primary
Adjunctive therapies					
Antiviral drugs	Complete-case	67	NA	NA	Consistent with primary
NMBAs	Complete-case	67	7.618 (1.942-29.880)	0.004	Consistent with primary
Renal replacement therapy	Complete-case	67	NA	NA	Consistent with primary
Complications					
HAP	Complete-case	67	NA	NA	Consistent with primary
ARDS	Complete-case	67	NA	NA	Consistent with primary

[a)] The numbers represent the best cut-off value determined by analysing each variable's receiver operator characteristic curve.

Abbreviations: **AOR**, adjusted odds ratio; **ARDS**, acute respiratory distress syndrome; **CCI**, Charlson Comorbidity Index; **CI**, confidence interval; **HAP**, hospital-acquired pneumonia; **IL-6**, interleukin 6; **NA**, not available; **NMBAs**, continuous neuromuscular blocking agents; **OR**, odds ratio; **RR**, respiration rate.

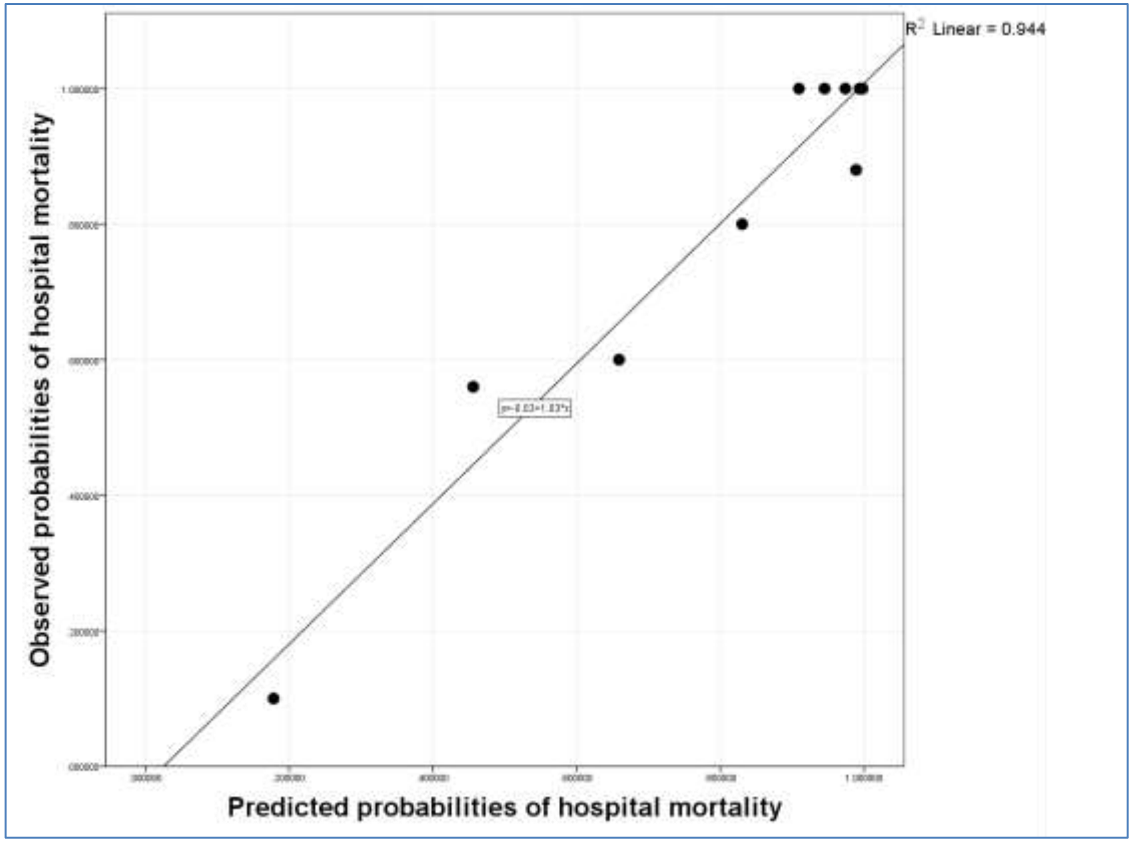

Supplementary Figure 3. Calibration plot for the multivariable logistic regression model in Supplementary Table 8. The model evaluates the association between the Simplified RALE score and hospital mortality in critically ill COVID-19 patients with the Delta variant, while adjusting for baseline confounders. Observed and predicted mortality rates are plotted. The fitted regression line is y = –0.03 + 1.03x (R^2 = 0.944), with a

calibration slope of 1.03 and an intercept of –0.03. This shows excellent calibration, as observed and predicted probabilities align across the risk range, indicating highly reliable mortality risk estimates.

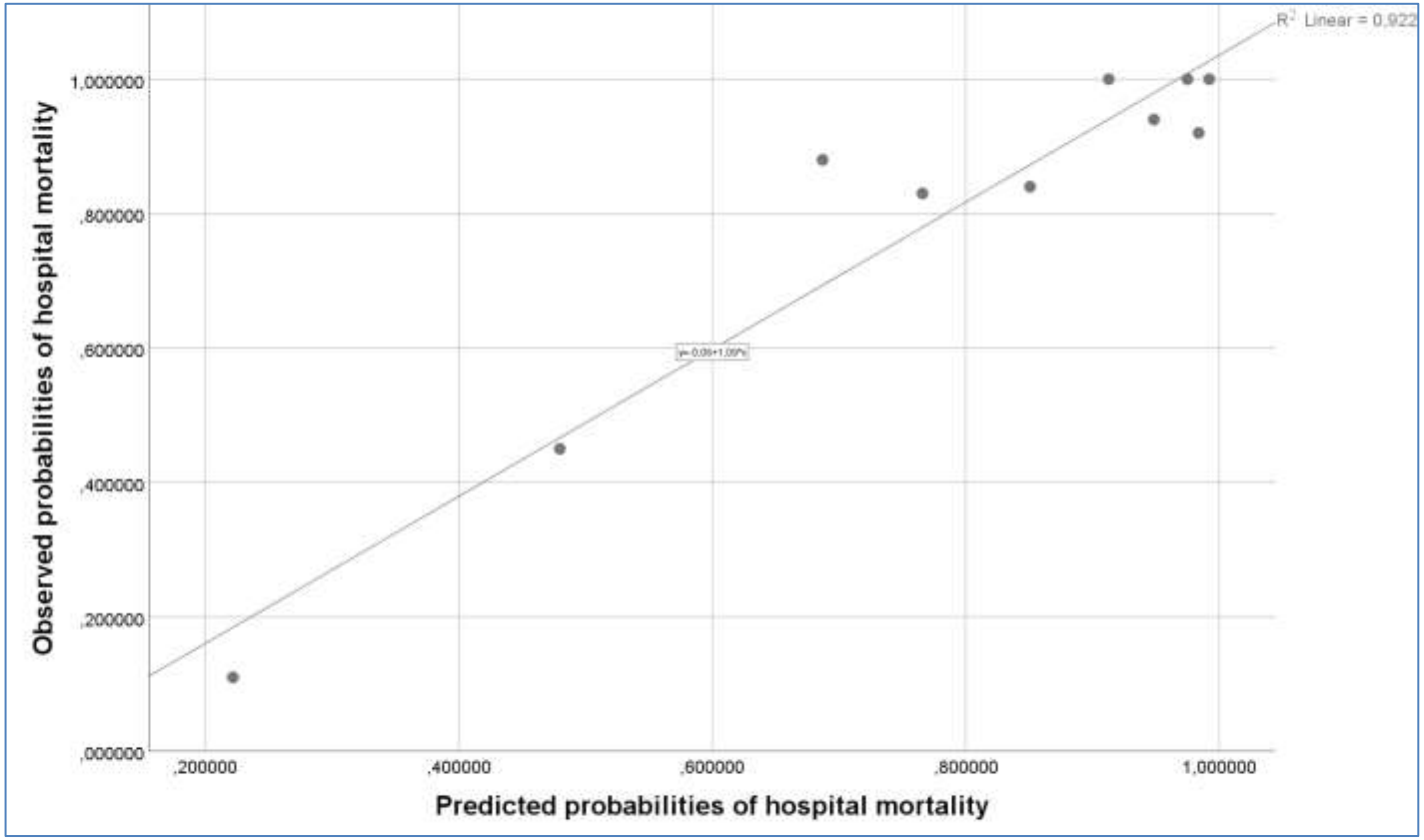

Supplementary Figure 4. Calibration plot for the multivariable logistic regression model in Supplementary Table 20. The model evaluates the association between the Simplified RALE score and hospital mortality among critically ill COVID-19 patients with the Delta variant, while

adjusting for baseline and mediator confounders. Observed hospital mortality probabilities are plotted against predicted probabilities. The fitted linear regression line is y = –0.06 + 1.09x (R^2 = 0.922), with a calibration slope of 1.09 and an intercept of –0.06. This indicates good calibration with minor underconfidence (slope slightly >1) and slight overall overprediction (intercept slightly <0), confirming that the model provides reliable mortality risk estimates.
